# SSL-VQ: vector-quantized variational autoencoders for semi-supervised prediction of therapeutic targets across diverse diseases

**DOI:** 10.1093/bioinformatics/btaf039

**Published:** 2025-01-28

**Authors:** Satoko Namba, Chen Li, Noriko Yuyama Otani, Yoshihiro Yamanishi

**Affiliations:** Department of Bioscience and Bioinformatics, Faculty of Computer Science and Systems Engineering, Kyushu Institute of Technology, Kawazu, Iizuka, Fukuoka, 820-8502, Japan; Department of Complex Systems Science, Graduate School of Informatics, Nagoya University, Chikusa, Nagoya, Aichi, 464-8601, Japan; Department of Bioscience and Bioinformatics, Faculty of Computer Science and Systems Engineering, Kyushu Institute of Technology, Kawazu, Iizuka, Fukuoka, 820-8502, Japan; Department of Complex Systems Science, Graduate School of Informatics, Nagoya University, Chikusa, Nagoya, Aichi, 464-8601, Japan; Department of Bioscience and Bioinformatics, Faculty of Computer Science and Systems Engineering, Kyushu Institute of Technology, Kawazu, Iizuka, Fukuoka, 820-8502, Japan; Department of Complex Systems Science, Graduate School of Informatics, Nagoya University, Chikusa, Nagoya, Aichi, 464-8601, Japan; Department of Bioscience and Bioinformatics, Faculty of Computer Science and Systems Engineering, Kyushu Institute of Technology, Kawazu, Iizuka, Fukuoka, 820-8502, Japan; Department of Complex Systems Science, Graduate School of Informatics, Nagoya University, Chikusa, Nagoya, Aichi, 464-8601, Japan

## Abstract

**Motivation:**

Identifying effective therapeutic targets poses a challenge in drug discovery, especially for uncharacterized diseases without known therapeutic targets (e.g. rare diseases, intractable diseases).

**Results:**

This study presents a novel machine learning approach using multimodal vector-quantized variational autoencoders (VQ-VAEs) for predicting therapeutic target molecules across diseases. To address the lack of known therapeutic target–disease associations, we incorporate the information on uncharacterized diseases without known targets or uncharacterized proteins without known indications (applicable diseases) in the semi-supervised learning (SSL) framework. The method integrates disease-specific and protein perturbation profiles with genetic perturbations (e.g. gene knockdowns and gene overexpressions) at the transcriptome level. Cross-cell representation learning, facilitated by VQ-VAEs, was performed to extract informative features from protein perturbation profiles across diverse human cell types. Concurrently, cross-disease representation learning was performed, leveraging VQ-VAE, to extract informative features reflecting disease states from disease-specific profiles. The model’s applicability to uncharacterized diseases or proteins is enhanced by considering the consistency between disease-specific and patient-specific signatures. The efficacy of the method is demonstrated across three practical scenarios for 79 diseases: target repositioning for target–disease pairs, new target prediction for uncharacterized diseases, and new indication prediction for uncharacterized proteins. This method is expected to be valuable for identifying therapeutic targets across various diseases.

**Availability and implementation:**

Code: github.com/YamanishiLab/SSL-VQ and Data: 10.5281/zenodo.14644837.

## 1 Introduction

Identifying therapeutic targets is critical in drug discovery (Emmerich *et al.* 2021). Notably, inappropriate target selection can lead to clinical trial failures (Sun *et al.* 2022). Most therapeutic targets, predominantly proteins, exert therapeutic effects through drug regulation (e.g. inhibition or activation). However, they may not necessarily align with disease susceptibility genes, causal genes, or biomarkers. For example, although most colorectal cancers exhibit adenomatous polyposis coli (APC) mutations affecting the WNT signaling pathway (Huyghe *et al.* 2019), β-catenin, a downstream biomolecule of APC, has emerged as an effective therapeutic target (Zhang and Wang 2020). Numerous studies have focused on detecting genes and proteins associated with disease progression and pathogenesis using omics data (Buphamalai *et al.* 2021). However, identified genes and proteins do not consistently translate into therapeutic targets, intensifying the challenge in drug discovery. The problem is serious especially for uncharacterized diseases without established therapeutic targets (e.g. rare diseases, intractable diseases) (Sharma *et al.* 2010).

Computational approaches, including machine learning and simulation technologies, have been successful in various drug discovery tasks, such as compound–protein interaction predictions (Nascimento *et al.* 2016), compound lead optimization (Zhou *et al.* 2019), and docking simulations with protein structures (Gentile *et al.* 2020). Conversely, limited computational approaches exist for predicting therapeutic targets. Unsupervised approaches based on genome-wide association study (GWAS) and transcriptome data from patients are popular. In GWAS-based approaches, disease susceptibility genes with single nucleotide polymorphisms (SNPs) are predicted as candidate therapeutic targets (Sabik and Farber 2017). However, it is difficult to relate SNPs directly to disease mechanism. In transcriptome-based methods, differentially expressed genes, relative to gene expression in healthy individuals, are considered candidate therapeutic targets (Ruiz-Garcia *et al.* 2010). However, these methods often yield an excess of candidates, complicating the identification of effective therapeutic targets. A supervised machine learning approach in the framework of target repositioning was developed to repurpose existing therapeutic targets for diseases that differ from the original diseases or indications (Namba *et al.* 2022). However, it cannot take into account uncharacterized diseases and proteins without known indications.

In recent years, a variety of unique omics data for proteins are becoming available. For example, protein-perturbed omics data on human cells with genetic perturbations (gene knockdown or gene overexpression) (Subramanian *et al.* 2017) would be a useful resource for exploring therapeutic target proteins, because protein perturbation profiles reflect cellular responses to protein inhibition or activation. There is an assumption that proteins with similar perturbation profiles following genetic perturbations are likely to be therapeutic targets for the same diseases. However, the information on proteins with known therapeutic indications (applicable diseases) and diseases with known therapeutic targets is limited. Thus, the lack of labeled data (known therapeutic target–disease associations) is an obstacle for the supervised learning (SL) approach. A possible solution would be to use a semi-supervised learning (SSL) approach, where models are trained using both labeled and unlabeled samples (Laine and Aila 2016). There is a strong incentive to develop an SSL framework for therapeutic target prediction.

In this study, we present a novel machine-learning method for predicting therapeutic target molecules for various diseases, leveraging multimodal vector-quantized variational autoencoders (VQ-VAEs) (Van Den Oord *et al.* 2017) within the SSL framework. The prediction integrates disease-specific and protein perturbation profiles involving gene knockdown and overexpression at the transcriptome level. Cross-cell representation learning, performed using VQ-VAE, was used to extract informative features from protein perturbation profiles across various human cell types. Additionally, cross-disease representation learning, also conducted using VQ-VAE, was used to extract informative features reflecting disease states from disease-specific profiles. To address the lack of known therapeutic target–disease associations, uncharacterized diseases and proteins were incorporated into the SSL framework. We demonstrate the utility of the proposed method in three practical scenarios: target repositioning for target–disease pairs, new target prediction for uncharacterized diseases, and new indication prediction for uncharacterized proteins.

## 2 Materials and methods

### 2.1 Overview of the proposed methods

We attempt to predict therapeutic targets based on protein–disease features extracted from protein perturbation and disease-specific profiles.

**Figure 1.**
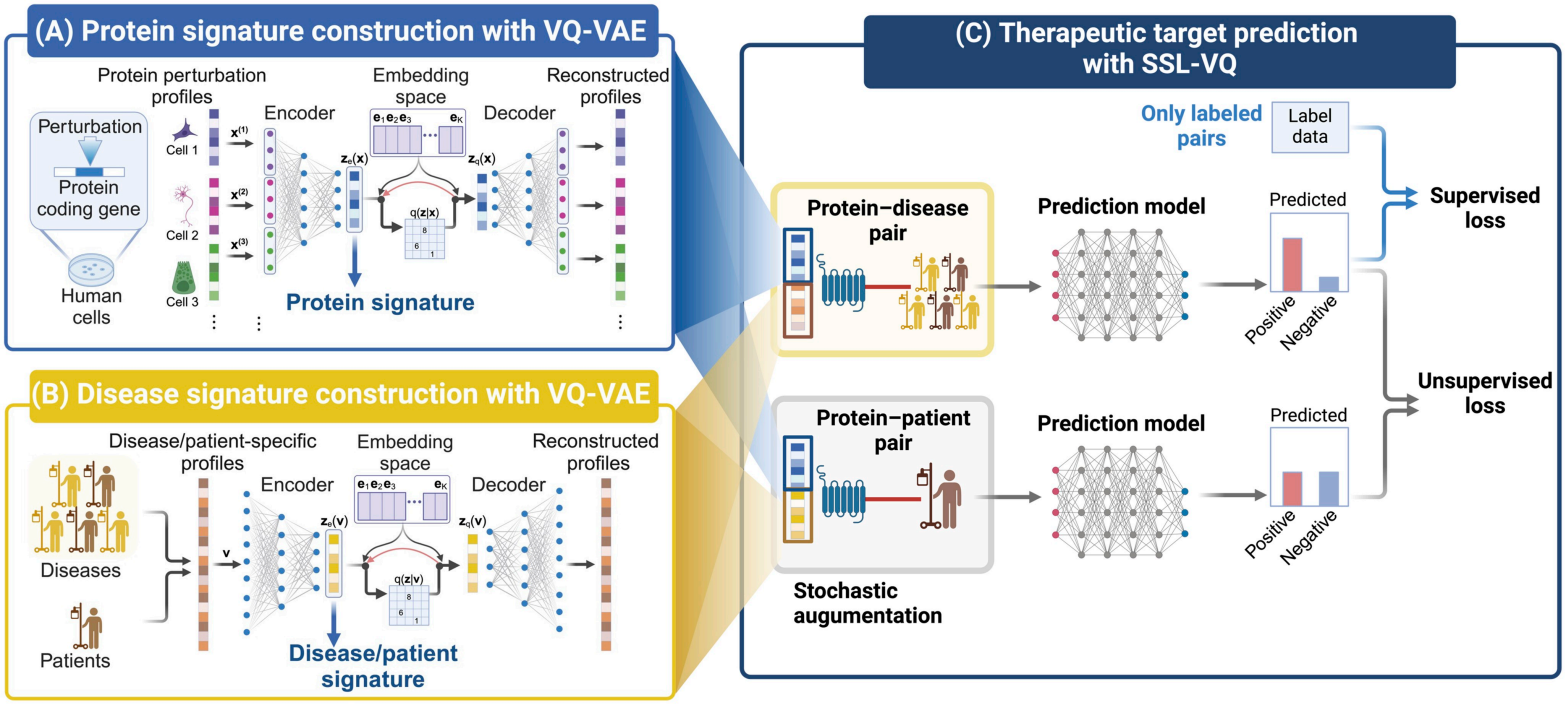
Overview of the proposed method to predict therapeutic targets for various diseases using semi-supervised learning-based neural networks with VQ-VAE signatures (SSL-VQ). (A) Cross-cell representative learning by multimodal VQ-VAE with discreate latent variables: extracting essential features (protein signatures) from protein perturbation profiles in various cell types. (B) Cross-disease representative learning by VQ-VAE: extracting crucial features (disease/patient signatures) from disease/patient-specific profiles. (C) SSL-VQ: predicting whether protein–disease pairs are therapeutic target–disease pairs (positive) or not (negative) across various diseases. Information of uncharacterized diseases and proteins are incorporated as unlabeled samples. Images were created with BioRender.com.

We modeled protein perturbation patterns based on protein perturbation profiles in various cell types by performing cross-cell representative learning using multimodal VQ-VAE with discreate latent variables, because the perturbation pattern of one protein do not continuously change to the perturbation pattern of another protein. We extracted informative features (protein signatures) across multiple cell types from protein perturbation profiles, reflecting the cellular responses to inhibitions or activations of proteins (Fig. 1A). Given that protein perturbation patterns are cell-dependent and disease pathogenesis typically involves diverse cellular interactions, we take into account various cell-specific features simultaneously.

We modeled disease states based on disease/patient-specific profiles by performing cross-disease representative learning using VQ-VAE with discreate latent variables, because the pathology-specific gene expression pattern of one disease do not continuously change to the gene expression pattern of another disease. We extracted disease/patient-specific features (disease/patient signatures) from disease/patient-specific profiles reflecting disease pathological mechanisms (Fig. 1B). Disease-specific transcriptome profiles are generally represented by multidimensional vectors containing gene expression patterns associated with the causes of disease and consequences of causal genes’ downstream reactions. VQ-VAE-based modeling of transcriptome patterns provides features with superior relevance to disease mechanisms.

We formulated the problem of therapeutic target prediction within the framework of SSL, considering the limited known therapeutic target–disease association data (Fig. 1C). We utilized protein–disease pairs, including uncharacterized diseases, as unlabeled samples. To construct a robust predictive model from heterogeneous disease-specific profiles, the model was trained to ensure close proximity between generalized disease-specific transcriptome patterns from a large number of patients with disease and transcriptome patterns from individual patients.

### 2.2 Protein perturbation transcriptome profiles

Protein perturbation profiles arising from gene knockdown or gene overexpression experiments were obtained from the L1000 (Subramanian *et al.* 2017). We constructed 4345 gene knockdown profiles across 17 cells and 4040 gene overexpression profiles across 21 cells by averaging biological replicates (Supplementary Tables S1 and S2), collectively referred to as “protein perturbation profiles.” Each of gene knockdown and overexpression profiles was represented as a feature vector, $x^{\mathrm{inh}}={\left(x_{1}^{\mathrm{inh}},x_{2}^{\mathrm{inh}},\ldots ,x_{a}^{\mathrm{inh}}\right)}^{T}$ and $x^{\mathrm{act}}={\left(x_{1}^{\mathrm{act}},x_{2}^{\mathrm{act}},\ldots ,x_{a}^{\mathrm{act}}\right)}^{T}$, respectively, where a=978 is the number of genes. For the missing values in the profiles, we performed a tensor imputation algorithm (Iwata *et al.* 2019).

### 2.3 Protein signature construction using VQ-VAE

#### 2.3.1 Protein signatures with multimodal VQ-VAE (protein multimodal VQ signatures)

To extract essential features (protein signatures) from protein perturbation profiles across various cell types, we simultaneously modeled the perturbation process in different cell types using VQ-VAE. In biological systems, each protein plays a unique role; thus, the protein functions do not continuously change from one protein to another. The cellular response to the perturbation of each protein is unique; thus, we hypothesized that protein perturbation patterns would follow a discrete distribution, leading us to use VQ-VAE, a generative model with discrete latent variables.

Consider a biological system with S proteins and C cell types. We aim to model pivotal features of protein perturbation profiles in multiple cell types. The sth protein in cth cell type is represented by a feature vector: $x_{s}^{\left(c\right)}={\left(x_{1}^{\left(c\right)},x_{2}^{\left(c\right)},\ldots ,x_{a}^{\left(c\right)}\right)}^{T}\;(s=1,\;2,\;\cdots ,\;S\;\mathrm{and}\;c=1,2,\ldots ,C)$. We construct the encoder and decoder networks for VQ-VAE, each comprising three hidden layers. The encoder’s input layer consists of C×a units to accommodate each cell’s signature $x^{\left(c\right)}$, and the second layer consists of C×512 units. The architecture of the first and second layers is cell-independent, designed as such to learn cell-specific features (Fig. 1A). The architecture between the second and third layers, and between the third and fourth layers, consists of fully connected layers, learning various cell features simultaneously. The decoder network mirrors the architecture of the encoder network. The embedding space is defined as a discrete latent space with K latent embedding vectors, denoted as $e\in R^{K\times H}$, where H represents the dimension of the embedding vectors, and information from all cell types is embedded.

Formally, the posterior categorical distribution of discrete latent variables is calculated as

(1) qz=kx=1 for k=argminj⁡zex-ej20 otherwise,

where $z_{e}(x)$ represents discrete latent variables output by the VQ-VAE encoder, mapped to K types of latent embedding vectors in latent embedding space. The latent variable $z_{q}\left(x\right),$ obtained through latent embedding, is represented as follows:

(2) zqx=ek, where k=argminj⁡zex-ej2.

Then, the latent variable $z_{q}\left(x\right)$ is used as input for the decoder. We jointly estimate all parameter sets of the encoder, decoder, and latent embedding space by minimizing the loss function as follows:

(3) L=log⁡pxzqx+sgzex-e22+βzex-sge22,

where sg represents the stop-gradient operator, which does not calculate the gradient during backpropagation. The first term denotes the reconstruction error. The second term corresponds to the L2 error, used for updating the embedding vectors. The third term is commitment loss, preventing the encoder output $z_{e}\left(x\right)$ from being updated prior to the embedding vector e. β, serving as the hyperparameter controlling the trade-off for commitment loss, was set at β = 0.25. After model training, we extracted latent variables $z_{e}\left(x\right)={\left(z_{1},z_{2},\ldots ,z_{H}\right)}^{T}$, referred to as “protein multimodal VQ signature.” The details of hyperparameters and preprocessing are shown in Supplementary Methods S1 in Supplementary Information.

#### 2.3.2 Proteins signatures with other types of VQ-VAE and VAE

As a variant of multimodal VQ-VAE, we modeled the protein perturbation process for each cell type using VQ-VAE and extracted cell-specific features from protein perturbation profiles. We constructed protein signatures by concatenating cell-specific features across all cell types, referred to as “protein cell-specific VQ signatures.”

As another variant of multimodal VQ-VAE, we used VQ-VAE based on averaged protein perturbation profiles. To extract important features from protein perturbation profiles across various cell types, the perturbation process was modeled by VQ-VAE from averaged protein perturbation profiles, referred to as “protein averaged VQ signatures.”

For comparison with VQ-VAE, we constructed “protein VAE signatures” using ordinary VAE with continues latent variables. These details are shown in Supplementary Methods S1 in Supplementary Information.

### 2.4 Disease/patient-specific transcriptome profiles

Transcriptome profiles of patients with various diseases were obtained from the CREEDs (Wang *et al.* 2016). We extracted profiles from humans for 79 diseases (Supplementary Table S4) and 14 804 genes, referring to the gene expression profiles of patients as “patient-specific profiles.” These were represented by a feature vector, $v^{\mathrm{Pat}}={\left(v_{1}^{\mathrm{Pat}},v_{2}^{\mathrm{Pat}},\ldots ,v_{b}^{\mathrm{Pat}}\right)}^{T}$, where b is the number of genes. Finally, multiple patient-specific profiles for the same disease were averaged, yielding a disease-specific profiles for each of the 79 diseases. Transcriptome profile of each disease was represented as $v^{\mathrm{Dis}}={\left(v_{1}^{\mathrm{Dis}},v_{2}^{\mathrm{Dis}},\ldots ,v_{b}^{\mathrm{Dis}}\right)}^{T}$.

### 2.5 Disease signature construction with VQ-VAE (disease/patient VQ signatures)

To extract essential features (disease signatures) from disease-specific profiles, we modeled disease states using VQ-VAE. Even if different diseases share similar pathological phenotypes, the molecular mechanisms do not change continuously from one disease to another. Therefore, we hypothesized that disease-specific transcriptome patterns would follow a discrete distribution, leading to the adoption of VQ-VAE with discrete latent variables. Additionally, given the limited number and high heterogeneity of disease-specific profiles, we enhanced model robustness by incorporating patient-specific profiles into the training process.

Given D diseases, we explore how to model important features of disease-specific and patient-specific transcriptome patterns. Each dth disease is represented as $v_{d}^{\mathrm{Dis}}={\left(v_{1}^{\mathrm{Dis}},v_{2}^{\mathrm{Dis}},\ldots ,v_{b}^{\mathrm{Dis}}\right)}^{T}\;(d=1,\;2,\;\cdots ,\;D)$, and the d'th patient with disease is represented as $v_{d'}^{\mathrm{Pat}}={\left(v_{1}^{\mathrm{Pat}},v_{2}^{\mathrm{Pat}},\ldots ,v_{b}^{\mathrm{Pat}}\right)}^{T}\left(d^{\prime }\;=\;1,2,\ldots ,D^{\prime }\right),\;$where D and D' represent the numbers of disease-specific and patient-specific profiles, respectively.

We construct encoder and decoder networks, consisting of fully connected layers (Fig. 1B). Let the output of the encoder network be $z_{e}(v)$, and the input of the decoder network be $z_{q}(v)$. We jointly estimate all parameter sets of the encoder, decoder, and latent embedding space, in a similar manner as in Section 2.3. After the model training, we extracted latent variables $z_{e}\left(v^{\mathrm{Dis}}\right)={\left(z_{1}^{\mathrm{Dis}},z_{2}^{\mathrm{Dis}},\ldots ,z_{H}^{\mathrm{Dis}}\right)}^{T}$ and $z_{e}\left(v^{\mathrm{Pat}}\right)={\left(z_{1}^{\mathrm{Pat}},z_{2}^{\mathrm{Pat}},\ldots ,z_{H}^{\mathrm{Pat}}\right)}^{T}$ for disease-specific and patient-specific profiles, respectively, referred to as “disease VQ signatures” and “patient VQ signatures,” respectively. The details of hyperparameters and preprocessing are shown in Supplementary Methods S1 in Supplementary Information.

### 2.6 Proposed methods for therapeutic target prediction

#### 2.6.1 Semi-supervised learning with VQ-VAE signatures

To overcome the limitations imposed by the scarcity of known therapeutic target–disease associations and to predict therapeutic targets for uncharacterized diseases and proteins, we used an SSL-based neural network using VQ-VAE signatures, i.e. semi-supervised learning with VQ-VAE signatures (SSL-VQ). The number of uncharacterized diseases without known therapeutic targets is large, potentially encompassing numerous diseases that could benefit from therapeutic targets.

We used the Π-model algorithm (Laine and Aila 2016) within the framework of SSL. This algorithm has been used in image classification tasks (Park *et al.* 2022), where the consistency of predictions is considered between augmented images, such as flipping and changing colors. We extended the concept of consistency to the context of disease-specific and patient-specific signatures (Fig. 1C). Given that disease-specific profiles are constructed by averaging patient-specific profiles, they are assumed to be in close proximity in the feature space. Considering the consistency can enhance model robustness, particularly given the high heterogeneity of disease-specific profiles.

Consider m known diseases with known therapeutic targets, n uncharacterized diseases without known therapeutic targets, and r known proteins with known therapeutic indications. Let set M contain the indices of known diseases, set N contain the indices of uncharacterized diseases, and set R contain the indices of known proteins. Given r×(m+n) protein–disease pairs, where $\left(r\times m\right)$ pairs are labeled pairs and (r×n) pairs are unlabeled pairs, the objective is to predict therapeutic target–disease associations. Each pair of the sth protein and dth disease is represented by a feature vector: $\Phi \left(s,d\right)$ (s ∈ R and d ∈ M∪N). Similarly, each pair of the sth protein and dth patient with disease is represented by a feature vector: $\Phi '\left(s,d\right)$ (s ∈ R and d ∈ M∪N). Notably, $\Phi \left(s,d\right)={\left[z_{e}\left(x_{s}\right),\;z_{e}\left(v_{d}^{\mathrm{Dis}}\right)\right]}^{T}$ represents a concatenated signature of protein VQ signature of the sth protein and disease VQ signature of the dth disease, whereas $\Phi '\left(s,d\right)={\left[z_{e}\left(x_{s}\right),\;z_{e}\left(v_{d}^{\mathrm{Pat}}\right)\right]}^{T}$ represents a concatenated signature of protein VQ signature of the sth protein and patient VQ signature of the dth patient with disease. In cases where a disease has multiple patient VQ signatures, one of these signatures is randomly selected.

To calculate supervised loss for labeled samples, we construct a learning set from known therapeutic target–disease associations, with m candidates for diseases and r candidates for targets. Each protein–disease pair is assigned a binary class label representing the therapeutic target–disease associations (s ∈ R and d ∈ M). Let $y_{s,d}\in \left\{0,\;1\right\}$ be the class label for the pair of the sth protein and dth disease, where $y_{s,d}=1$ if it is a therapeutic target–disease pair, while $y_{s,d}=0$ otherwise.

We construct a predictive model, denoted as $f:\;\Phi \left(s,d\right)\mapsto y$, where f is a neural network. The architecture comprises an input layer, three hidden layers, and an output layer, with all neurons being fully connected. The input layer receives protein–disease feature vectors $\Phi \left(s,d\right)$, whereas the output layer provides the probability of the sth protein and dth disease pair being a therapeutic target–disease association. The output of the (l+1)th layer is represented as follows:

(4) αl+1=gWl+1αl+εl+1,

where $W^{\left(l+1\right)}$ is the weight between the lth and (l+1)th layers, $\varepsilon^{\left(l+1\right)}$ is the bias of the (l+1)th layer, and g is the rectified linear unit function and sigmoid function in the hidden and output layers, respectively. The output value is represented by $u_{s,d}\equiv f\left(\Phi \left(s,d\right);w\right)=\alpha^{\left(4\right)}$, where ${W^{\left(1\right)},W^{\left(2\right)},\ldots ,W}^{\left(4\right)}$ are collectively denoted as w.

We estimate the weights w by minimizing the binary cross entropy weight loss as follows:

(5) LSupw=-1BProBDis∑s∈R∩BPro∑d∈M∩BDisσys,d·log⁡us,d+1-ys,d·log⁡1-us,d,

where σ is a weight for positive labels, defined as the ratio of positive labels to negative labels. Additionally, $B^{\mathrm{Pro}}$ and $B^{\mathrm{Dis}}$ are sets of protein and disease indices in the minibatch, respectively. $L^{\mathrm{Sup}}\left(w\right)$, referred to as the “supervised loss,” applies only to labeled samples.

We also introduce an unsupervised loss for unlabeled samples to regularize for the same input samples:

(6) LUnsw=ωτ12BProBDis∑s∈R∩BPro∑d∈(N∩BDis)us,d-u∼s,d2,

where $\omega \left(\tau \right)$ is the time-dependent weighting function, gradually increasing the weight of the unsupervised loss to ensure model convergence. ${\tilde{u}}_{s,d}\equiv f\left(\Phi '\left(s,d\right);w\right)$ is the prediction result of input signature $\Phi '\left(s,d\right)$, the concatenated signature of protein and patient VQ signatures. Notably, ${\tilde{u}}_{s,d}$ is a stochastic variable of $u_{s,d}$ owing to network dropout and the use of patient VQ signature rather than disease VQ signature. Training these outputs to be in close proximity enhances robustness, given limited number of labeled samples.

Following the addition of supervised and unsupervised loss components, the final loss function is represented as follows:

(7) L=LSupw+LUnsw.

A grid search was performed to determine the optimal hyper parameters. The details of hyperparameters are shown in Supplementary Methods S1 in Supplementary Information. The Π-model algorithm is shown in Table 1.

**Table 1.** Algorithm of the Π-model.

**Require**: M= set of known disease indices with known labels **Require**: N= set of uncharacterized disease indices without known labels **Require:** R= set of known protein indices with known labels **Require:** $\Phi \left(s,d\right)=$ feature vector of protein–disease pair, s∈R and d∈(M∪N) **Require:** $\Phi '\left(s,d\right)=$ feature vector of protein–patient pair, s∈R and d∈(M∪N) **Require:** $y_{s,d}=$ labels for labeled inputs s∈R and d∈M **Require:** $\omega \left(\tau \right)=$ unsupervised weight ramp-up function **Require:** $f_{w}(\Phi \left(s,d\right))=$ stochastic neural network with trainable parameters w	
**Process:**	
** for** i in [1, num_epochs] **do**	
** for** each minibatch $B^{\mathrm{Pro}}$, $B^{\mathrm{Dis}}\;$**do**	
** ** $u_{s\in \left(R\cap B^{\mathrm{Pro}}\right),d\in \left\{\left(M\cup N\right)\cap B^{\mathrm{Dis}}\right\}}\leftarrow f\left(\Phi \left(s,d\right);w\right)$	Evaluate network outputs for protein–disease inputs
** ** ${\tilde{u}}_{s\in (R\cap B^{\mathrm{Pro}}),d\in \left\{N\cap B^{\mathrm{Dis}}\right\}}\leftarrow f\left(\Phi '\left(s,d\right);w\right)$	Again, with different dropout and protein–patient inputs
** ** $L\leftarrow L^{\mathrm{Sup}}\left(w\right)+L^{\mathrm{Uns}}\left(w\right)$	Supervised and unsupervised loss components
** **Update w using the ADAM optimizer	Update network parameters
** end for**	
** end for** ** return** w	

#### 2.6.2 Supervised learning with VQ-VAE signatures

To examine the effect of SSL considering information on uncharacterized diseases and uncharacterized proteins, we tested a SL framework. Only labeled protein–disease pairs were used for training. The loss function in SL is represented as in Equation (5). The neural network architecture remains the same as that in SSL-VQ, and hyperparameters were determined through 5-fold cross validation. The method is referred to as supervised learning with VQ-VAE signatures “SL-VQ.”

### 2.7 Baseline methods for therapeutic target prediction

The multitask learning method incorporates protein perturbation profiles and disease similarities to predict therapeutic targets (Namba *et al.* 2022). The method is referred to as “Multitask.”

Information regarding disease-associated SNPs is used to identify therapeutic targets (Shastry 2007, Sabik and Farber 2017). The underlying assumption of this approach is that diseases result from functional changes in proteins encoded by genes containing SNPs in their coding regions, regarding these genes potential therapeutic targets. We constructed three types of disease-specific SNP profiles: “SNP-PV,” “SNP-LD,” and “SNP-eQTL.”

In SNP-PV, when a gene had multiple SNPs or was reported by multiple GWASs, we averaged its p-values. We used $-\log(p^{\mathrm{SNP}})$ values as predictive scores. Genes with SNPs associated with a disease were considered to be candidate therapeutic targets. In SNP-LD, SNPs were mapped to genes using FUMA (Watanabe *et al.* 2017), considering linkage disequilibrium (LD). We then calculated the *P*-value of each gene as a prediction score based on *P*-values of SNPs by meta-analysis. In SNP-eQTL, for genes with multiple expression quantitative trait loci (eQTLs), we summed their eQTL values obtained from GTEx (Lonsdale *et al.* 2013) (v8). Genes with highly positive and negative eQTL values were considered as candidate inhibitory and activatory targets, respectively.

### 2.8 Experimental setup in three scenarios in practice

We simulate practical applications of therapeutic target prediction by considering three scenarios: (i) target repositioning for target–disease pairs, (ii) new target prediction for uncharacterized diseases, and (iii) new indication prediction for uncharacterized proteins.

#### 2.8.1 Target repositioning for target–disease pairs

We aim to detect missing associations between known therapeutic target proteins and diseases, using information on known therapeutic target proteins. Hence, we performed a 5-fold pair-wise cross-validation. First, we randomly partitioned target–disease pairs in the gold standard dataset (see Section 2.9) into five roughly equal subsets, each serving as a test set in turn. Subsequently, we trained a predictive model on the remaining four subsets and computed prediction scores for target–disease pairs in the test set. Finally, we evaluated the prediction accuracy over the five folds.

#### 2.8.2 New target prediction for uncharacterized diseases

We aim to predict new therapeutic target proteins for uncharacterized diseases without known therapeutic targets. Initially, we trained a predictive model on the gold standard set, and the model was then applied to uncharacterized diseases. After computing prediction scores for target–uncharacterized disease pairs, we validated the prediction results through a manually curated set of therapeutic targets for the uncharacterized diseases (see Section 2.9) and evaluated prediction accuracy.

#### 2.8.3 New indication prediction for uncharacterized proteins

We aim to predict new therapeutic indications (applicable diseases) for uncharacterized proteins without known therapeutic indications. Initially, we trained a predictive model on the gold standard set, and the model was then applied to uncharacterized proteins. After computing prediction scores for uncharacterized protein–disease pairs, we validated the prediction results by a manually curated set of therapeutic indications for the uncharacterized proteins (see Section 2.9), and evaluated the accuracy.

### 2.9 Therapeutic target data

The information on therapeutic target molecules was sourced from a previous study (Namba *et al.* 2022). In total, 529 target−disease associations, comprising 225 inhibitory targets and 32 diseases, were used as gold standard inhibitory target data. Additionally, 45 target−disease associations, involving 37 activatory targets and 16 diseases, were used as gold standard activatory target data. This dataset served as the “gold standard set” for the “target repositioning for target–disease pairs” scenario.

Independent of this dataset, we prepared new therapeutic target data for predicting uncharacterized diseases and proteins through manually curation from medical monographs (Papadakis *et al.* 2014) and recent literature (Supplementary Tables S5–S9). Specifically, for uncharacterized disease prediction, 30 target−disease associations involving 30 inhibitory targets and 6 diseases were used as test data of inhibitory target prediction. Additionally, 34 target−disease associations, encompassing 26 activatory targets and 16 diseases, were used as test data of activatory target prediction. This dataset was used for the “new target prediction for uncharacterized diseases” scenario (Section 2.8.2).

For uncharacterized protein prediction, 73 target−disease associations, involving 63 inhibitory targets and 24 diseases, were used as test data of inhibitory target prediction. Additionally, 32 target−disease associations, comprising 21 activatory targets and 18 diseases, were used as test data of activatory target prediction. This dataset was used for the “new indication prediction for uncharacterized proteins” scenario (Section 2.8.3).

Note that there is no overlap among the three therapeutic target datasets for individual scenarios.

### 2.10 Performance evaluation procedure

We used the area under the receiver operating characteristic (ROC) curve (AUC) as an accuracy measure. ROC curves were generated to access the performance of classifiers over all possible cutoffs. The true positive rates (TPRs) were plotted against the false positive rates (FPRs). AUC scores, ranging from 0 to 1.0, provide a quantitative measure of classifier performance, where 1.0 indicates perfect inference (100% TPR, 0% FPR), and 0.5 represents random inference.

## 3 Results

### 3.1 Performance evaluation of target repositioning for target–disease pairs

We evaluated the performance of SSL-VQ in target repositioning, a scenario involving the repositioning of existing therapeutic targets to other diseases. We performed pair-wise cross-validation using the gold standard dataset (see the “target repositioning for target–disease pairs” scenario in the Section 2.8.1). To examine the effect of VQ-VAE, we also tested SSL and SL with signatures constructed by ordinary VAE (referred to as SSL-VAE and SL-VAE, respectively; see the Section 2.3.2). A performance comparison was conducted between proposed (SSL-VQ, SSL-VAE, SL-VQ and SL-VAE) and baseline (SNP-PV, SNP-LD, SNP-eQTL, and Multitask) methods.

**Figure 2.**
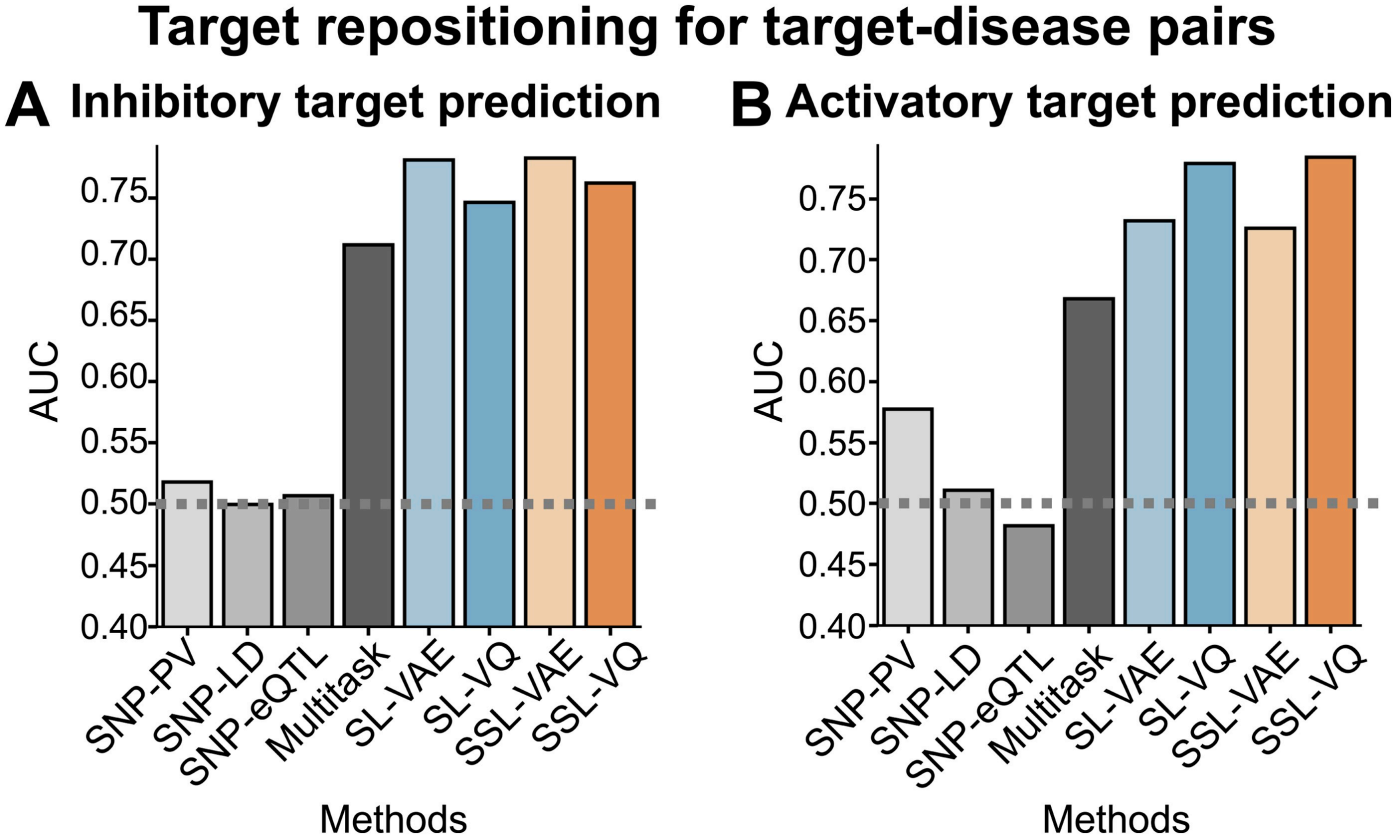
Performance evaluation of target repositioning for target–disease pairs. (A) Comparison of proposed (SSL-VQ, SSL-VAE, SL-VQ, and SL-VAE) and baseline (SNP-PV, SNP-LD, SNP-eQTL, and Multitask) methods for predicting inhibitory targets for 33 diseases and 225 proteins. (B) As described in (A), but for activatory target predictions involving 16 diseases and 37 proteins.

Figure 2A and B shows the results of performance evaluations for inhibitory and activatory target predictions, respectively. SSL-VQ showed stable accuracy for both inhibitory target predictions and activatory target predictions. Although SL-VQ was slightly less accurate compared with SSL-VQ, it outperformed the baseline methods. SSL-VAE was less accurate compared with SSL-VQ for activatory target prediction, but SSL-VAE was more accurate compared with SSL-VQ for inhibitory target prediction. SNP-PV was modestly accurate in predicting activatory targets; SNP-LD considering LD, and SNP-eQTL did not do so well. These methods are useful for identifying disease susceptibility genes, but susceptibility genes are not necessarily therapeutic targets, which may explain this observation. These results suggest that SSL-VQ excels in predicting inhibitory and activatory targets separately with stable accuracy, outperforming baseline methods by considering fused patterns of protein perturbations across various cell types.

### 3.2 Performance evaluation of new target predictions for uncharacterized diseases

The performance of SSL-VQ in predicting therapeutic targets for uncharacterized diseases was evaluated. Specifically, models were trained using the gold standard dataset containing diseases with at least one known therapeutic target and subsequently evaluated through their application to uncharacterized diseases without known therapeutic targets (see the “new target prediction for uncharacterized diseases” scenario in the Section 2.8.2). SSL-VQ was compared with SSL-VAE, SL-VQ, SL-VAE, SNP-PV, SNP-LD, SNP-eQTL, and Multitask.

**Figure 3.**
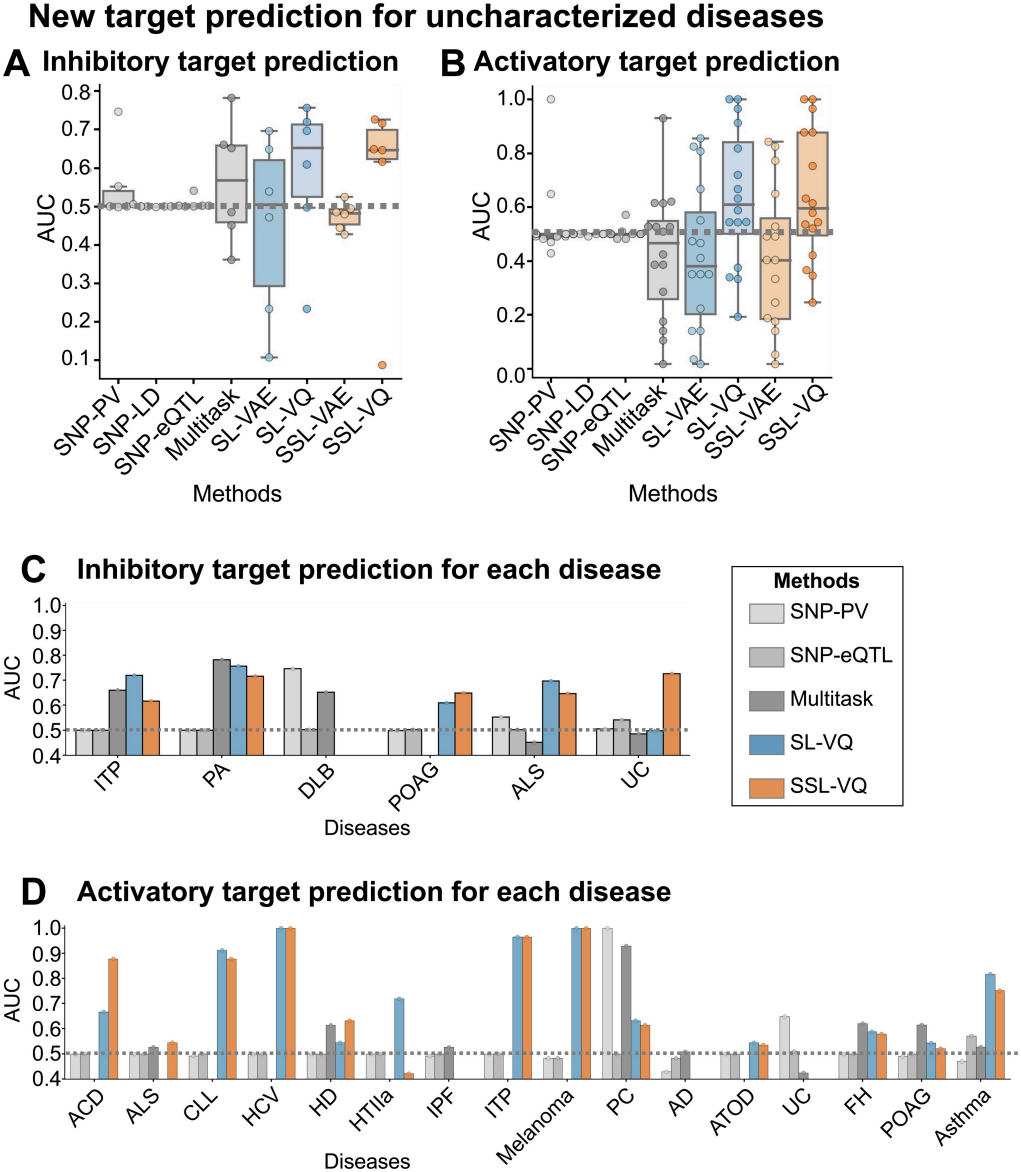
Performance evaluation of new target predictions for uncharacterized diseases. (A) Comparison of proposed (SSL-VQ, SSL-VAE, SL-VQ, and SL-VAE) and baseline (SNP-PV, SNP-LD, SNP-eQTL, and Multitask) methods for predicting inhibitory targets involving 6 diseases and 30 proteins. Boxplots represent AUC score distributions for each disease. (B) As described in (A), but for activatory target predictions involving 16 diseases and 26 proteins. (C) As described in (A), but showing bar graphs representing inhibitory target predictions for each disease. (D) As described in (C), but for activatory target predictions. Disease abbreviations: IPF, idiopathic pulmonary fibrosis; FH, familial hypercholesterolemia.

Figure 3A and B shows performance evaluations for predicting inhibitory and activatory targets for uncharacterized diseases, respectively. SSL-VQ and SL-VQ exhibited superior accuracy for inhibitory target predictions compared with the baseline methods. For activatory target prediction, SSL-VQ significantly outperformed baseline methods. SL-VQ also performed better than baseline methods (Multitask, $P=8.0\times 10^{-2}$; SNP-eQTL, $P=3.7\times 10^{-2}$; SNP-PV, $P=8.8\times 10^{-2}$). The accuracy of SSL-VAE and SL-VAE was poor, which indicates that feature extraction by VQ-VAE with discreate variables, rather than VAE with continuous variables, is more useful, especially for prediction for uncharacterized diseases. These results suggest that it is useful to incorporate information of uncharacterized diseases and proteins as unlabeled samples in SSL-VQ.

Detailed performance comparisons for each disease revealed that SSL-VQ or SL-VQ was more accurate in four of six diseases for inhibitory target prediction (Fig. 3C). Compared with baseline methods, SSL-VQ exhibited higher accuracy in primary open angle glaucoma (POAG), amyotrophic lateral sclerosis (ALS), and ulcerative colitis (UC). Compared with baseline methods, SL-VQ showed higher accuracy in immune thrombocytopenic purpura (ITP), POAG, and ALS. SNP-PV and Multitask were more accurate for dementia with Lewy bodies (DLB) and pituitary adenomas (PA), respectively. We then elaborated the details of the prediction results using independent resources. SSL-VQ predicted TNFA with a higher rank for UC, an intractable disease without curative treatment. In UC, TNFA is excessively produced, triggering an inflammatory response in the colon through macrophage activation and neutrophil migration to the vascular endothelium (Oshitani 2016). The antibody drug infliximab, which inhibits TNFA, has been shown to improve symptoms in patients with moderate to severe UC (Cui *et al.* 2021). These results suggest that SSL-VQ can effectively predict inhibitory targets for uncharacterized diseases, including intractable diseases.

For activatory target prediction, SSL-VQ outperformed the baseline methods, particularly for allergic contact dermatitis (ACD), ALS, chronic lymphocytic leukemia (CLL), hepatitis C (HCV), Huntington disease (HD), ITP, melanoma, atopic dermatitis (ATOD), and asthma (Fig. 3D). SL-VQ also outperformed the baseline methods, particularly for ACD, CLL, HCV, hyperlipoproteinemia type IIa (HTIIa), ITP, melanoma, ATOD, and asthma. SNP-PV was more accurate for pancreatic cancer (PC) and UC. We then elaborated the details of the prediction results using independent resources. SSL-VQ predicted NR3C1, a glucocorticoid receptor (GR), with a higher rank for ACD. GR activation contributes to treatment of ACD (Omura and Tamura 2015), with NR3C1 serving as an activatory target for betamethasone, cortisol, dexamethasone, prednisolone, and triamcinolone (Kawai 2013). These findings suggest that SSL-VQ can effectively predict activatory targets for uncharacterized diseases without known activatory targets.

### 3.3 Performance evaluation of new indication predictions for uncharacterized proteins

We evaluated the prediction accuracy of SSL-VQ in predicting therapeutic indication for uncharacterized proteins, suggesting that SSL-VQ can predict new applicable diseases for various uncharacterized proteins. The details are shown in Supplementary Results S1 in Supplementary Information.

### 3.4 Feature extraction processes from multiple cell types

We explored the impact of feature extraction processes from multiple cell types on prediction accuracy. The results suggest that multimodal VQ-VAE learning various cell types simultaneously are more useful for all practical scenarios rather than learning each cell types separately. These details are shown in Supplementary Results S1 in Supplementary Information.

### 3.5 Biological interpretation of newly predicted therapeutic targets for uncharacterized diseases

We comprehensively predicted new therapeutic targets for all protein–disease pairs (inhibitory targets: 343 255 pairs involving 4345 proteins and 79 diseases; activatory targets: 319 160 pairs involving 4040 proteins and 79 diseases) using SSL-VQ (Supplementary Tables S12 and S13). Moreover, we elaborated the validity of the predicted associations through a literature review.

**Figure 4.**
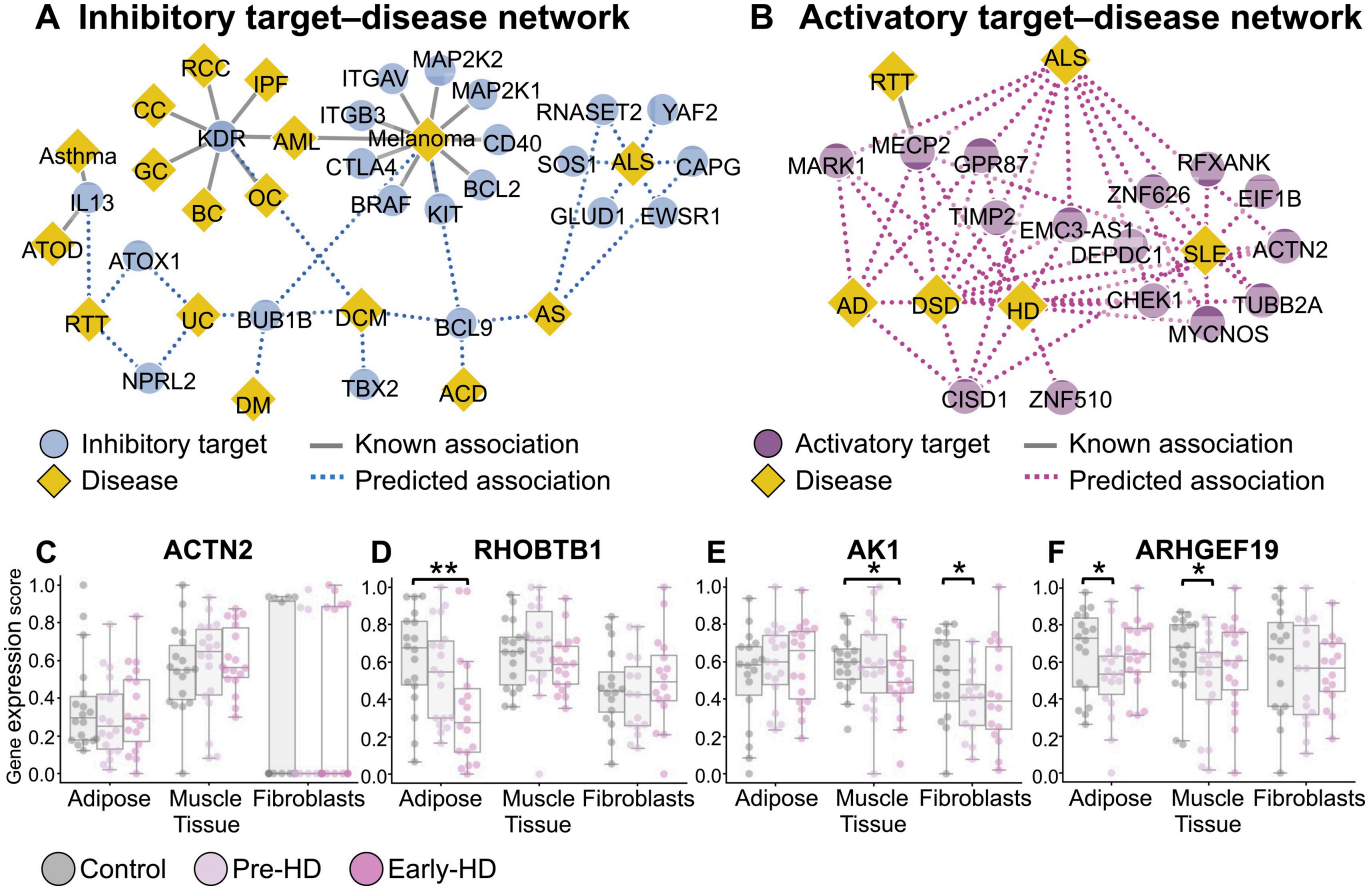
Biological interpretation of newly predicted therapeutic targets for uncharacterized diseases. (A) Part of the newly predicted inhibitory target–disease association network achieved using SSL-VQ. Circles and diamonds denote inhibitory targets and diseases, whereas gray and blue lines represent known and predicted associations, respectively. (B) As described in (A), but for activatory targets. (C) Comparison of gene expression scores of the predicted activatory target for HD, *ACTN2*, among control, pre-HD, and early-HD groups in an independent cohort. Boxes represent gene expression score distributions. The asterisk represents significance. (D) As described in (C), but for *RHOBTB1*. (E) As described in (C), but for *AK1*. (F) As described in (C), but for *ARHGEF19*. Disease abbreviations: AML, acute myeloid leukemia; AS, Alpers syndrome; BC, breast cancer; CC, colorectal cancer; DM, distal myopathy; DSD, 46, XY disorder of sex development; GC, gastric cancer; OC, ovarian cancer; RCC, renal cell carcinoma.

Figure 4A shows a portion of the newly predicted inhibitory target–disease association network. We focused on associations for uncharacterized diseases without known inhibitory targets. For dilated cardiomyopathy (DCM), a progressive degenerative disease of the myocardium that is intractable with no curative treatment except heart transplantation, BUB1B was predicted as an inhibitory target. BUB1B upregulation is associated with mitotic dysregulation, which contributes to DCM progression (Zhou *et al.* 2016). For Rett syndrome (RTT), another uncharacterized and intractable disease without known targets, IL-13 was predicted as an inhibitory target. IL-13 upregulation is observed in RTT (Pecorelli *et al.* 2016), with Th2 known to produce IL-13, and a Th2-shifted balance increasing Th2-producing cytokine levels (Leoncini *et al.* 2015). For ALS, an uncharacterized disease, EWSR1 and CAPG were predicted as inhibitory targets. Patients with ALS exhibit missense mutations in EWSR1, and EWSR1 localization in motor neurons is implicated in the ALS mechanism (Couthouis *et al.* 2012). Notably, changes in CAPG protein levels occur in the cerebrospinal fluid of patients with ALS (Oeckl *et al.* 2020). Collectively, these findings indicate that SSL-VQ has the potential to predict promising inhibitory targets for uncharacterized diseases.

Figure 4B shows a segment of the newly predicted activatory target–disease association network. We focused on associations for uncharacterized diseases without known activatory targets. For HD, an uncharacterized disease, ACTN2 was predicted as an activatory target, aligning with its significant downregulation in both HD model mice and human patients with HD (Becanovic *et al.* 2010). For ALS, MECP2 was predicted as an activatory target. *MECP2* is a target gene of FUS, a DNA-binding protein with ALS-specific mutations, and MECP2 protein levels are known to be reduced in ALS mutant derivatives (Coady and Manley 2015). For systemic lupus erythematosus (SLE), another uncharacterized disease, TUBB2A was predicted as an activatory target, consistent with its significant downregulation in patients with SLE ($P=5.0\times 10^{-5}$) (Zhang *et al.* 2019). These results highlight the potential of SSL-VQ for predicting promising activatory targets for uncharacterized diseases.

Finally, we examined the validity of predicted activatory targets for HD using independent cohort data. The cohort data consisted of three groups, i.e. control (n=24), before disease onset (pre-HD; n=23), and patients with early HD (early-HD; n=21), as well as three tissues, namely adipose, muscle, and fibroblasts (Neueder *et al.* 2022) (Supplementary Methods S1). We compared the gene expression levels of predicted targets with high prediction ranks (Supplementary Table S13), i.e. *ACTN2*, *RHOBTB1*, *AK1*, and *ARHGEF19*, among the control, pre-HD, and early-HD groups. *ACTN2* exhibited no significant changes (Fig. 4C). ACTN2 is reportedly downregulated in the striatum (Becanovic *et al.* 2010), a distinction from the tissues in this cohort, which may explain the disparate results. In contrast, *RHOBTB1* was significantly downregulated in early-HD adipose $(P=2.6\times 10^{-3}$) (Fig. 4D). RHOBTB1 interacts directly with SETD2, a histone lysine methyltransferase implicated in HD pathogenesis (Kumar *et al.* 2023). *AK1* was downregulated in pre-HD fibroblasts ($P=2.8\times 10^{-2})$ and early-HD muscle ($P=4.5\times 10^{-2}$) (Fig. 4E). *ARHGEF19* was downregulated in pre-HD muscle ($P=3.2\times 10^{-2}$) and pre-HD adipose ($P=4.8\times 10^{-2}$) (Fig. 4F), indicating its potential involvement in disease pathogenesis and possible designation as an activatory target prior to disease onset. Collectively, these results affirm the validity of the predicted activatory targets for HD.

## 4 Discussion

In this study, we developed SSL-VQ to predict therapeutic targets for various diseases by leveraging protein perturbation profiles across multiple cell types and disease-specific profiles at the transcriptome level. Using VQ-VAE, we conducted cross-cell representation learning to extract fused features from protein perturbation profiles in diverse cell types. Additionally, we performed cross-disease representation learning to extract crucial features reflecting disease states from disease-specific profiles. The originality lies in the incorporation of information regarding diseases without known therapeutic targets or proteins without known indications, the consideration of consistency between disease-specific and patient-specific VQ signatures, and the applicability toward uncharacterized diseases and proteins. We demonstrated the utility of SSL-VQ in target repositioning, predicting therapeutic targets for uncharacterized diseases, and predicting applicable diseases for uncharacterized proteins. Our method is expected to facilitate the identification of therapeutic target across various diseases.

## 5 Conclusion

VQ-VAE worked better than ordinary VAE with continuous latent variables (Supplementary Results S1). VQ-VAE-based dimension reduction of transcriptome profiles enabled to perform SSL with many unlabeled pairs, which was not possible when using the original transcriptome profiles. However, VQ-VAE is known to suffer from codebook collapse when the original feature vectors are numerically similar. We addressed this issue by incorporating the LeakyReLU into activation function, but stochastically quantized VAE (Takida *et al.* 2022) may be an alternative solution.

## Supplementary Material

btaf039_Supplementary_Data

## Data Availability

The data underlying this article are available in Zenodo, at [https://doi.org/10.5281/zenodo.14644837].
