# Supplementary material for: SSL-VQ: vector-quantized variational autoencoders for semi-supervised prediction of therapeutic targets across diverse diseases
Source: Bioinformatics. 2025 Jan 28;41(2):btaf039. doi: 10.1093/bioinformatics/btaf039 (PMC11842052; doi:10.1093/bioinformatics/btaf039)
Supplement: btaf039_Supplementary_Data [file btaf039_supplementary_data.pdf]

Supplementary information for

SSL-VQ: vector-quantized variational autoencoders for semi-supervised prediction of therapeutic targets across diverse diseases

Satoko Namba[1,2], Chen Li[1,2], Noriko Otani[1,2] and Yoshihiro Yamanishi[1,2*]

[1]Department of Bioscience and Bioinformatics, Faculty of Computer Science and Systems Engineering, Kyushu Institute of Technology, Iizuka, Fukuoka 820-8502, Japan,
[2]Graduate School of Informatics, Nagoya University, Chikusa, Nagoya 464-8601, Japan.

*corresponding author: Yoshihiro Yamanishi (yamanishi@i.nagoya-u.ac.jp)

Table of contents

Table of contents 2

Supplementary Methods 6

1. Protein signatures with multimodal VQ-VAE (protein multimodal VQ signatures) 6
2. Proteins signatures with cell-specific VQ-VAE (protein cell-specific VQ signatures) 7
3. Protein signatures with averaged VQ-VAE (protein averaged VQ signatures) 8
4. Protein signatures with averaged VAE (protein averaged VAE signatures) 9
5. Disease/patient-specific signatures with VQ-VAE (disease/patient VQ signatures) 10
6. Disease/patient-specific signatures with VAE (disease/patient VAE signatures) 12
7. Semi-supervised learning-based neural network with VQ-VAE signatures (SSL-VQ) 13
8. Protein perturbation transcriptome profiles 14
9. Data completion method 15
10. Disease/patient-specific transcriptome profiles 16
11. Validation of predicted therapeutic targets for Huntington's disease (HD) 17

Supplementary Results 18

1. Performance evaluation of new indication predictions for uncharacterized proteins 18
2. Feature extraction processes from multiple cell types 20
3. Performance comparison between VQ-VAE and VAE signatures 22
4. Codebook collapse in VQ-VAE 24
5. Biological interpretation of newly predicted therapeutic targets for Huntington's diseases 25
6. Performance evaluation of SSL-VQ based on the F1 measure 26
7. Performance comparisons when trained on datasets with different amounts of unlabeled samples 28
8. Performance evaluation of the proposed methods for common diseases and rare diseases 30
9. Model interpretability and biological relevance of multimodal VQ-VAE 31
10. Performance evaluation of SSL-VQ for each disease and protein 32

Supplementary Discussion 33

Supplementary References 35

Supplementary Figures 36

Supplementary Fig. S1: Performance evaluation of target repositioning for target-disease pairs based on the F1 measure. 36
Supplementary Fig. S2: Performance evaluation of new target predictions for uncharacterized diseases based on the F1 measure. 37
Supplementary Fig. S3: Performance evaluation of new indication prediction for uncharacterized proteins based on AUC. 38
Supplementary Fig. S4: Performance evaluation of new indication prediction for uncharacterized proteins based on the F1 measure. 39
Supplementary Fig. S5: Performance evaluation of proposed methods for predicting therapeutic indications for uncharacterized proteins. 40
Supplementary Fig. S6: Performance evaluation of predicting therapeutic indications for uncharacterized proteins for each protein class. 41
Supplementary Fig. S7: Performance comparison between feature extraction processes from multiple cells based on AUC. 42
Supplementary Fig. S8: Performance comparison between VAE signatures and VQ-VAE signatures. 43
Supplementary Fig. S9: Performance comparisons based on AUC when trained on datasets with different amounts of unlabeled samples. 44
Supplementary Fig. S10: Performance evaluation of the proposed methods for common diseases and rare diseases. 45
Supplementary Fig. S11: Distribution of original and reconstructed gene knockdown signatures of A375. 46
Supplementary Fig. S12: Distribution of original and reconstructed gene knockdown signatures of A549. 47
Supplementary Fig. S13: Distribution of original and reconstructed gene knockdown signatures of ASC. 48
Supplementary Fig. S14: Distribution of original and reconstructed gene knockdown signatures of HA1E. 49
Supplementary Fig. S15: Distribution of original and reconstructed gene knockdown signatures of HCC515. 50
Supplementary Fig. S16: Distribution of original and reconstructed gene knockdown signatures of HEPG2. 51
Supplementary Fig. S17: Distribution of original and reconstructed gene knockdown signatures of HEPG2. 52
Supplementary Fig. S18: Distribution of original and reconstructed gene knockdown signatures of HT29. 53
Supplementary Fig. S19: Distribution of original and reconstructed gene knockdown signatures of MCF7. 54
Supplementary Fig. S20: Distribution of original and reconstructed gene knockdown signatures of NPC. 55
Supplementary Fig. S21: Distribution of original and reconstructed gene knockdown signatures of PC3. 56
Supplementary Fig. S22: Distribution of original and reconstructed gene knockdown signatures of VCAP. 57
Supplementary Fig. S23: Distribution of original and reconstructed gene overexpression signatures of A375. 58
Supplementary Fig. S24: Distribution of original and reconstructed gene overexpression signatures of A549. 59
Supplementary Fig. S25: Distribution of original and reconstructed gene overexpression signatures of HA1E. 60
Supplementary Fig. S26: Distribution of original and reconstructed gene overexpression signatures of HCC515. 61

Supplementary Fig. S27: Distribution of original and reconstructed gene overexpression signatures of HEK293T. 62
Supplementary Fig. S28: Distribution of original and reconstructed gene overexpression signatures of HEPG2. 63
Supplementary Fig. S29: Distribution of original and reconstructed gene overexpression signatures of HT29.... 64
Supplementary Fig. S30: Distribution of original and reconstructed gene overexpression signatures of MCF7... 65
Supplementary Fig. S31: Distribution of original and reconstructed gene overexpression signatures of PC3. 66
Supplementary Fig. S32: Distribution of original and reconstructed gene overexpression signatures of VCAP. . 67
Supplementary Fig. S33: Protein expressions of activatory targets predicted for Huntington's diseases (HD). ... 68
Supplementary Fig. S34: Biological interpretation of protein knockdown VQ signatures. 69
Supplementary Fig. S35: Biological interpretation of protein overexpression VQ signatures. 70
Supplementary Fig. S36: Performance evaluation of target repositioning for each disease. 71
Supplementary Fig. S37: Performance evaluation of target repositioning for each protein. (A) Comparison of proposed (SSL-VQ, SSL-VAE, SL-VQ and SL-VAE) and baseline (SNP-PV, SNP-LD, SNP-eQTL and Multitask) methods for predicting inhibitory targets involving 33 diseases and 225 proteins. Boxplots represent AUC score distributions for proteins. (B) As described in (A), but for activatory target predictions involving 16 diseases and 37 proteins. 72

Supplementary Tables 73

Supplementary Table S1: Cell line list of target gene perturbation profiles. 73
Supplementary Table S2: Knockdown and overexpressed gene list. 74
Supplementary Table S3: Cell line list with missing rates below 90%. 83
Supplementary Table S4: All diseases of disease/patient-specific transcriptome profiles. 84
Supplementary Table S5: Diseases used in gold standard dataset. 86
Supplementary Table S6: Diseases used in gold standard dataset. 87
Supplementary Table S7: Diseases used in uncharacterized disease dataset. 88
Supplementary Table S8: Diseases used in uncharacterized disease dataset. 89
Supplementary Table S9: Proteins used in uncharacterized protein dataset. 90
Supplementary Table S10: Performance evaluation of predicting inhibitory indications for uncharacterized proteins. 91
Supplementary Table S11: Performance evaluation of predicting activatory indications for uncharacterized proteins. 93
Supplementary Table S12: Newly predicted inhibitory targets for uncharacterized diseases. 94
Supplementary Table S13: Newly predicted activatory targets for uncharacterized diseases. 97
Supplementary Table S14: The number of unlabeled and labeled samples used to assess of the influence of amount of unlabeled samples. 100
Supplementary Table S15: Training times of SSL-VQ for each parameter set. 101

Supplementary Methods

1. Protein signatures with multimodal VQ-VAE (protein multimodal VQ signatures)

Only cells with missing rates below 90% were used for model training. Specifically, 11 and 10 cells were utilized for gene knockdown and gene overexpression profiles, respectively (Supplementary Table S3). The input layer consisted of $C \times 978$ units (knockdown, $C = 11$; overexpression, $C = 10$), and the hidden layers consisted of $C \times 512$, 1024, and 512 units. The latent embedding size was set as $K = 256$. To prevent overfitting, dropout was applied to all encoder layers as well as the first and second hidden layers of the decoder, with a dropout rate of 0.1. The learning rate, number of epochs, and batch size were set as 2e-4, 500, and 64, respectively. For the activation function, we adopted the leaky rectified linear unit (LeakyReLU) with a negative slope of 0.5. Preprocessing for input profiles was achieved using MaxAbsScaling.

2. Proteins signatures with cell-specific VQ-VAE (protein cell-specific VQ signatures)

To compare with multimodal VQ-VAE, we modeled the protein perturbation process for each cell using VQ-VAE and extracted cell-specific features from protein perturbation profiles. We constructed protein signatures by concatenating cell-specific features across all cells.

We construct the encoder and decoder networks, composed of fully connected layers. The encoder's input layer consisted of $a\ (= 978)$ units, and the hidden layers consisted of 512, 256 and 128 units. Construction of the decoder mirrors that of the encoder. The model was trained for each cell, extracting the c-th cell's latent variables $\boldsymbol{z}_{\boldsymbol{e}}^{(c)}(\boldsymbol{x})$, and these variables were concatenated across all cells to obtain protein signatures $[\boldsymbol{z}_{\boldsymbol{e}}^{(1)}(\boldsymbol{x}), \boldsymbol{z}_{\boldsymbol{e}}^{(2)}(\boldsymbol{x}), \cdots, \boldsymbol{z}_{\boldsymbol{e}}^{(c)}(\boldsymbol{x})]^{\mathrm{T}}$ $(c = 1,2,\cdots,C)$. This signature is referred to as the "protein cell-specific VQ signatures."

3. Protein signatures with averaged VQ-VAE (protein averaged VQ signatures)

To compare with multimodal VQ-VAE, we employed VQ-VAE based on averaged protein perturbation profiles. To extract important features from protein perturbation profiles across various cells, the perturbation process was modeled by VQ-VAE from averaged protein perturbation profiles.

We constructed the averaged protein perturbation profiles of all cells for each s-th protein, represented by a feature vector $\boldsymbol{x}^{\mathrm{Ave}} = \left(x_1^{\mathrm{Ave}}, x_2^{\mathrm{Ave}}, \ldots, x_a^{\mathrm{Ave}}\right)^{\mathrm{T}}$ $(s = 1, 2, \cdots, S)$. Subsequently, a VQ-VAE model, as well as cell-specific VQ-VAE, was built and trained specifically for averaged protein perturbation profiles. In this context, we adopted the scaled exponential linear unit as activation function. Following model training, latent variables $\boldsymbol{z}_e(\boldsymbol{x}) = (z_1, z_2, \ldots, z_H)^{\mathrm{T}}$ were extracted, referred to as the "protein averaged VQ signature."

4. Protein signatures with averaged VAE (protein averaged VAE signatures)

To compare with VQ-VAEs, we employed ordinary VAE with continues latent variables based on averaged protein perturbation profiles. To extract important features from protein perturbation profiles across various cells, the perturbation process was modeled by VAE from averaged protein perturbation profiles.

We constructed the averaged protein perturbation profiles of all cells for each s-th protein, represented by a feature vector $\boldsymbol{x}^{\mathrm{Ave}} = \left(x_1^{\mathrm{Ave}}, x_2^{\mathrm{Ave}}, \ldots, x_a^{\mathrm{Ave}}\right)^{\mathrm{T}}$ $(s = 1, 2, \cdots, S)$. Subsequently, a VAE model, as well as VQ-VAEs, was built and trained specifically for averaged protein perturbation profiles. In this context, we adopted the hyperbolic tangent function as activation function. Following model training, latent variables $\boldsymbol{z}_e(\boldsymbol{x}) = (z_1, z_2, \ldots, z_H)^{\mathrm{T}}$ were extracted, referred to as the "protein averaged VAE signature."

5. Disease/patient-specific signatures with VQ-VAE (disease/patient VQ signatures)

To extract essential features from disease-specific profiles, we modeled disease states using VQ-VAE. Even if different diseases share similar pathological phenotypes, the molecular mechanisms do not change continuously from one disease to another. Therefore, we hypothesized that disease-specific transcriptome patterns would follow a discrete distribution, leading to the adoption of VQ-VAE with discrete latent variables. Additionally, given the limited number and high heterogeneity of disease-specific profiles, we enhanced model robustness by incorporating patient-specific profiles into the training process to augment disease-specific profiles.

Given D diseases, we explore how to model important features of disease-specific and patient-specific transcriptome patterns. Each d-th disease is represented as $\boldsymbol{v}_d^{\mathrm{Dis}} = \left(v_1^{\mathrm{Dis}}, v_2^{\mathrm{Dis}}, \dots, v_b^{\mathrm{Dis}}\right)^{\mathrm{T}}$ $(d = 1, 2, \cdots, D)$, and the d'-th patient with disease is represented as $\boldsymbol{v}_{d'}^{\mathrm{Pat}} = \left(v_1^{\mathrm{Pat}}, v_2^{\mathrm{Pat}}, \dots, v_b^{\mathrm{Pat}}\right)^{\mathrm{T}}$ $(d' = 1, 2, \dots, D')$, where D and D' represent the numbers of disease-specific and patient-specific profiles, respectively.

We construct encoder and decoder networks, consisting of fully connected layers. The input layer of encoder consists of b units to accommodate each disease/patient-specific profile (Fig. 1B). The decoder mirrors the encoder's structure.

Let the output of the encoder network be $\boldsymbol{z}_e(\boldsymbol{v})$; then, the posterior distribution of discrete latent variables is calculated as follows:

$$q(z = k|\boldsymbol{v}) = \begin{cases} 1 \text{ for } k = \underset{j}{\operatorname{argmin}} \left\| \boldsymbol{z}_e(\boldsymbol{v}) - \boldsymbol{e}_j \right\|_2 \\ 0 \text{ otherwise} \end{cases}. \tag{4}$$

The discrete latent variables $\boldsymbol{z}_e(\boldsymbol{v})$ are mapped to K types of latent embedding vectors in the latent embedding space, and the latent variables $\boldsymbol{z}_q(\boldsymbol{v})$ obtained through latent embedding are represented as follows:

$$\boldsymbol{z}_q(\boldsymbol{v}) = \boldsymbol{e}_k, \text{where } k = \underset{j}{\operatorname{argmin}} \left\| \boldsymbol{z}_e(\boldsymbol{v}) - \boldsymbol{e}_j \right\|_2. \quad (5)$$

The latent variable $\boldsymbol{z}_q(\boldsymbol{v})$ is used as input for the decoder. We jointly estimate all parameter sets of the encoder, decoder, and latent embedding space by minimizing the loss component as follows:

$$L = \log p\left(\boldsymbol{v} \middle| \boldsymbol{z}_q(\boldsymbol{v})\right) + \|\text{sg}[\boldsymbol{z}_e(\boldsymbol{v})] - \boldsymbol{e}\|_2^2 + \beta\|\boldsymbol{z}_e(\boldsymbol{v}) - \text{sg}[\boldsymbol{e}]\|_2^2, \quad (6)$$

where hyperparameter $\beta = 0.25$. After the model training, we extracted latent variables $\boldsymbol{z}_e(\boldsymbol{v}^{\text{Dis}}) = (z_1^{\text{Dis}}, z_2^{\text{Dis}}, \dots, z_H^{\text{Dis}})^{\text{T}}$ and $\boldsymbol{z}_e(\boldsymbol{v}^{\text{Pat}}) = (z_1^{\text{Pat}}, z_2^{\text{Pat}}, \dots, z_H^{\text{Pat}})^{\text{T}}$ for disease-specific and patient-specific profiles, respectively, referred to as "disease VQ signatures" and "patient VQ signatures", respectively.

In total, 295 patient-specific profiles and 79 disease-specific profiles were used for model training. Given the sparsity of disease-specific and patient-specific profiles, they were standardized during preprocessing, and genes with a variance of "0" were removed. The input layer consists of $b = 14{,}070$ units, and the hidden layers consist of 1000, 512, and 256 units. The latent embedding size was set as $K = 128$. To prevent overfitting, dropout was applied to all encoder layers and to the first and second hidden layers of the decoder, with a dropout rate of 0.1. The learning rate, number of epochs, and batch size were set as 2e-3, 2000, and 64, respectively. The hyperbolic tangent function was adopted as the activation function.

6. Disease/patient-specific signatures with VAE (disease/patient VAE signatures)

To compare with VQ-VAEs, we employed ordinary VAE with continues latent variables based on disease/patient-specific profiles. To extract important features from disease/patient-specific profiles, disease states were modeled by VAE from disease/patient-specific profiles.

Given D diseases, we explore how to model important features of disease-specific and patient-specific transcriptome patterns. Each d -th disease is represented as $\boldsymbol{v}_d^{\mathrm{Dis}} = \left(v_1^{\mathrm{Dis}}, v_2^{\mathrm{Dis}}, \dots, v_b^{\mathrm{Dis}}\right)^{\mathrm{T}}$ $(d = 1, 2, \cdots, D)$, and the d'-th patient with disease is represented as $\boldsymbol{v}_{d'}^{\mathrm{Pat}} = \left(v_1^{\mathrm{Pat}}, v_2^{\mathrm{Pat}}, \dots, v_b^{\mathrm{Pat}}\right)^{\mathrm{T}}$ $(d' = 1,2, \dots, D')$, where D and D' represent the numbers of disease-specific and patient-specific profiles, respectively. A VAE model, as well as VQ-VAEs, was built and trained specifically for disease/patient-specific profiles. In this context, we adopted the Rectified Linear Unit as activation function. Following model training, latent variables $\boldsymbol{z}_e\left(\boldsymbol{v}^{\mathrm{Dis}}\right) = (z_1^{\mathrm{Dis}}, z_2^{\mathrm{Dis}}, \dots, z_H^{\mathrm{Dis}})^{\mathrm{T}}$ and $\boldsymbol{z}_e(\boldsymbol{v}^{\mathrm{Pat}}) = (z_1^{\mathrm{Pat}}, z_2^{\mathrm{Pat}}, \dots, z_H^{\mathrm{Pat}})^{\mathrm{T}}$ were extracted for disease-specific and patient-specific profiles, respectively, referred to as “disease VQ signatures” and “patient VQ signatures”, respectively.

7. Semi-supervised learning-based neural network with VQ-VAE signatures (SSL-VQ)

A grid search was performed to determine the optimal hidden layer sizes and dropout rate. We predefined a set of candidate values {1024/512/256, 512/256/128, 256/128/64, 128/64/32} for hidden layer sizes and {0.1, 0.2, 0.3, 0.4, 0.5} for the dropout rate. Additionally, we set the learning rate to 0.0001, the maximum number of epochs to 2000, and the batch size to 64.

Regarding training times, as you suggested, we measured training times of SSL-VQ for each hyperparameter set (e.g., latent layer sizes and drop rates) and therapeutic target type (e.g., inhibitory targets or activatory targets). Supplementary Table S15 shows the training times taken for each hyperparameter set and therapeutic target type. For computational resources, iMac [Processor, 3.6 GHz, 10 core Inter Core i9; Computer memory, 128 GB 2267 MHz DDR4; OS, Mac OS (Sonoma 14.4.1)] was used for training the SSL-VQ model.

8. Protein perturbation transcriptome profiles

Protein perturbation profiles arising from gene knockdown or gene overexpression experiments were obtained from the L1000 database (Subramanian *et al.*, 2017). This database provided 978 landmark genes. Using "level 5" data, comprising profiles generated by collapsing several replicates, we incorporated 36,720 gene knockdown profiles (denoted "trt_sh.cgs") and 34,171 gene overexpression profiles (denoted "trt_oe"). Gene knockdown profiles were individualized by averaging biological replicates. In total, we constructed 4,345 gene knockdown profiles across 17 cells and 4,040 gene overexpression profiles across 21 cells (refer to Supplementary Tables S1 and S2).

Protein perturbation profiles served as feature vectors for candidate target proteins. Transcriptome profiles, constructed from gene knockdown and gene overexpression, were referred to as "gene knockdown profiles" and "gene overexpression profiles," respectively, collectively referred to as "protein perturbation profiles." Each of gene knockdown and overexpression profiles was represented as a feature vector, $\boldsymbol{x}^{\mathrm{inh}} = \left(x_1^{\mathrm{inh}}, x_2^{\mathrm{inh}}, \ldots, x_a^{\mathrm{inf}}\right)^{\mathrm{T}}$ and $\boldsymbol{x}^{\mathrm{act}} = (x_1^{\mathrm{act}}, x_2^{\mathrm{act}}, \ldots, x_a^{\mathrm{act}})^{\mathrm{T}}$, respectively, where a =978 is the number of genes. Due to numerous missing entries in protein perturbation profiles, we imputed those values using a tensor decomposition algorithm (Iwata *et al.*, 2019).

9. Data completion method

We imputed the missing entries of the protein perturbation profiles using a tensor decomposition algorithm (Iwata *et al.*, 2019). Protein perturbation data can be represented by a third-order tensor. The gene knockdown data consisted of 4,345 knocked down genes, 978 genes, and 17 cell lines, and can be represented as a $4{,}345 \times 978 \times 17$ tensor. Similarly, gene overexpression data consisted of 4,040 overexpressed genes, 978 genes, and 20 cell lines, and was represented as a $4{,}040 \times 978 \times 20$ tensor. Most parts of these tensors are missing or unobserved.

10. Disease/patient-specific transcriptome profiles

Transcriptome profiles of patients with various diseases were obtained from the CREEDs database (Wang *et al.*, 2016) using the characteristic direction method (Clark *et al.*, 2014), comparing gene expression measurements in diseased and control tissues.

We extracted profiles from humans for 79 diseases and 14,804 genes, referring to the gene expression profiles of patients as “patient-specific profiles.” These were represented by a feature vector, $\boldsymbol{v}^{\text{Pat}} = \left(v_1^{\text{Pat}}, v_2^{\text{Pat}}, \dots, v_b^{\text{Pat}}\right)^{\text{T}}$, where b is the number of genes. Finally, multiple patient-specific profiles for the same disease were averaged, yielding a disease-specific profiles for each of the 79 diseases. Transcriptome profile of each disease was represented as $\boldsymbol{v}^{\text{Dis}} = \left(v_1^{\text{Dis}}, v_2^{\text{Dis}}, \dots, v_b^{\text{Dis}}\right)^{\text{T}}$.

11. Validation of predicted therapeutic targets for Huntington's disease (HD)

We validated predicted activatory targets for HD using an independent resource, HD cohort. The cohort data consisted of three groups, i.e., control (n =24), before disease onset (pre-HD; $n = 23$), and patients with early HD (early-HD; $n = 21$), as well as three tissues, namely adipose, muscle, and fibroblasts (Neueder *et al.*, 2022). Gene-expression data was downloaded from the original paper.

We compared the levels of gene expression for predicted activatory targets among control, pre-HD and early-HD. Where the gene-expression levels of an activatory target were lower in pre-HD or early-HD than control, an association was suggested between the target and HD.

Supplementary Results

1. Performance evaluation of new indication predictions for uncharacterized proteins

We evaluated the prediction accuracy of SSL-VQ in predicting therapeutic indication for uncharacterized proteins. Models were trained with gold standard data, where proteins had at least one known therapeutic association with a disease. The models were subsequently evaluated through their application to uncharacterized proteins without known therapeutic associations with diseases (refer to the "new indication prediction for uncharacterized proteins" scenario in the Materials and Methods). SSL-VQ was compared with SL-VQ, SNP-PV, SNP-eQTL, and Multitask.

Supplementary Figure S3 illustrates the results of the performance evaluation in predicting therapeutic indications for uncharacterized proteins. Regarding inhibitory target predictions (Supplementary Fig. S3A), SSL-VQ demonstrated higher accuracy compared with the baseline methods. SSL-VQ significantly outperformed the baseline methods, particularly for HPRT1 (AUC=0.83) and TLR9 (AUC=0.78) (Supplementary Table S10). Regarding activatory target predictions (Supplementary Fig. S3B), SSL-VQ exhibited superior prediction performance compared with the baseline methods, demonstrating significantly improved prediction accuracy, especially for HTR4 (AUC=0.71) and ADORA2A (AUC=0.68) (Supplementary Table S11). Additionally, SL-VQ outperformed the baseline methods in relation to HTR4 (AUC=0.90) and GABBR1 (AUC=0.74). These results suggest that SSL-VQ effectively predicts therapeutic indications for uncharacterized proteins by incorporating fused features of protein perturbation patterns in multiple cells. The dependency of prediction accuracy on protein classes was also evaluated.

The dependency of prediction accuracy on protein classes was also evaluated. For inhibitory targets, SSL-VQ outperformed the baseline methods for transmembrane signal receptors and metabolite enzymes (Supplementary Fig. S6A). SL-VQ exhibited a similar outperformance,

particularly for DNA metabolism proteins and protein modifying enzymes. Regarding activatory targets, SSL-VQ outperformed the baseline methods for metabolite interconversion enzymes and translational regulators, and SL-VQ outperformed the baseline methods for transmembrane signal receptors (Supplementary Fig. S6B). These findings indicate that SSL-VQ can predict new applicable diseases for various uncharacterized proteins by considering fused features of protein perturbation patterns across various cells.

2. Feature extraction processes from multiple cell types

We explored the impact of feature extraction processes from multiple cell types on prediction accuracy. Six patterns, representing combinations of models and protein VQ signatures, were compared: SSL with protein multimodal VQ signatures, SSL with protein cell-specific VQ signatures, SSL with protein averaged VQ signatures, SL with protein multimodal VQ signatures, SL with protein cell-specific VQ signatures, and SL with protein averaged VQ signatures.

Supplementary Figure S7 show the results of performance evaluation for target-disease pairs. Models were evaluated in the "target repositioning for target-disease pairs" scenario. All protein VQ signatures showed highly similar prediction performances for both inhibitory and activatory target predictions (Supplementary Fig. S7A and S7B). Thus, SSL with any protein VQ signatures is considered effective approaches in the target repositioning framework.

We also examined the influence of feature extraction processes from multiple cells on prediction accuracy in the "new target prediction for uncharacterized diseases" scenario. Regarding inhibitory target prediction, SSL with protein multimodal VQ signatures and SL with protein multimodal VQ signatures exhibited superior prediction performance (Supplementary Fig. S7C). Concerning activatory target prediction, there were no major differences between the types of protein VQ signatures, although SL with protein averaged VQ signatures, SSL with protein averaged VQ signatures, and SSL with protein multimodal VQ signatures exhibited slightly better performance compared with the other signatures (Supplementary Fig. S7D). These findings suggest that SSL with protein multimodal VQ signatures or protein averaged VQ signatures are useful approaches in the new target prediction for uncharacterized diseases.

Additionally, we explored the impact of feature extraction processes from multiple cells on prediction accuracy in the "new indication prediction for uncharacterized proteins" scenario. Concerning inhibitory target prediction, SSL with protein multimodal VQ signatures demonstrated

slightly better prediction performance compared with the other protein VQ signatures (Supplementary Fig. S7E). For activatory target prediction, SSL with protein multimodal VQ signatures and SSL with protein averaged VQ signatures exhibited the best performance (Supplementary Fig. S7F). Collectively, these results highlight SSL with protein multimodal VQ signatures and protein averaged VQ signatures as valuable approaches in the new indication prediction for uncharacterized proteins.

3. Performance comparison between VQ-VAE and VAE signatures

We explored the impact of feature extraction processes using VQ-VAE with discrete latent variables and VAE with continuous latent variables on prediction accuracy. Four patterns, representing combinations of models and signatures, were compared: SSL with VQ-VAE signatures, SSL with VAE signatures, SL with VQ-VAE signatures, and SL with VAE signatures. VQ-VAE signatures represent the case of using disease VQ signatures constructed by VQ-VAE and protein averaged VQ signatures constructed by VQ-VAE, while VAE signatures represent the case of using disease VAE signatures constructed by VAE and protein averaged VAE signatures constructed by VAE.

Supplementary Figures S8A and S8B show the results of performance evaluation for inhibitory target-disease pairs and activatory target-disease pairs, respectively. Models were evaluated in the "target repositioning for target-disease pairs" scenario. SSL with VQ-VAE signatures exhibited the best prediction performance for activatory target prediction. Regarding inhibitory target prediction, VAE signatures were more accurate than VQ-VAE signatures. For activatory target prediction, VQ-VAE signatures exhibited better accuracy than VAE signatures in both SL and SSL frameworks. These results suggest that VQ-VAE signatures, constructed using VQ-VAE with discrete latent variables, are useful in the target repositioning framework.

We also examined the influence of feature extraction processes using VQ-VAE and VAE on prediction accuracy in the "new target prediction for uncharacterized diseases" scenario. SSL with VQ-VAE signatures exhibited the best prediction performance for both inhibitory and activatory target predictions (Supplementary Fig. S8C and S8D). VQ-VAE signatures were clearly more accurate for both SSL and SL frameworks. These results suggest that VQ-VAE signatures, constructed using VQ-VAE with discrete latent variables, are useful in the new target prediction for uncharacterized diseases.

Additionally, we explored the impact of feature extraction processes using VQ-VAE and VAE on prediction accuracy in the "new indication prediction for uncharacterized proteins" scenario. SSL

with VQ-VAE signatures exhibited the best prediction performance for both inhibitory and activatory target predictions (Supplementary Fig. S8E and S8F). Especially for activatory target prediction, using VQ-VAE signatures significantly improved the prediction accuracy of VAE signatures. These results suggest that VQ-VAE signatures, constructed using VQ-VAE with discrete latent variables, are useful in the new indication prediction for uncharacterized proteins.

4. Codebook collapse in VQ-VAE

We investigated the impact of activation functions on modeling of protein perturbation profiles using multimodal VQ-VAE. VQ-VAE is known to suffer from codebook collapse when the original feature vectors are numerically similar. We examined the occurrence of codebook collapse when using SELU and LeakyReLU as activation functions. Regarding gene knockdown signatures, codebook collapse did not occur in both SELU and LeakyReLU. Regarding gene overexpression signatures, codebook collapse occurred in SELU, whereas LeakyReLU did not cause codebook collapse.

Supplementary Figures S11–22 and S23–S32 shows distribution of original and reconstructed gene knockdown signatures, and that of original and reconstructed gene overexpression signatures, respectively. For gene knockdown signatures, there was almost no difference in reconstruction errors between SELU and LeakyReLU, whereas in gene overexpression signatures, reconstruction errors were smaller with LeakyReLU than with SELU for many proteins in many cells. The reconstruction errors were significantly improved especially for A375, A549, HA1E, and VCAP. These results indicate that codebook collapse in overexpresion signatures can be overcome by using LeakyReLU as an activation function.

5. Biological interpretation of newly predicted therapeutic targets for Huntington’s diseases

We further validated activatory targets predicted for Huntington's disease, a rare disease. Protein expression levels of the candidate activatory targets [ACTN1, AK1, and RHOBTB1 (shown in Fig. 4C–E)] were examined across 44 normal tissues using The Human Protein Atlas [https://www.proteinatlas.org/] (Supplementary Fig. S33). Note that ARHGEF (Fig. 4F) was not examined due to lack of information from The Human Protein Atlas. The protein expression levels were assessed at four levels—“Not detected,” “Low,” “Medium,” and “High”—according to the criteria defined by The Human Protein Atlas.

Supplementary Figure S33 shows protein expressions of the candidate activatory targets predicted for Huntington’s diseases. ACTN1 was expressed in only a few tissues and was expressed at the “Medium” levels in the cerebral cortex and caudate (Supplementary Fig. S33A). Caudate is located in the basal ganglia, and Huntington's disease is a neurodegenerative disorder where neurons in the basal ganglia gradually decrease, indicating that ACTN1 may be a potential activatory target for Huntington’s disease. AK1 was expressed at the “Low” levels in the cerebral cortex, hippocampus, and caudate (Supplementary Fig. S33B). THOBTB1 was expressed at the “High” levels in the cerebellum, hippocampus, and caudate (Supplementary Fig. S33C). Collectively, these results affirm the validity of the predicted activatory targets for Huntington's disease.

6. Performance evaluation of SSL-VQ based on the F1 measure

We assessed the models' performance based on not only AUC but also the F1 measure (also known as the F-score), which is a harmonic mean of precision and recall. The F1 measure ranges from 0 to 1.0, with 1.0 indicating perfect inference (100% precision, 100% recall). In this evaluation, the threshold that yielded the maximum F1 measure was selected. A performance comparison was conducted between proposed (SSL-VQ, SSL-VAE, SL-VQ and SL-VAE) and baseline (SNP-PV, SNP-LD, SNP-eQTL, and Multitask) methods.

First, we evaluated the performance of proposed methods in target repositioning, a scenario involving the repositioning of existing therapeutic targets to other diseases. Supplementary Fig. S1A and S1B show the results of performance evaluations based on the F1 measure for inhibitory and activatory target predictions, respectively. In the inhibitory target prediction, the proposed methods tended to work better than the baseline methods. This trend was consistent with inhibitory and activatory target predictions. These results suggest that the proposed methods excel in predicting inhibitory and activatory targets separately with stable accuracy, outperforming the baseline methods by considering fused patterns of protein perturbations across various cell types.

Second, we evaluated the performance of SSL-VQ in predicting therapeutic targets for uncharacterized diseases. Supplementary Fig. S2A and S2B shows the results of performance evaluations based on the F1 measure for inhibitory and activatory target predictions, respectively. In the inhibitory target prediction, SSL-VQ had the best accuracy, followed by the SL-VAE and SNP-PV. For activatory target prediction, the SSL-VQ tended to work better than the baseline methods. These findings suggest that SSL-VQ can effectively predict therapeutic targets for uncharacterized diseases without known therapeutic targets.

Third, we evaluated the prediction accuracy of SSL-VQ in predicting therapeutic indication for uncharacterized proteins. Supplementary Fig. S4A and S4B show the results of performance

evaluations based on the F1 measure for inhibitory and activatory target predictions, respectively. SSL-VQ, SL-VQ, and Multitask worked well. This trend was consistent with inhibitory and activatory target predictions. These results suggest that SSL-VQ can predict new applicable diseases for various uncharacterized proteins.

7. Performance comparisons when trained on datasets with different amounts of unlabeled samples

We assessed the impact of the amount of unlabeled data on prediction accuracy of SSL-VQ. We trained the SSL-VQ model on three datasets with varying amounts of unlabeled data: (I) the 'Small' dataset, where all pairs of known targets and uncharacterized diseases were defined as unlabeled samples; (II) the 'Middle' dataset, where all pairs of known diseases and uncharacterized proteins were defined as unlabeled samples; and (III) the 'Large' dataset, where all pairs of known targets and uncharacterized diseases and all pairs of known diseases and uncharacterized proteins were defined as unlabeled samples. The number and ratios of unlabeled and labeled samples in the three datasets are shown in Supplementary Table S14.

We evaluated the performances of SSL-VQ in target repositioning, a scenario involving the repositioning of existing therapeutic targets to other diseases (Supplementary Fig. S9). There was little difference in accuracy among the three datasets for both inhibitory and activatory target predictions (Supplementary Fig. S9A and S9B). These results suggest that increasing the information on uncharacterized diseases or proteins from unlabeled samples do not improve prediction accuracy as the aim of target repositioning is to detect missing associations between known therapeutic target proteins and diseases.

We evaluated the performance of SSL-VQ in predicting therapeutic targets for uncharacterized diseases (Supplementary Fig. S9). For inhibitory target prediction, the SSL-VQ trained with the "Large" dataset had the best accuracy (Supplementary Fig. S9C). The SSL-VQ trained with the "Small" dataset was slightly more accurate than that with the "Middle" dataset. This is because the "Small" and "Large" dataset include information on uncharacterized diseases in the unlabeled samples, whereas the "Middle" dataset does not include information on uncharacterized diseases. For activatory target prediction, there was little difference in prediction accuracy among the

three datasets (Supplementary Fig. S9D). These results suggest that increasing unlabeled samples for uncharacterized diseases could improve the accuracy of predicting therapeutic targets for uncharacterized diseases.

We evaluated the prediction accuracy of SSL-VQ in predicting therapeutic indication for uncharacterized proteins (Supplementary Fig. S9). For inhibitory target prediction, the SSL-VQ trained with the “Middle” dataset was slightly more accurate than those with the other datasets (Supplementary Fig. S9E). For activatory target prediction, there was little difference among the three datasets (Supplementary Fig. S9F). These results suggest that unlabeled samples including uncharacterized proteins can improve the accuracy for predicting therapeutic indication for uncharacterized proteins.

8. Performance evaluation of the proposed methods for common diseases and rare diseases

We stratified the performances of the proposed methods by disease category (rare vs. common diseases). Here, diseases from the Orphanet database [https://www.orpha.net/] are treated as rare diseases, while other diseases are treated as common diseases.

We evaluated the performances of SSL-VQ and SSL-VAE in target repositioning, a scenario involving the repositioning of existing therapeutic targets to other diseases (Supplementary Fig. S10). For inhibitory target prediction, SSL-VQ performed well for many rare diseases (Supplementary Fig. S10A), indicating that learning information on uncharacterized diseases from unlabeled samples enhances the applicability for rare diseases. For activatory target prediction, the accuracy for rare diseases was lower than that for common diseases (Supplementary Fig. S10B). This inconsistent trend between inhibitory and activatory target predictions may be due to more limited information on known activatory targets.

We evaluated the performance of SSL-VQ in predicting therapeutic targets for uncharacterized diseases (Supplementary Fig. S10). For inhibitory target prediction, SSL-VQ had the highest accuracy and the accuracy for rare diseases was higher than that for common diseases (Supplementary Fig. S10C). For activatory target prediction, SSL-VQ had the highest accuracy, and there were no significant differences between common and rare diseases (Supplementary Fig. S10D). These results suggest that SSL-VQ can be applicable to a range of diseases including rare diseases.

9. Model interpretability and biological relevance of multimodal VQ-VAE

We examined biological meaning of the features learned by VQ-VAE. We assessed the contribution of input genes to each latent variable forming the protein multimodal VQ signatures using SHapley Additive exPlanations (SHAP) (Lundberg and Lee, 2017) (Supplementary Figs. S34 and S35). SHAP values reflect the impact on the model output.

For protein knockdown VQ signatures, low expressions of genes such as *S100A4* in HEPG2 cell line and *TIMP2* in HA1E cell line contributed to the dimension 1 (Supplementary Fig. S34A). Conversely, high expressions of *CDH3* in VCAP cell line and *RAB11FIP2* in HA1E cell line contributed to the dimension 1. The contributions to other dimensions were similarly examined (Supplementary Fig. S34B–F). For protein overexpression VQ signatures, low expressions of *ORC1* in PC3 cell line and *SYK* in HEPG2 cell line contributed to the dimension 1 (Supplementary Fig. S35A). Conversely, high expressions of *TCEA2* in PC3 cell line and *SCAND1* in MCF7 cell line contributed significantly to dimension 1. The contributions to other dimensions were similarly examined (Supplementary Fig. S35B–F). These results suggest that protein multimodal VQ signatures reflect information on a variety of genes of cell lines.

10. Performance evaluation of SSL-VQ for each disease and protein

We tested prediction accuracy for diseases and targets in the target repositioning scenario involving the repositioning of existing therapeutic targets to other diseases. A performance comparison was conducted between proposed (SSL-VQ, SSL-VAE, SL-VQ and SL-VAE) and baseline (SNP-PV, SNP-LD, SNP-eQTL, and Multitask) methods. Note that other scenarios have already been tested for prediction accuracy against diseases and targets (Fig. 3 and Supplementary Fig. S3).

For performance evaluation for each disease, the proposed methods worked better than the baseline methods (Supplementary Fig. S36), as observed in the performance evaluation for all target-disease pairs (Fig. 2). For inhibitory target prediction, SL-VQ was slightly inferior to SSL-VAE and SSL-VQ (Supplementary Fig. S36A). This trend was consistent with inhibitory and activatory target predictions. These results suggest that proposed methods excel in predicting inhibitory and activatory targets separately with stable accuracy, outperforming baseline methods by considering fused patterns of protein perturbations across various cell types.

For performance evaluation for each protein, the proposed methods worked better than the baseline methods (Supplementary Fig. S37). For activatory target prediction, SSL-VQ and Multitask had the highest prediction accuracy. These results suggest that proposed methods can outperform baseline methods by considering fused patterns of protein perturbations across various cell types.

Supplementary Discussion

The SNP profiling is a popular method to detect biomolecules that are related with a disease with strong genetic factors. The SNP profiling method's accuracy was unstable in the therapeutic target prediction task (Figs. 2 and 3). It also struggles to distinguish between inhibitory and activatory targets. In contrast, our proposed method can distinguish between inhibitory and activatory targets. This superiority may be attributed to its comprehensive consideration of protein perturbation profiles, reflecting the inhibitory or activating functions of target proteins. As more protein perturbation profiles are accumulated, our method's applicability range and accuracy could further improve. Integration of biomolecular network information to consider the *in vivo* environment (Han *et al.*, 2022) could also enhance prediction validity.

VQ-VAE facilitated representation learning, enabling the extraction of informative features from disease-specific and protein perturbation profiles among various cell types. VQ-VAE worked better than ordinary VAE, a generative model with continuous latent variables (see Supplementary Results in Supplementary Information), suggesting that VQ-VAE is suited for feature extraction of protein and disease transcriptome profiles. Furthermore, VQ-VAE-based dimension reduction of transcriptome profiles enabled to perform SSL with a huge amount of unlabeled pairs, which was not possible when using the original transcriptome profiles. However, VQ-VAE is known to suffer from codebook collapse when the original feature vectors are numerically similar. In this study, we addressed this issue by incorporating the LeakyReLU function into the neural network activation function. An alternative solution could involve using stochastically quantized VAE (Takida *et al.*, 2022).

Our proposed SSL framework–based method aligns with the increased attention directed toward SSL. In drug discovery research, SSL has been applied to predict chemical toxicity (Chen *et al.*, 2021), compound bioactivity (Watson *et al.*, 2021), and compound–protein interactions (Xia *et al.*, 2010).

When predicting therapeutic targets, the available information on known therapeutic target-disease associations is limited. SSL is a suitable approach, especially in cases involving diseases without established therapeutic targets or with minimal target information.

Rare diseases generally have limited available data. Even for rare diseases with limited available data, VQ-VAE models can be applied if gene expression profiles from disease patients are available. However, it is assumed that the disease-specific transcriptome profiles will be heterogeneous as it is constructed by averaging the limited number of patient-specific transcriptome profiles. In this study, to mitigate the heterogeneous nature of disease/patient-specific transcriptome profiles of rare diseases, SSL-VQ employs a pi-model that takes into account the consistency between disease-specific and patient-specific transcriptome profiles. This enables stable and accurate predictions for heterogeneous rare diseases.

In recent years, several key methodologies and models have been developed (Zeng, Zhu, *et al.*, 2020; Zhou *et al.*, 2020; Zeng *et al.*, 2019; Zeng, Song, *et al.*, 2020). The previous studies have focused on predicting new target candidates for known drugs and predicting new drug candidates for diseases through drug repositioning (Zeng, Zhu, *et al.*, 2020; Zhou *et al.*, 2020; Zeng *et al.*, 2019; Zeng, Song, *et al.*, 2020) [https://doi.org/10.1039/C9SC04336E; 10.1038/s41421-020-0153-3; 10.1093/bioinformatics/btz418; and 10.1021/acs.jproteome.0c00316], whereas the present study focused on predicting new therapeutic target candidates for diseases including uncharacterized diseases. The therapeutic targets in this study include both targets with and without known drugs. Thus, pharmaceutical companies can select any compound that interacts with the identified therapeutic targets. The objective of this study is different from those of the previous studies.

Supplementary References

Chen,J. *et al.* (2021) Chemical toxicity prediction based on semi-supervised learning and graph convolutional neural network. *J. Cheminform.*, **13**, 1–16.

Clark,N.R. *et al.* (2014) The characteristic direction: A geometrical approach to identify differentially expressed genes. *BMC Bioinformatics*, **15**.

Han,Y. *et al.* (2022) Empowering the discovery of novel target-disease associations via machine learning approaches in the open targets platform. *BMC Bioinformatics*, **23**, 1–19.

Iwata,M. *et al.* (2019) Predicting drug-induced transcriptome responses of a wide range of human cell lines by a novel tensor-train decomposition algorithm. *Bioinformatics*, **35**, i191–i199.

Lundberg,S.M. and Lee,S.I. (2017) A unified approach to interpreting model predictions. In, *Advances in Neural Information Processing Systems*., pp. 4766–4775.

Neueder,A. *et al.* (2022) Abnormal molecular signatures of inflammation, energy metabolism, and vesicle biology in human Huntington disease peripheral tissues. *Genome Biol.*, **23**, 1–21.

Subramanian,A. *et al.* (2017) A Next Generation Connectivity Map: L1000 platform and the first 1,000,000 profiles. *Cell*, **171**, 1437-1452.e17.

Takida,Y. *et al.* (2022) SQ-VAE: Variational Bayes on Discrete Representation with Self-annealed Stochastic Quantization. *Proc. Mach. Learn. Res.*, **162**, 20987–21012.

Wang,Z. *et al.* (2016) Extraction and analysis of signatures from the Gene Expression Omnibus by the crowd. *Nat. Commun.*, **7**.

Watson,O. *et al.* (2021) A semi-supervised learning framework for quantitative structure–activity regression modelling. *Bioinformatics*, **37**, 342–350.

Xia,Z. *et al.* (2010) Semi-supervised drug-protein interaction prediction from heterogeneous biological spaces. *BMC Syst. Biol. 2010 42*, **4**, 1–16.

Zeng,X. *et al.* (2019) deepDR: a network-based deep learning approach to in silico drug repositioning. *Bioinformatics*, **35**, 5191–5198.

Zeng,X., Song,X., *et al.* (2020) Repurpose Open Data to Discover Therapeutics for COVID-19 Using Deep Learning. *J. Proteome Res.*, **19**, 4624–4636.

Zeng,X., Zhu,S., *et al.* (2020) Target identification among known drugs by deep learning from heterogeneous networks. *Chem. Sci.*, **11**, 1775–1797.

Zhou,Y. *et al.* (2020) Network-based drug repurposing for novel coronavirus 2019-nCoV/SARS-CoV-2. *Cell Discov. 2020 61*, **6**, 1–18.

Supplementary Figures

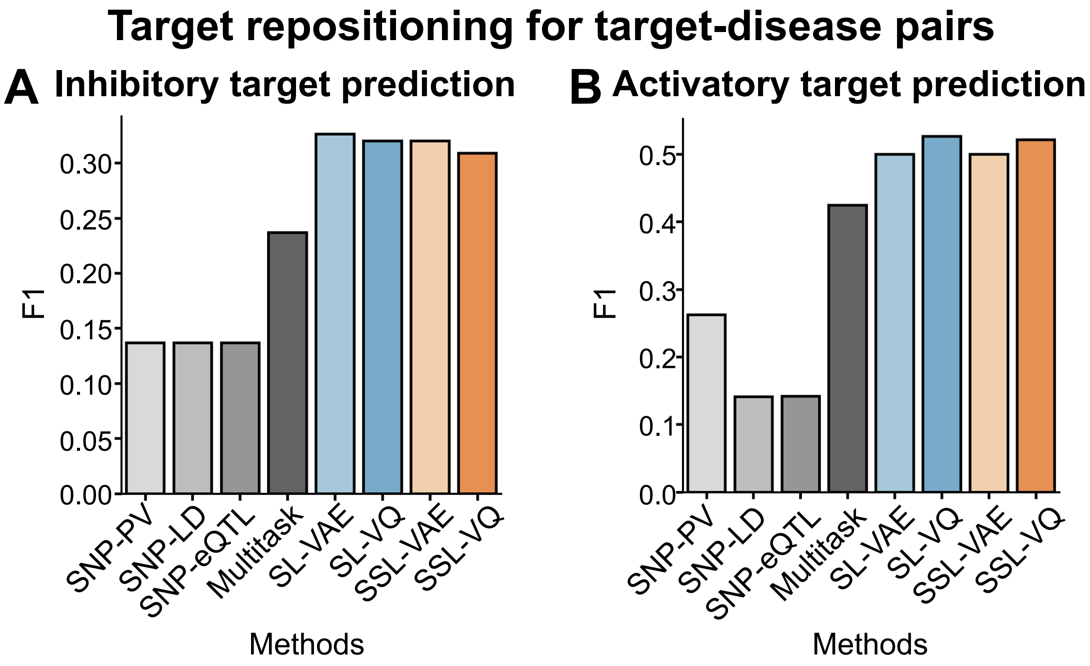

Supplementary Fig. S1: Performance evaluation of target repositioning for target-disease pairs based on the F1 measure.

(A) Comparison of proposed (SSL-VQ, SSL-VAE, SL-VQ and SL-VAE) and baseline (SNP-PV, SNP-LD, SNP-eQTL and Multitask) methods for predicting inhibitory targets for 33 diseases and 225 proteins. (B) As described in (A), but for activatory target predictions involving 16 diseases and 37 proteins.

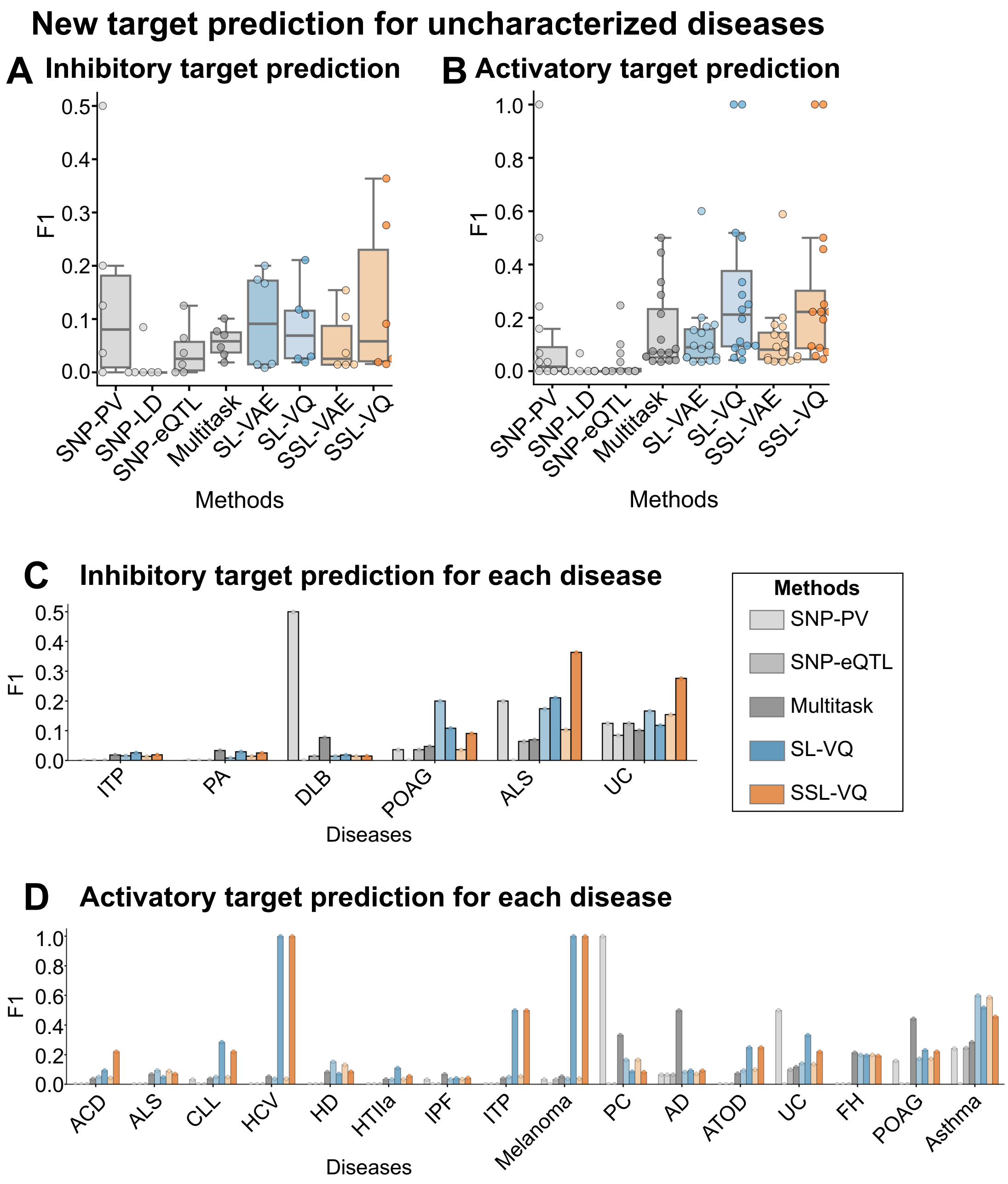

Supplementary Fig. S2: Performance evaluation of new target predictions for uncharacterized diseases based on the F1 measure.

(A) Comparison of proposed (SSL-VQ, SSL-VAE, SL-VQ and SL-VAE) and baseline (SNP-PV, SNP-LD, SNP-eQTL and Multitask) methods for predicting inhibitory targets involving 6 diseases and 30 proteins. Boxplots represent the F1 measures for diseases. (B) As described in (A), but for activatory target predictions involving 16 diseases and 26 proteins. (C) As described in (A), but showing bar graphs representing inhibitory target predictions for each disease. (D) As described in (C), but for activatory target predictions. Disease abbreviations: IPF, idiopathic pulmonary fibrosis; FH, familial hypercholesterolemia.

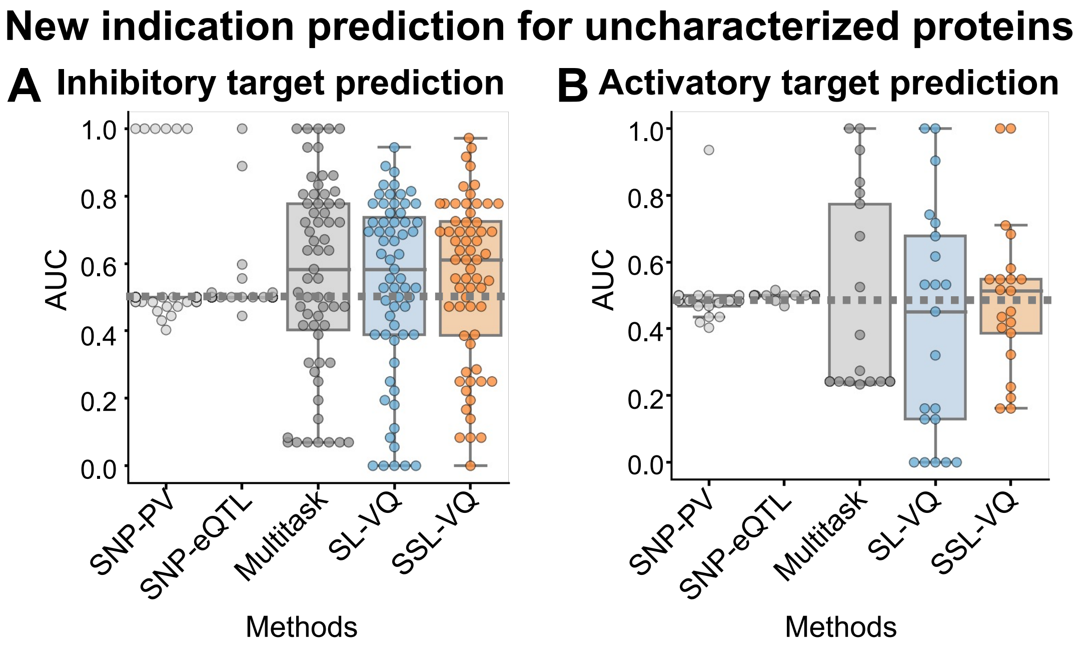

Supplementary Fig. S3: Performance evaluation of new indication prediction for uncharacterized proteins based on AUC.

Comparison of the performance of proposed and baseline methods for predicting inhibitory indications for 24 diseases and 63 proteins; the proposed methods are SSL-VQ and SL-VQ; the baseline methods are SNP-PV, SNP-eQTL, and Multitask. Models were trained and evaluated based on a "new indication prediction for uncharacterized proteins" scenario (Methods). The boxplots represent the distributions of AUC scores for each protein. (B) Same as (A) but for activatory indication predictions for 18 diseases and 21 proteins.

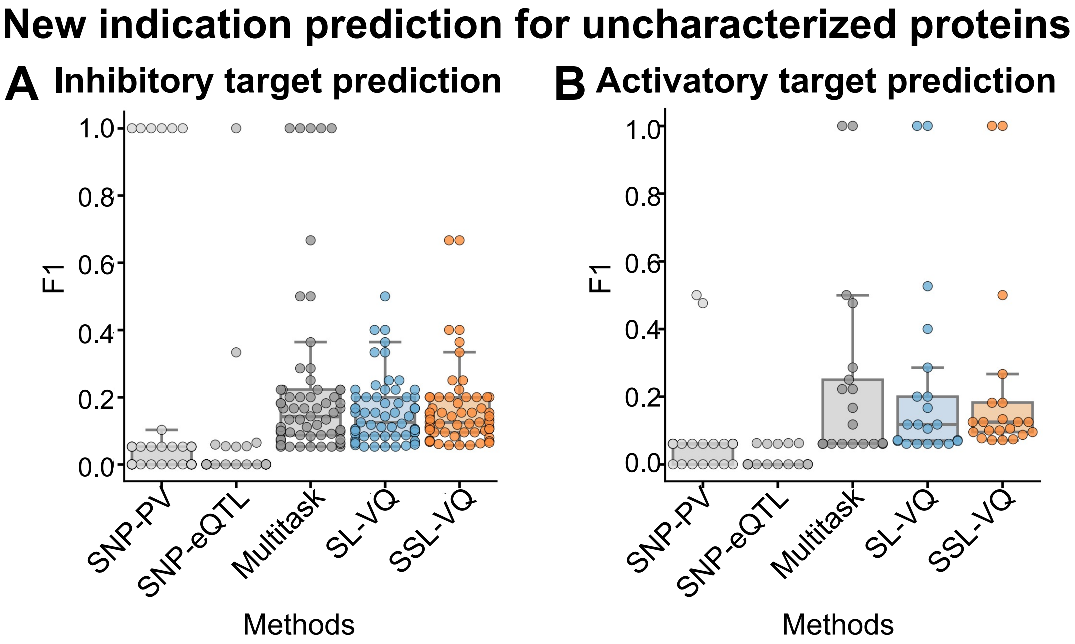

Supplementary Fig. S4: Performance evaluation of new indication prediction for uncharacterized proteins based on the F1 measure.

(A) Comparison of the performance of proposed and baseline methods for predicting inhibitory indications for 24 diseases and 63 proteins; the proposed methods are SSL-VQ and SL-VQ; the baseline methods are SNP-PV, SNP-eQTL, and Multitask. Models were trained and evaluated based on a "new indication prediction for uncharacterized proteins" scenario (Methods). The boxplots represent the distributions of the F1 measures for proteins. (B) Same as (A) but for activatory indication predictions for 18 diseases and 21 proteins.

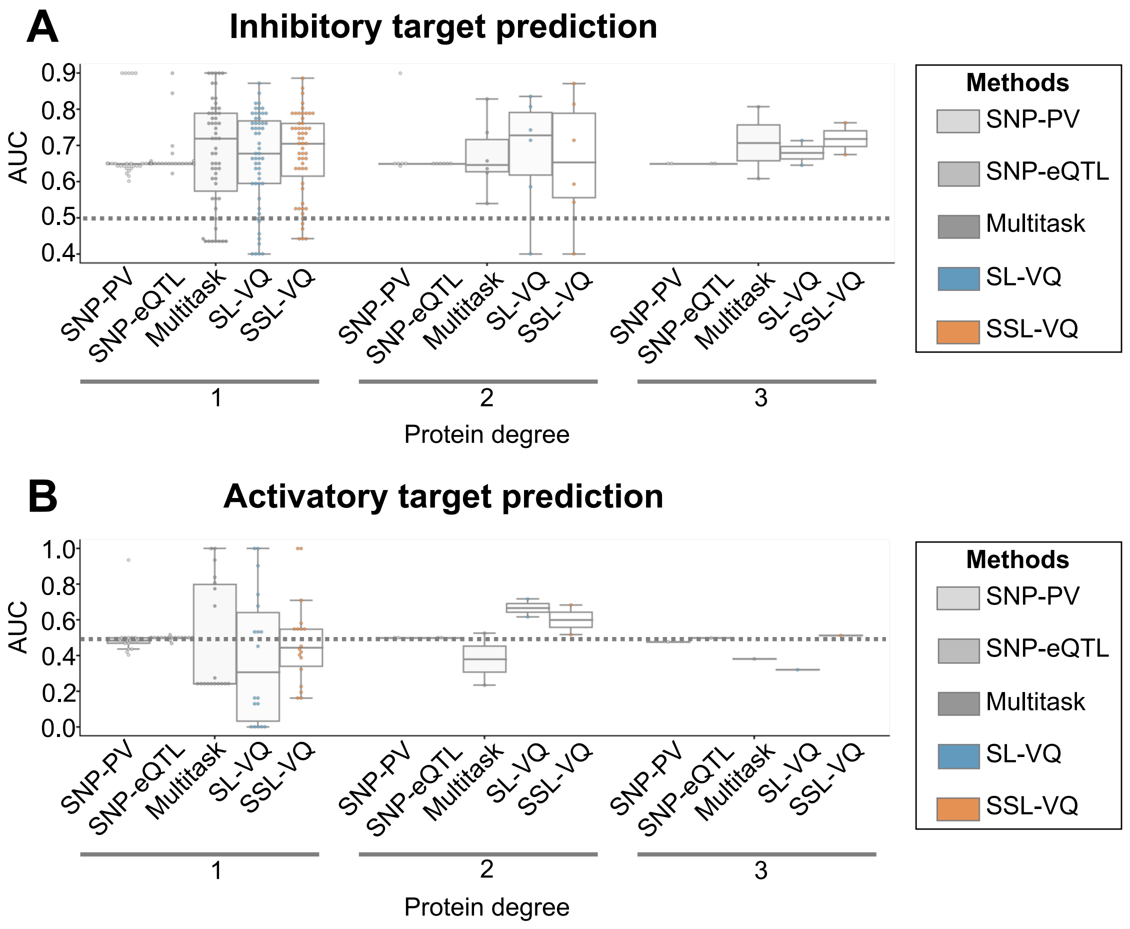

Supplementary Fig. S5: Performance evaluation of proposed methods for predicting therapeutic indications for uncharacterized proteins.

(A) Comparison of the performance of the proposed methods and baseline methods for predicting inhibitory targets for 24 diseases and 63 proteins; the proposed methods correspond to the SSL-VQ and SL-VQ. The baseline methods correspond to SNP-PV, SNP-eQTL and multitask learning method. Models were trained and evaluated based on a "uncharacterized protein" scenario (Methods). The boxplots represent the distributions of AUC scores for each target. In the box plots: center line, median; box, interquartile range; whiskers, 1.5× interquartile range; and point, AUC score for the target. Colors represent prediction methods. (B) Same as (A) but for activatory target predictions for 18 diseases and 21 proteins. The horizontal dotted line represents AUC = 0.5.

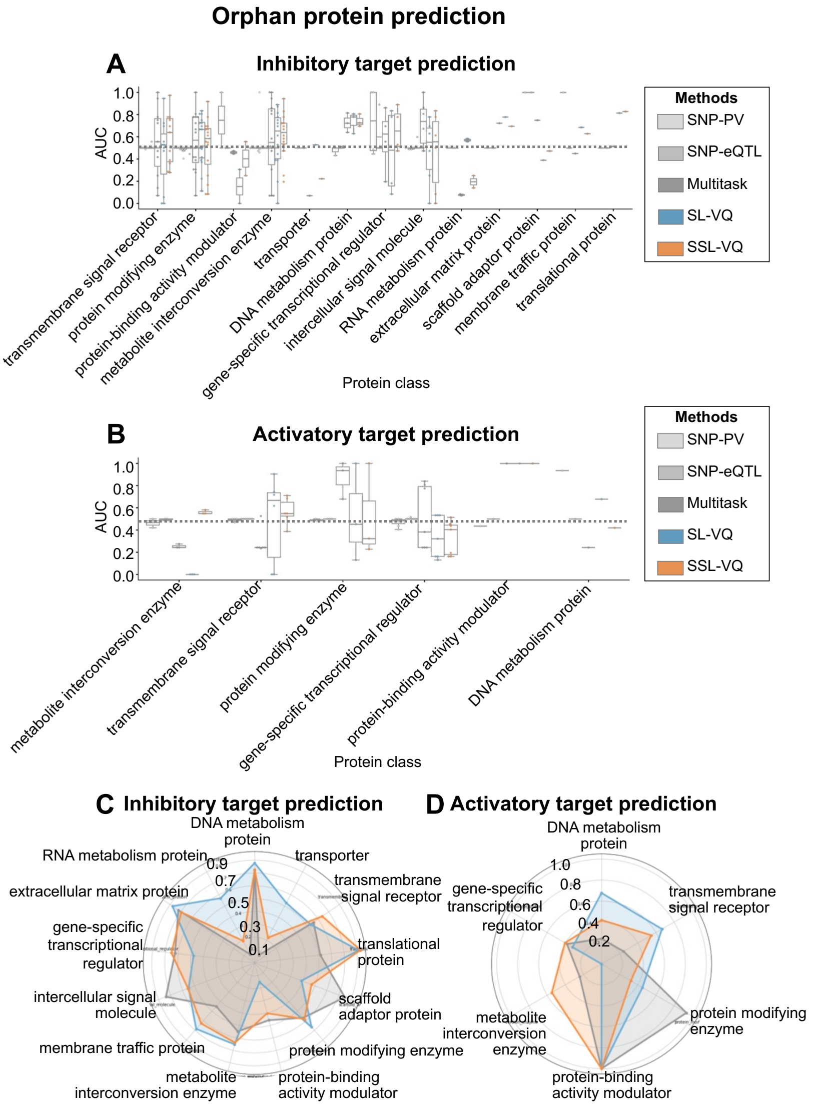

Supplementary Fig. S6: Performance evaluation of predicting therapeutic indications for uncharacterized proteins for each protein class.

(A) Comparison of the performance of the proposed methods and baseline methods for predicting inhibitory targets for 24 diseases and 63 proteins; the proposed methods correspond to the SSL-VQ and SL-VQ. The baseline method corresponds to multitask learning method. Models were trained and evaluated based on a "uncharacterized protein" scenario (Methods). The boxplots represent the distributions of AUC scores for each protein class. Protein class information was obtained from PANTHER database (https://www.pantherdb.org/). In the box plots: center line, median; box, interquartile range; whiskers, 1.5× interquartile range; and point, AUC score for each protein. Colors represent prediction methods. (B) Same as (A) but for activatory target predictions for 18 diseases and 21 proteins. (C) Same as (A) but radar chart for each protein class. Orange, blue, and gray lines represent SSL-VQ, SL-VQ and multitask learning method, respectively. (D) Same as (A) but for activatory target predictions for 18 diseases and 21 proteins.

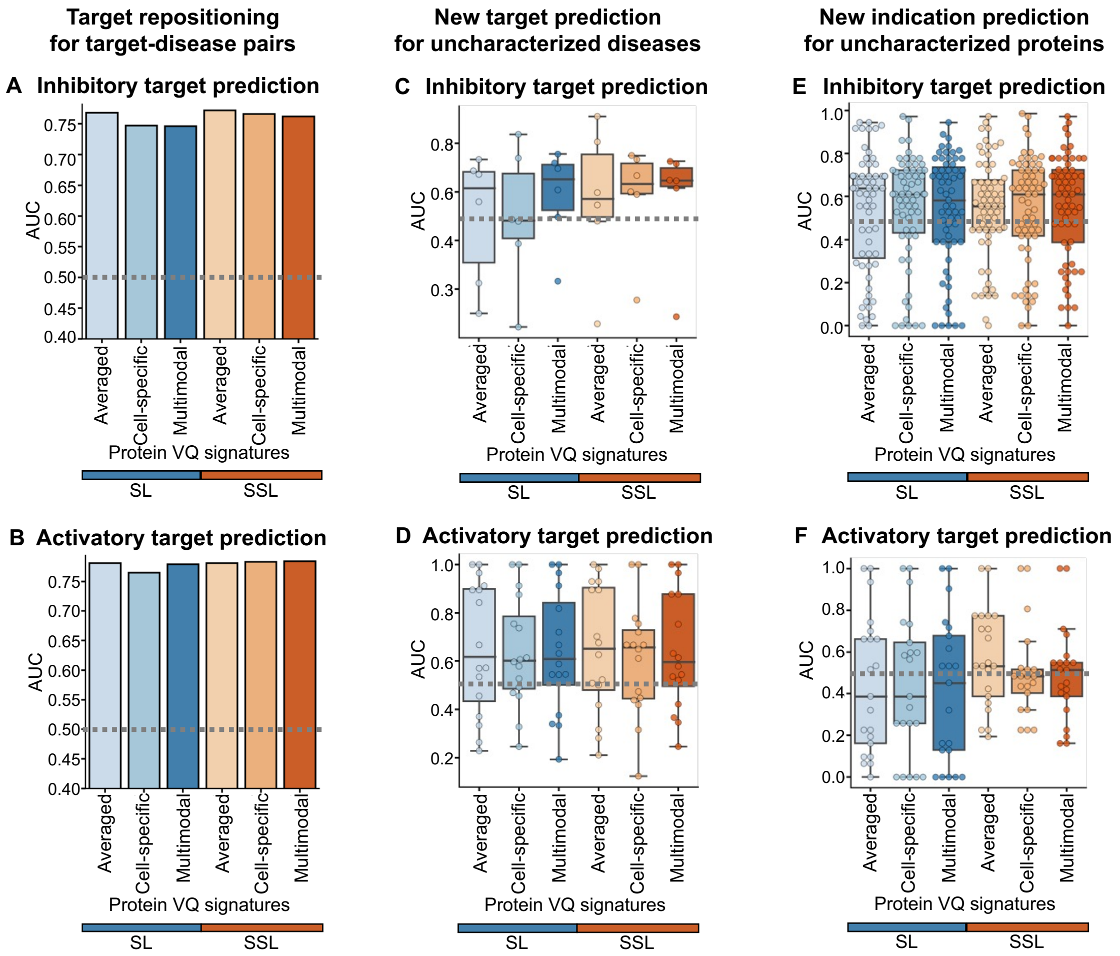

Supplementary Fig. S7: Performance comparison between feature extraction processes from multiple cells based on AUC.

(A) Target repositioning for inhibitory target-disease pairs. SL and SSL were used. Protein VQ signatures include protein multimodal VQ signatures (Multimodal), protein cell-specific VQ signatures (Cell-specific), and protein averaged VQ signatures (Averaged). Models were trained and evaluated based on the "target repositioning for target-disease pairs" scenario. (B) As described for (A), but for activatory target-disease pairs. (C) Performance evaluation of new inhibitory target prediction for uncharacterized diseases. Models were trained and evaluated based on the "new target prediction for uncharacterized diseases" scenario. Boxplots represent AUC score distributions for each disease. (D) As described for (C), but for activatory targets. (E) Performance evaluation of new inhibitory indication predictions for uncharacterized proteins. Models were trained and evaluated based on the "new indication prediction for uncharacterized proteins" scenario. (F) As described for (E), but for activatory targets.

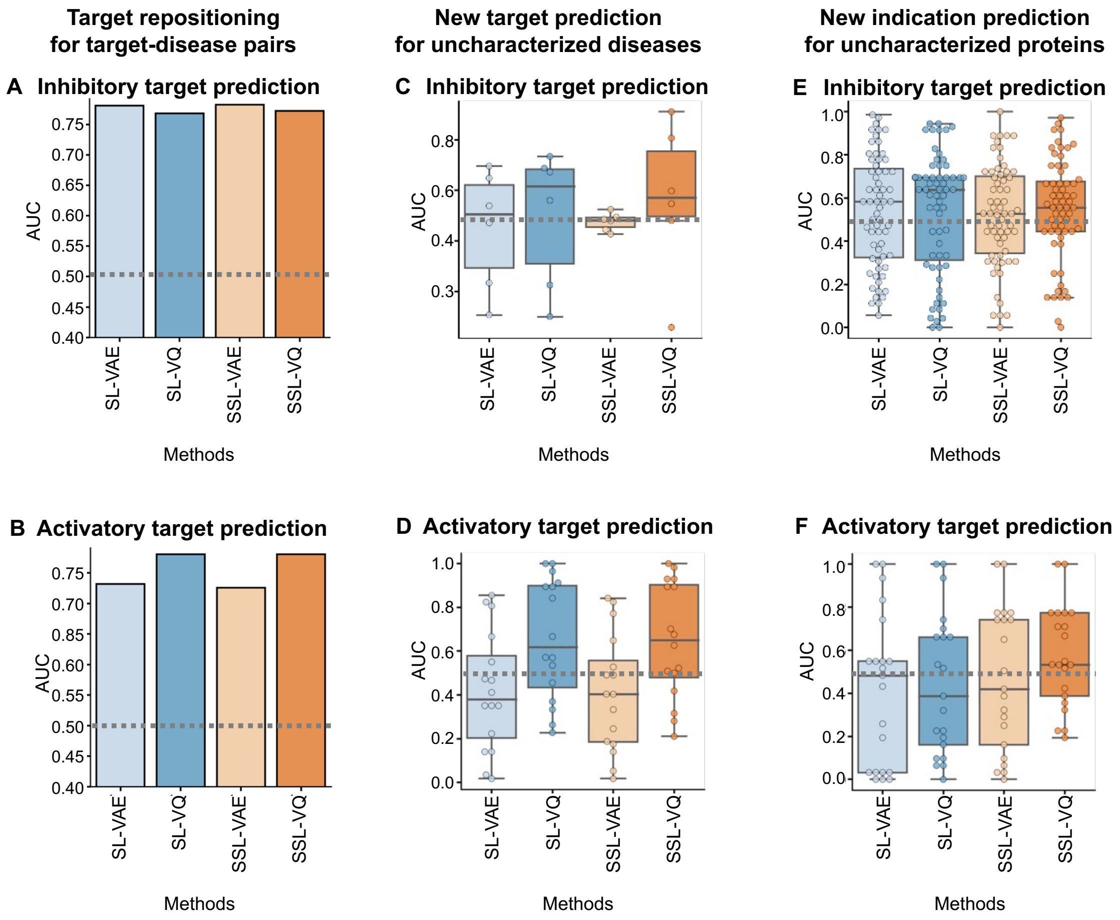

Supplementary Fig. S8: Performance comparison between VAE signatures and VQ-VAE signatures. Target repositioning for inhibitory target-disease pairs. SL and SSL were used. VAE signatures represent the case where protein VAE signatures and disease VAE signatures are used; VQ signatures represent the case where protein averaged VQ signatures and disease VQ signatures are used. Models were trained and evaluated based on "target repositioning for target-disease pairs" scenario. (B) As described for (A), but for activatory target-disease pairs. (C) Performance evaluation of new inhibitory target prediction for uncharacterized diseases. Models were trained and evaluated based on the "new target prediction for uncharacterized diseases" scenario. Boxplots represent AUC score distributions for each disease. (D) As described for (C), but for activatory targets. (E) Performance evaluation of new inhibitory indication predictions for uncharacterized proteins. Models were trained and evaluated based on the "new indication prediction for uncharacterized proteins" scenario. (F) As described for (E), but for activatory targets.

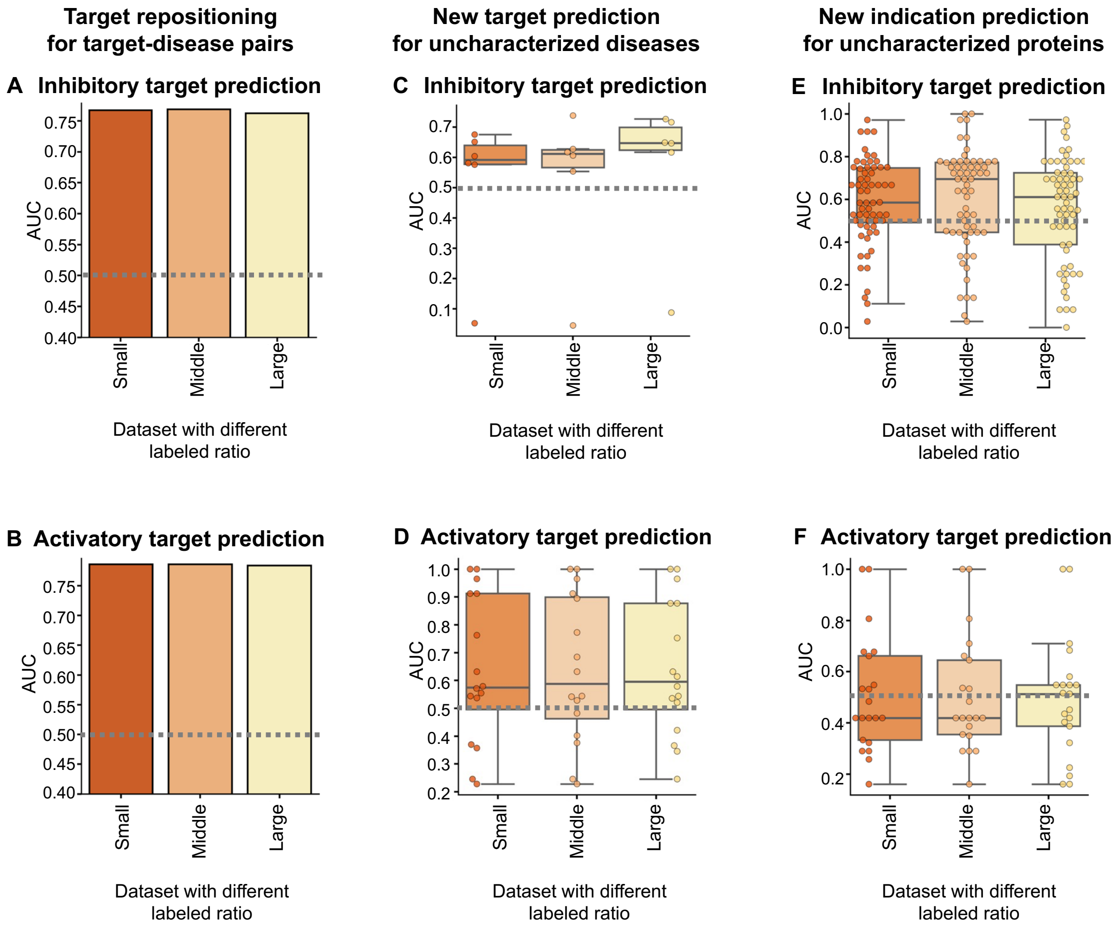

Supplementary Fig. S9: Performance comparisons based on AUC when trained on datasets with different amounts of unlabeled samples.

(A) Target repositioning for inhibitory target-disease pairs by SSL-VQ. Models were trained and evaluated based on the "target repositioning for target-disease pairs" scenario. The method's performance was compared across the "Small," "Middle," and "Large." The orange bars represent the "Small" dataset, the light orange bars represent the "Middle" dataset, and the light yellow bars represent the "Large" dataset. (B) As described for (A), but for activatory target-disease pairs. (C) Performance evaluation of new inhibitory target prediction for uncharacterized diseases. Models were trained and evaluated based on the "new target prediction for uncharacterized diseases" scenario. Boxplots represent AUC score distributions for diseases. (D) As described for (C), but for activatory targets. (E) Performance evaluation of new inhibitory indication predictions for uncharacterized proteins. Models were trained and evaluated based on the "new indication prediction for uncharacterized proteins" scenario. (F) As described for (E), but for activatory targets.

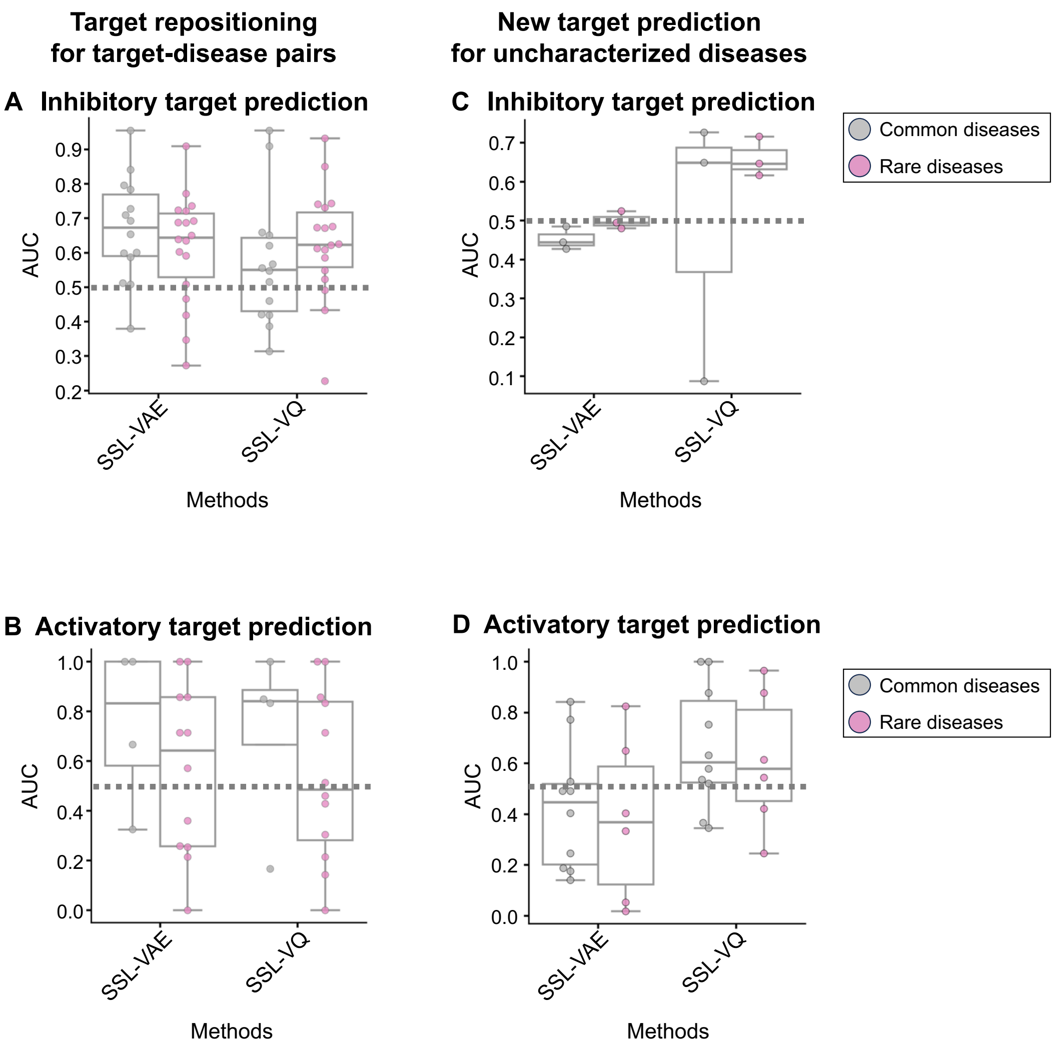

Supplementary Fig. S10: Performance evaluation of the proposed methods for common diseases and rare diseases.

(A) Target repositioning for inhibitory target–disease pairs. Models were trained and evaluated based on the "target repositioning for target-disease pairs" scenario. The AUC scores were compared between common and rare diseases. The gray dots represent the common diseases, the pink dots represent the rare diseases. (B) As described for (A), but for activatory target–disease pairs. (C) Performance evaluation of new inhibitory target prediction for uncharacterized diseases. Models were trained and evaluated based on the "new target prediction for uncharacterized diseases" scenario. Boxplots represent AUC score distributions for diseases. (D) As described for (C), but for activatory targets.

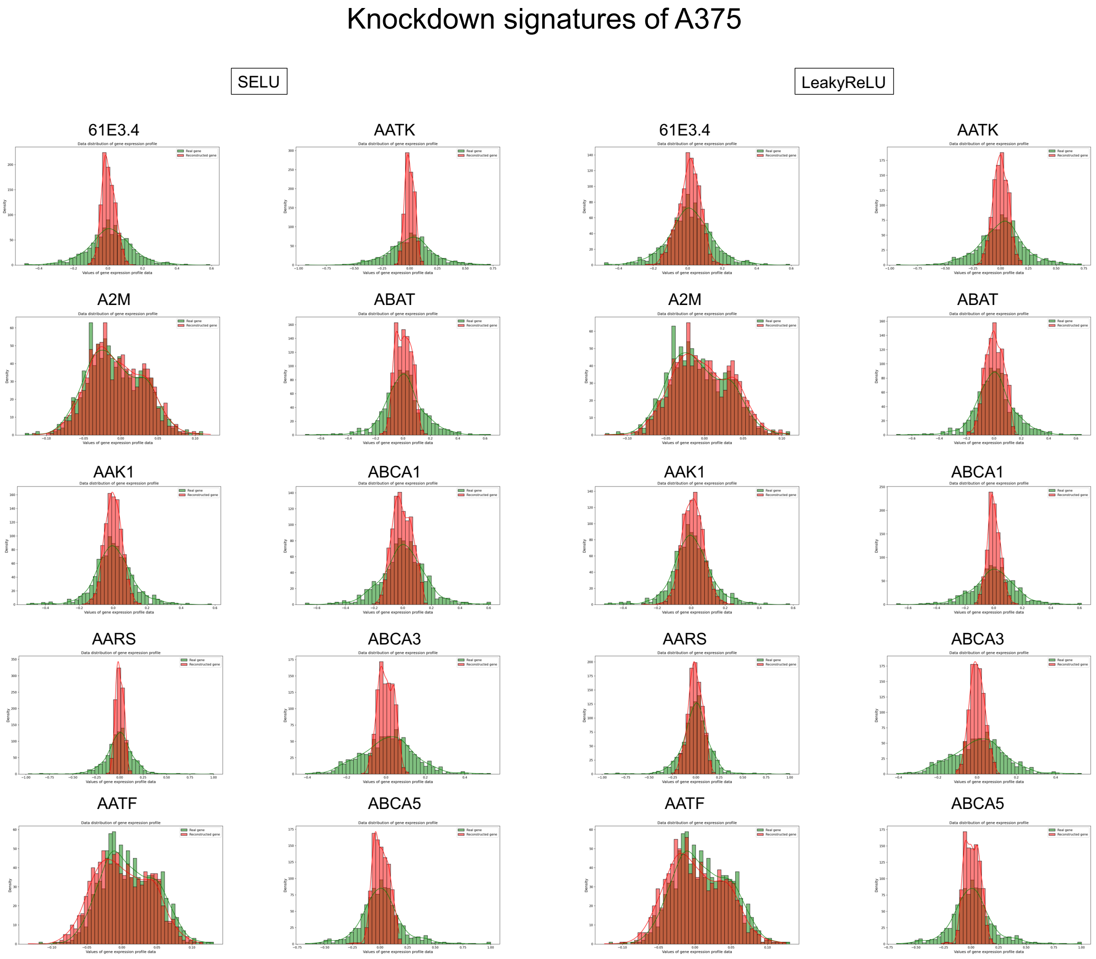

Supplementary Fig. S11: Distribution of original and reconstructed gene knockdown signatures of A375.

The left 10 panels show original and reconstructed signatures using multimodal VQ-VAE with SELU activation function. The right 10 panels show original and reconstructed signatures using multimodal VQ-VAE with LeakyReLU. Horizontal and vertical axes represent gene expression scores and frequency. Green and red represent original and reconstructed signatures, respectively.

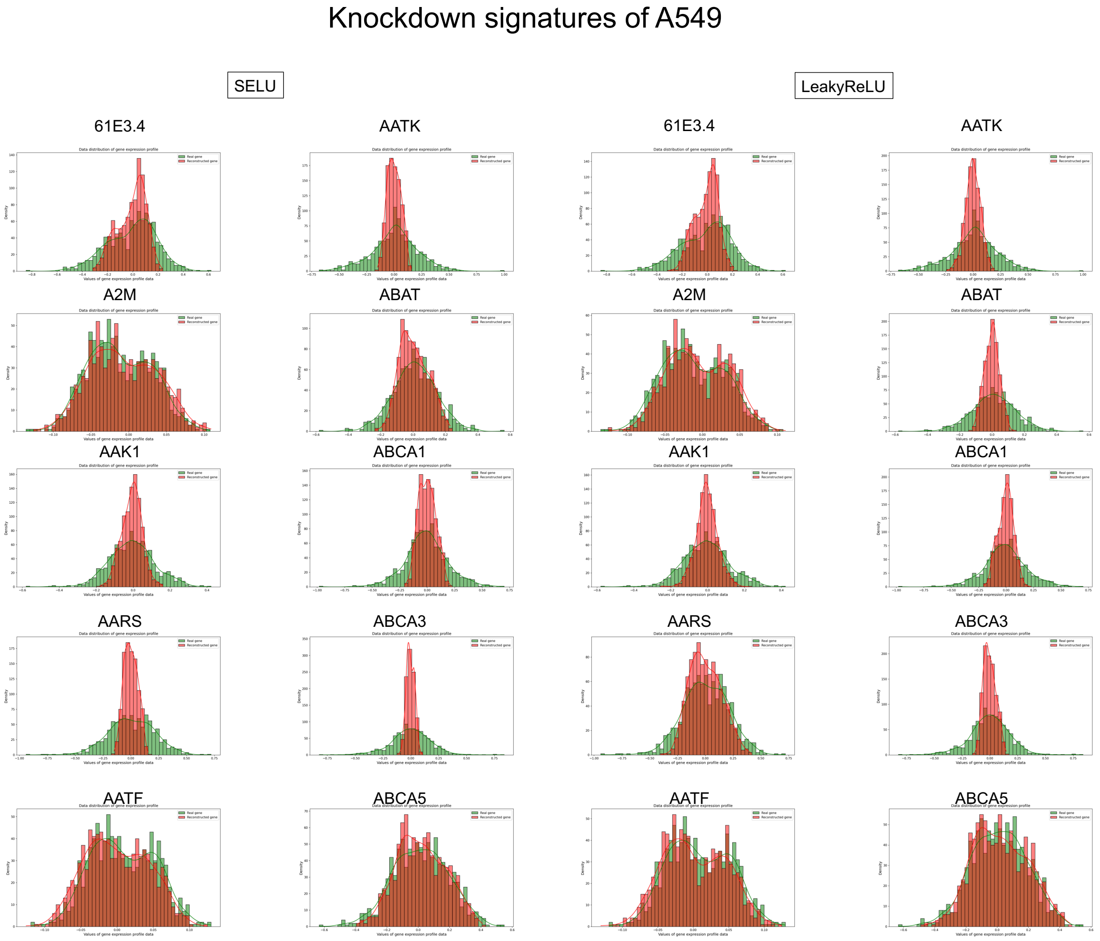

Supplementary Fig. S12: Distribution of original and reconstructed gene knockdown signatures of A549.

The left 10 panels show original and reconstructed signatures using multimodal VQ-VAE with SELU activation function. The right 10 panels show original and reconstructed signatures using multimodal VQ-VAE with LeakyReLU. Horizontal and vertical axes represent gene expression scores and frequency. Green and red represent original and reconstructed signatures, respectively.

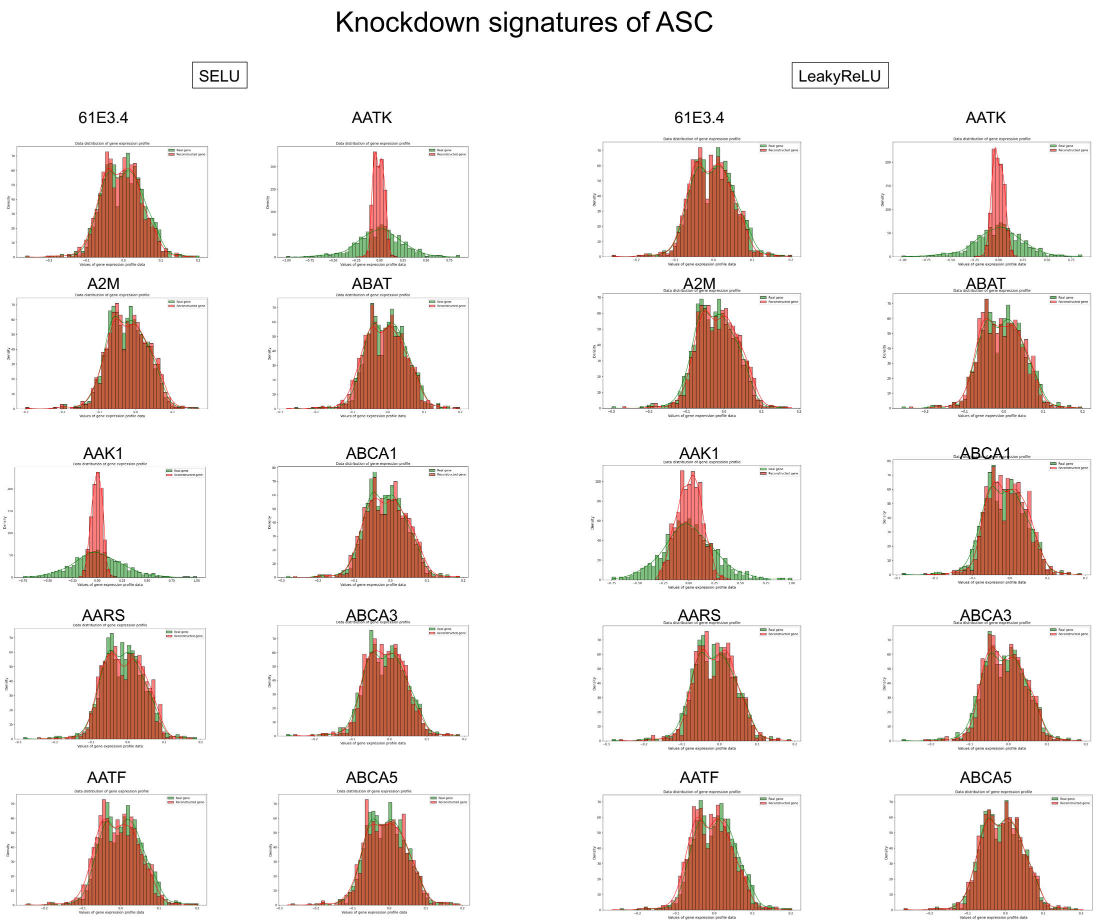

Supplementary Fig. S13: Distribution of original and reconstructed gene knockdown signatures of ASC.

The left 10 panels show original and reconstructed signatures using multimodal VQ-VAE with SELU activation function. The right 10 panels show original and reconstructed signatures using multimodal VQ-VAE with LeakyReLU. Horizontal and vertical axes represent gene expression scores and frequency. Green and red represent original and reconstructed signatures, respectively.

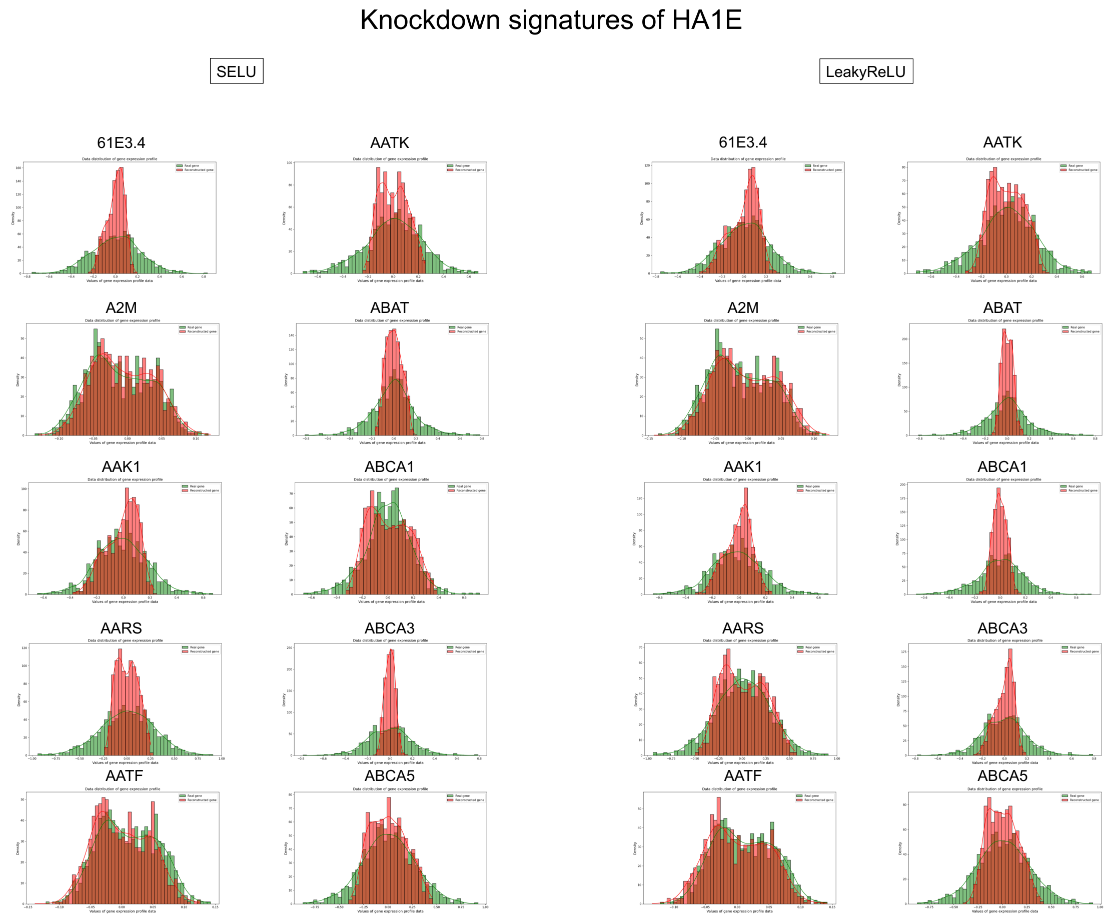

Supplementary Fig. S14: Distribution of original and reconstructed gene knockdown signatures of HA1E.

The left 10 panels show original and reconstructed signatures using multimodal VQ-VAE with SELU activation function. The right 10 panels show original and reconstructed signatures using multimodal VQ-VAE with LeakyReLU. Horizontal and vertical axes represent gene expression scores and frequency. Green and red represent original and reconstructed signatures, respectively.

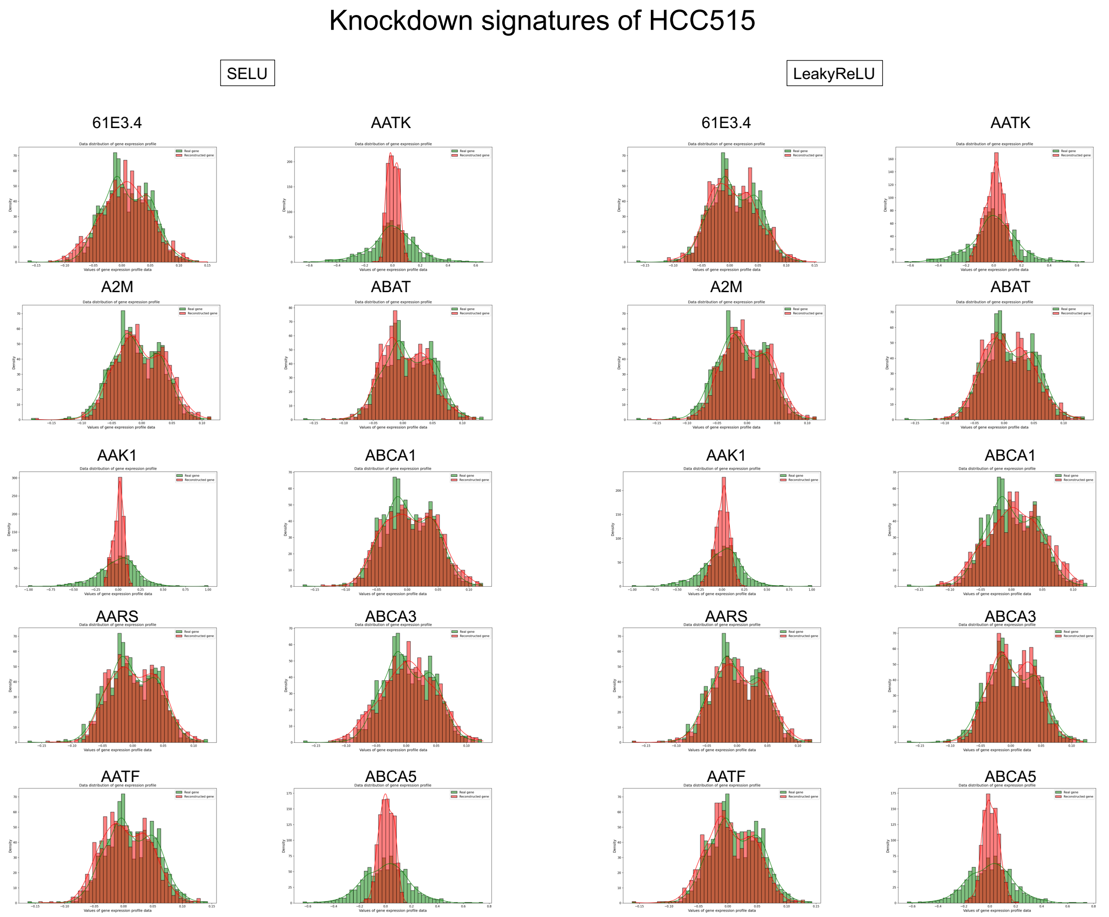

Supplementary Fig. S15: Distribution of original and reconstructed gene knockdown signatures of HCC515.

The left 10 panels show original and reconstructed signatures using multimodal VQ-VAE with SELU activation function. The right 10 panels show original and reconstructed signatures using multimodal VQ-VAE with LeakyReLU. Horizontal and vertical axes represent gene expression scores and frequency. Green and red represent original and reconstructed signatures, respectively.

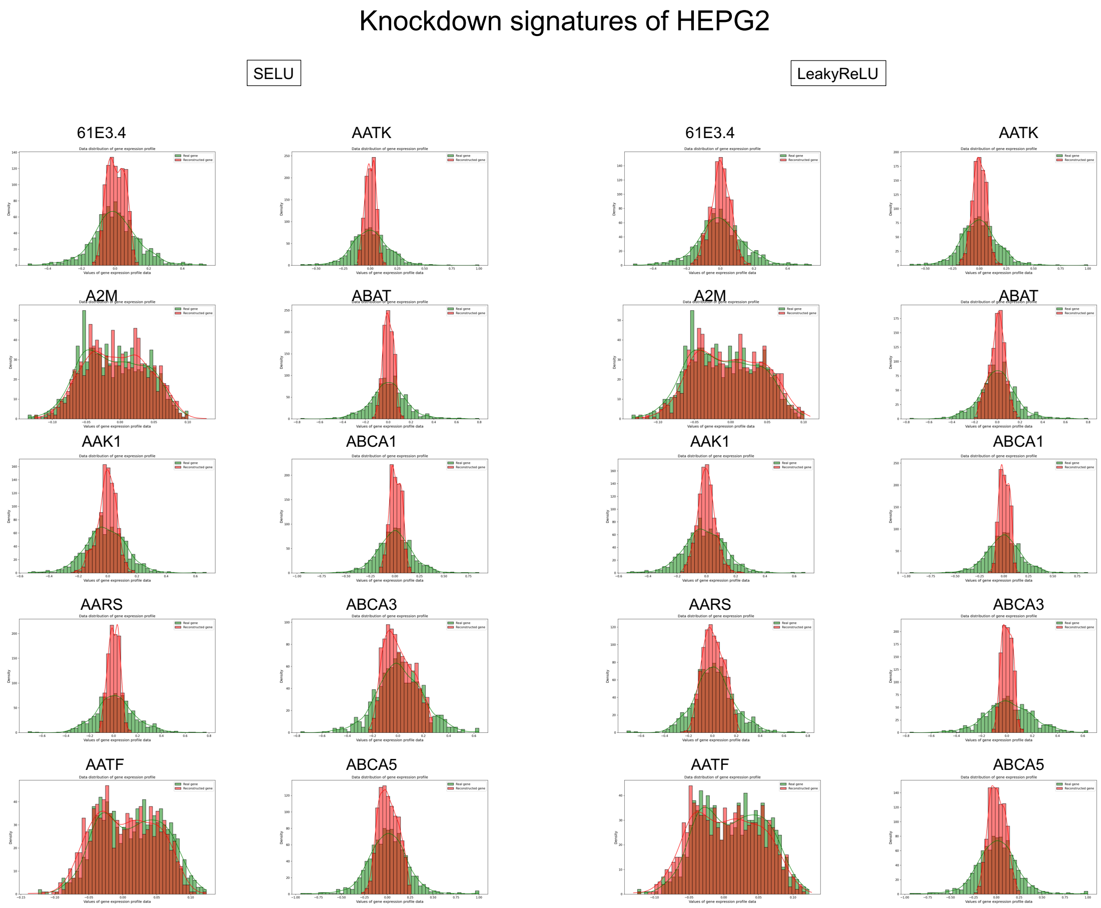

Supplementary Fig. S16: Distribution of original and reconstructed gene knockdown signatures of HEPG2.

The left 10 panels show original and reconstructed signatures using multimodal VQ-VAE with SELU activation function. The right 10 panels show original and reconstructed signatures using multimodal VQ-VAE with LeakyReLU. Horizontal and vertical axes represent gene expression scores and frequency. Green and red represent original and reconstructed signatures, respectively.

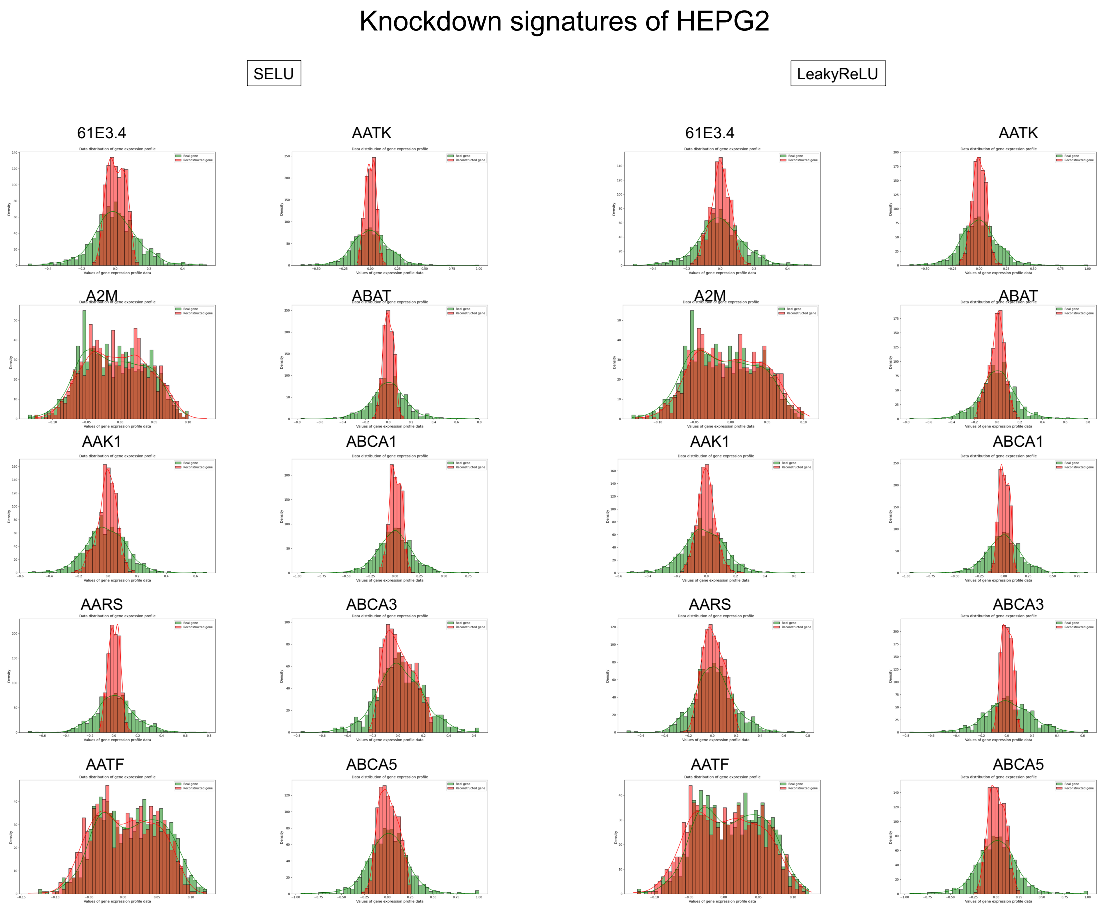

Supplementary Fig. S17: Distribution of original and reconstructed gene knockdown signatures of HEPG2.

The left 10 panels show original and reconstructed signatures using multimodal VQ-VAE with SELU activation function. The right 10 panels show original and reconstructed signatures using multimodal VQ-VAE with LekyReLU. Horizontal and vertical axes represent gene expression scores and frequency. Green and red represent original and reconstructed signatures, respectively.

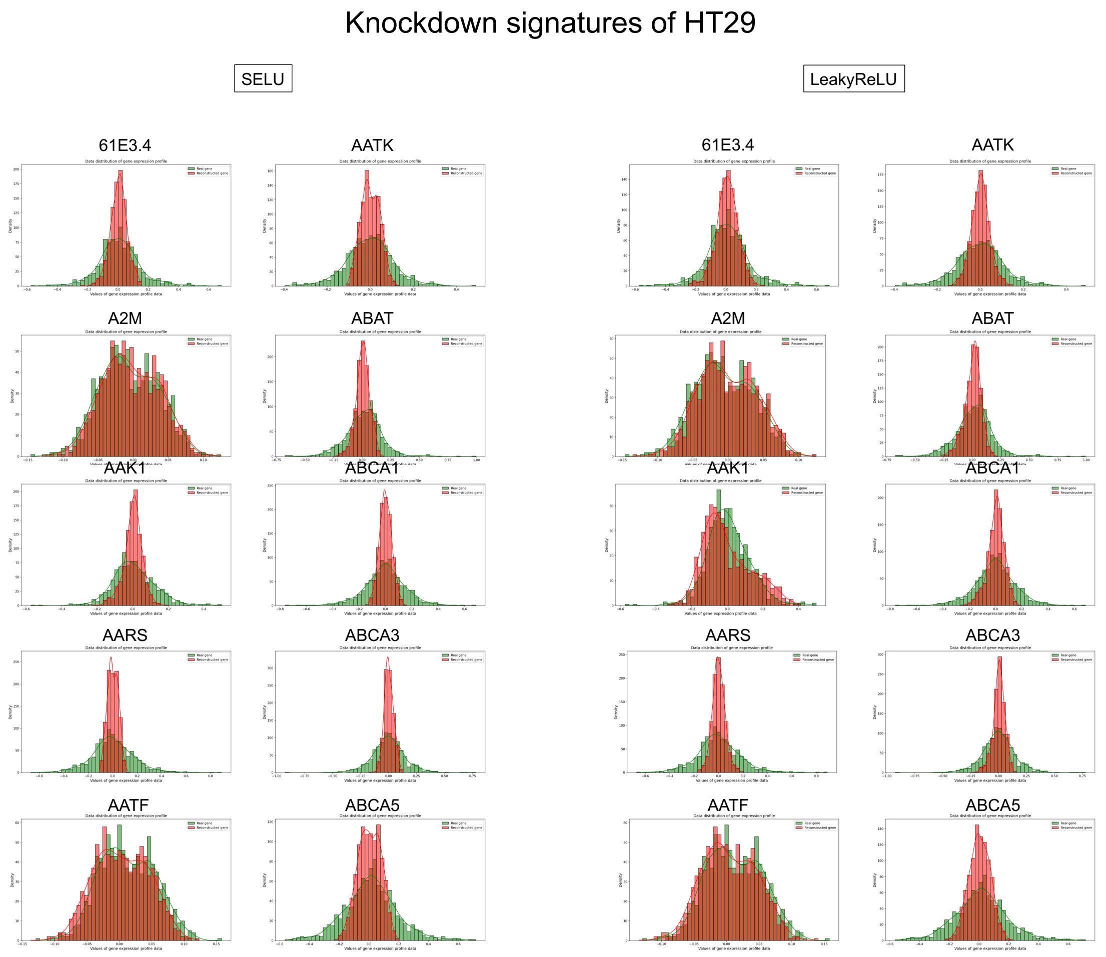

Supplementary Fig. S18: Distribution of original and reconstructed gene knockdown signatures of HT29.

The left 10 panels show original and reconstructed signatures using multimodal VQ-VAE with SELU activation function. The right 10 panels show original and reconstructed signatures using multimodal VQ-VAE with LeakyReLU. Horizontal and vertical axes represent gene expression scores and frequency. Green and red represent original and reconstructed signatures, respectively.

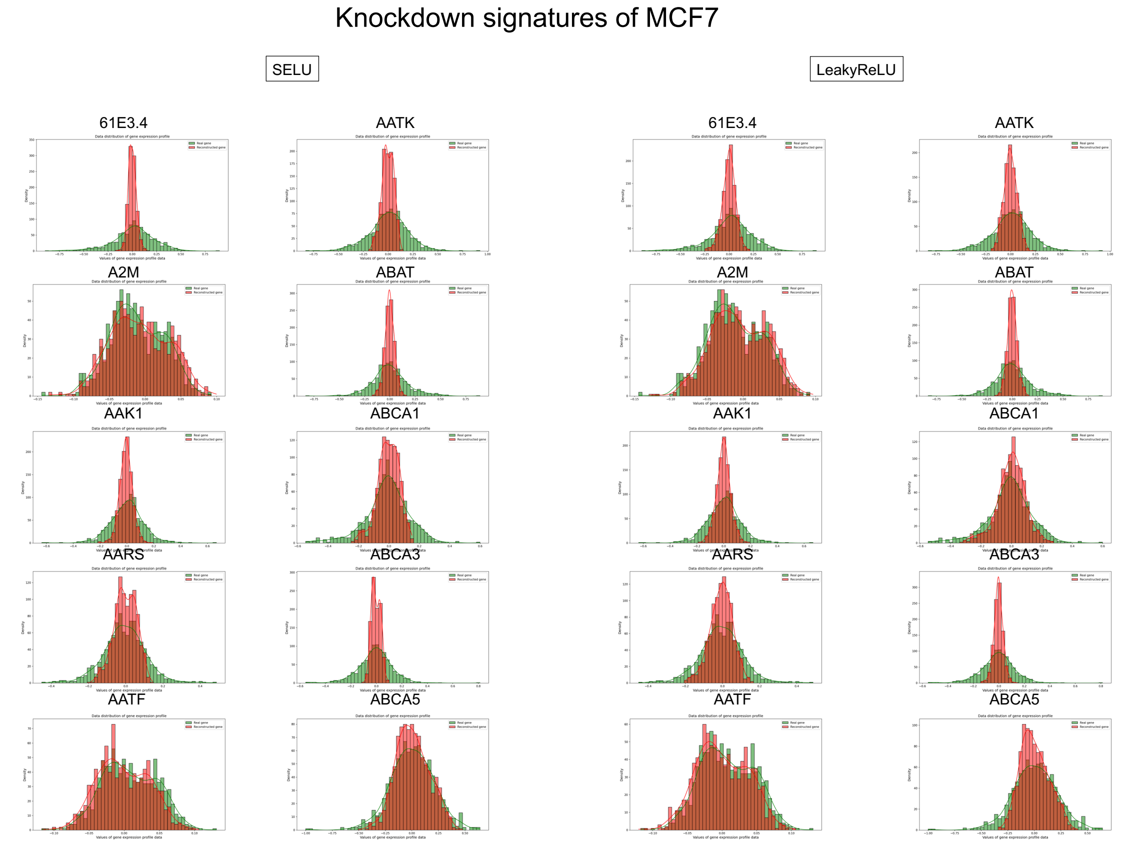

Supplementary Fig. S19: Distribution of original and reconstructed gene knockdown signatures of MCF7.

The left 10 panels show original and reconstructed signatures using multimodal VQ-VAE with SELU activation function. The right 10 panels show original and reconstructed signatures using multimodal VQ-VAE with LeakyReLU. Horizontal and vertical axes represent gene expression scores and frequency. Green and red represent original and reconstructed signatures, respectively.

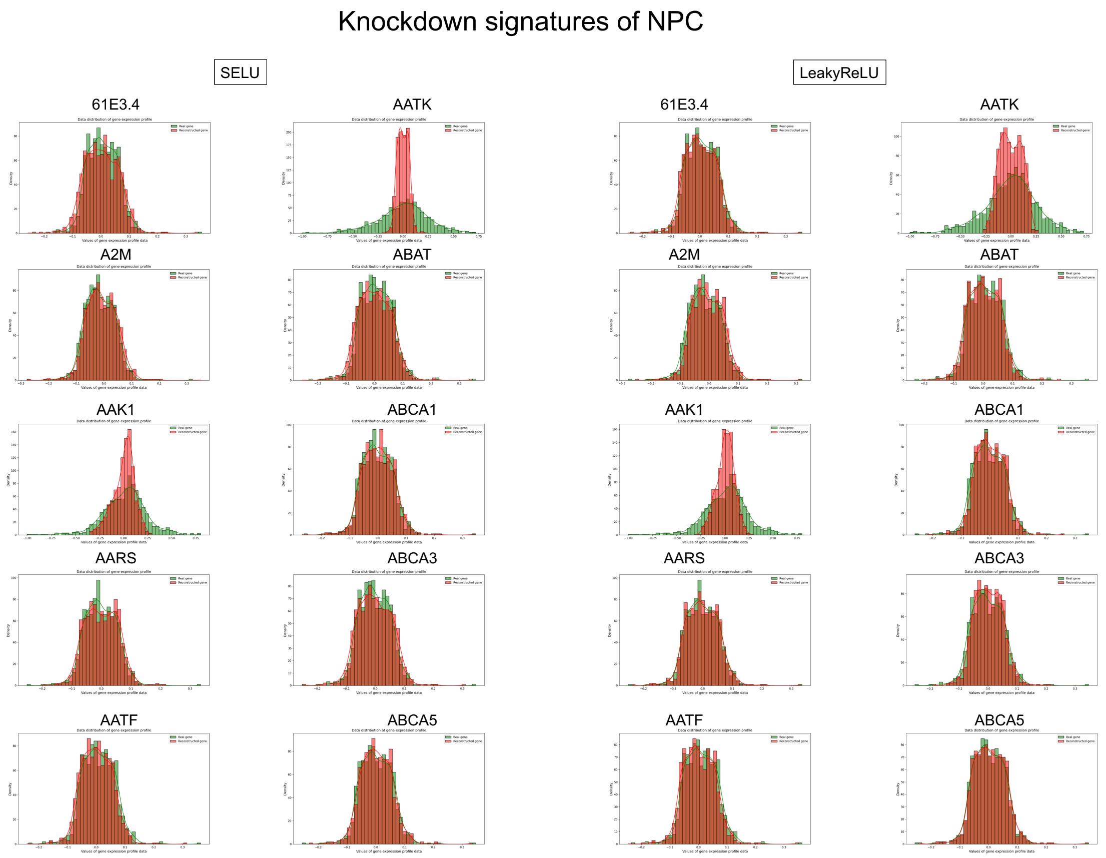

Supplementary Fig. S20: Distribution of original and reconstructed gene knockdown signatures of NPC.

The left 10 panels show original and reconstructed signatures using multimodal VQ-VAE with SELU activation function. The right 10 panels show original and reconstructed signatures using multimodal VQ-VAE with LeakyReLU. Horizontal and vertical axes represent gene expression scores and frequency. Green and red represent original and reconstructed signatures, respectively.

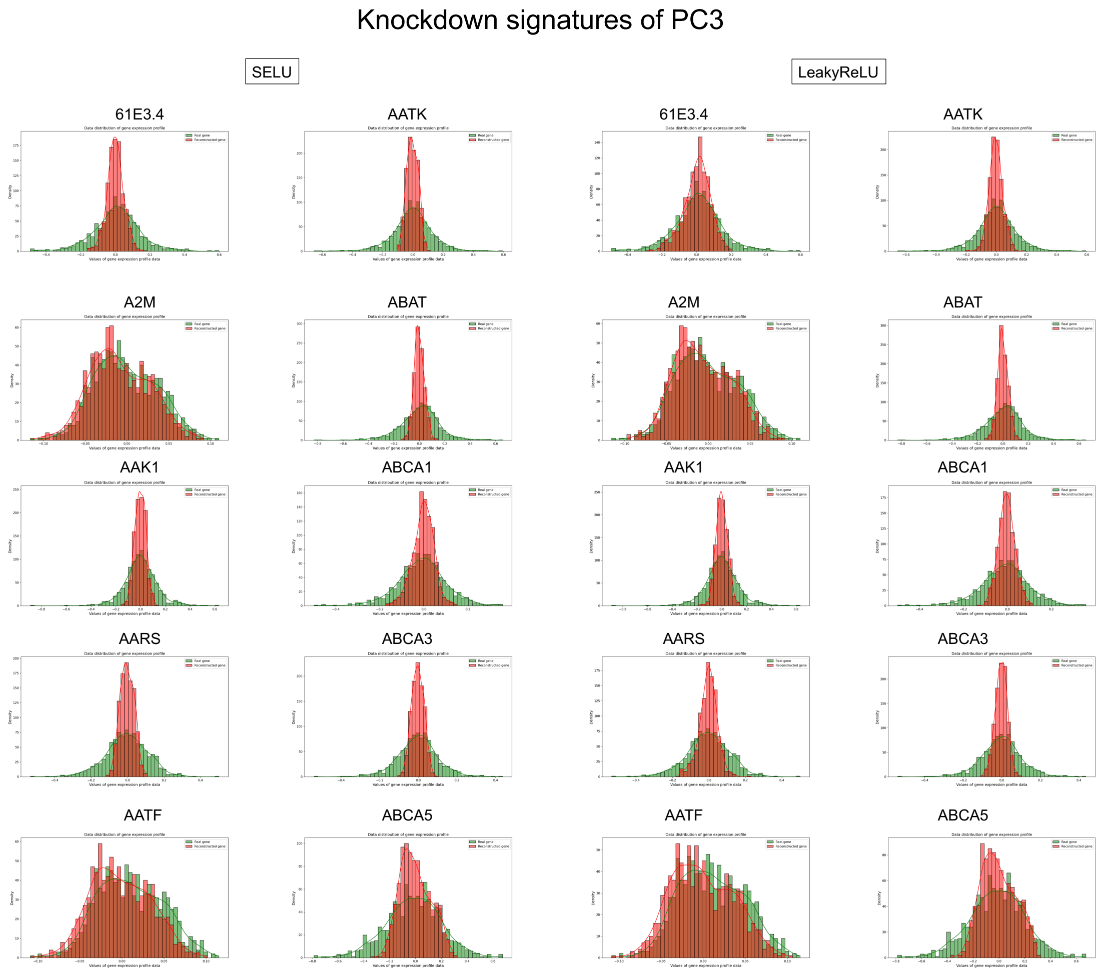

Supplementary Fig. S21: Distribution of original and reconstructed gene knockdown signatures of PC3.

The left 10 panels show original and reconstructed signatures using multimodal VQ-VAE with SELU activation function. The right 10 panels show original and reconstructed signatures using multimodal VQ-VAE with LeakyReLU. Horizontal and vertical axes represent gene expression scores and frequency. Green and red represent original and reconstructed signatures, respectively.

Knockdown signatures of VCAP

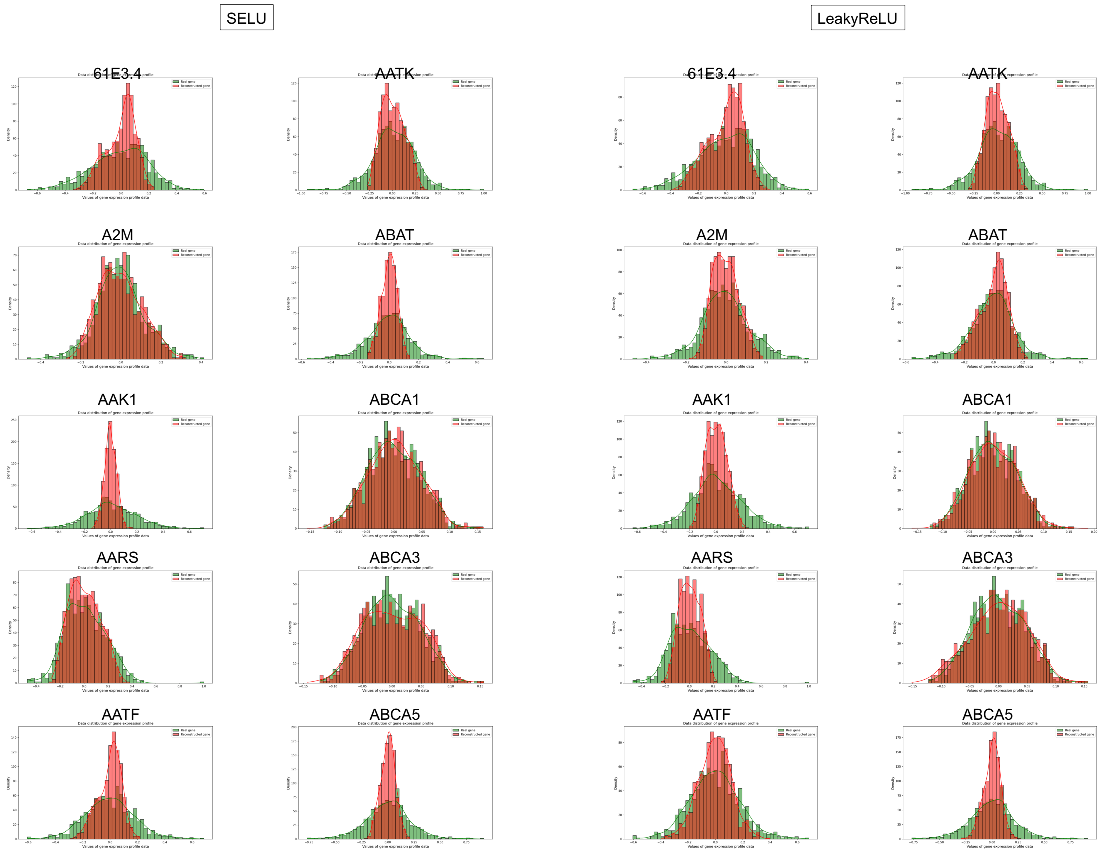

Supplementary Fig. S22: Distribution of original and reconstructed gene knockdown signatures of VCAP.

The left 10 panels show original and reconstructed signatures using multimodal VQ-VAE with SELU activation function. The right 10 panels show original and reconstructed signatures using multimodal VQ-VAE with LeakyReLU. Horizontal and vertical axes represent gene expression scores and frequency. Green and red represent original and reconstructed signatures, respectively.

Overexpression signatures of A375

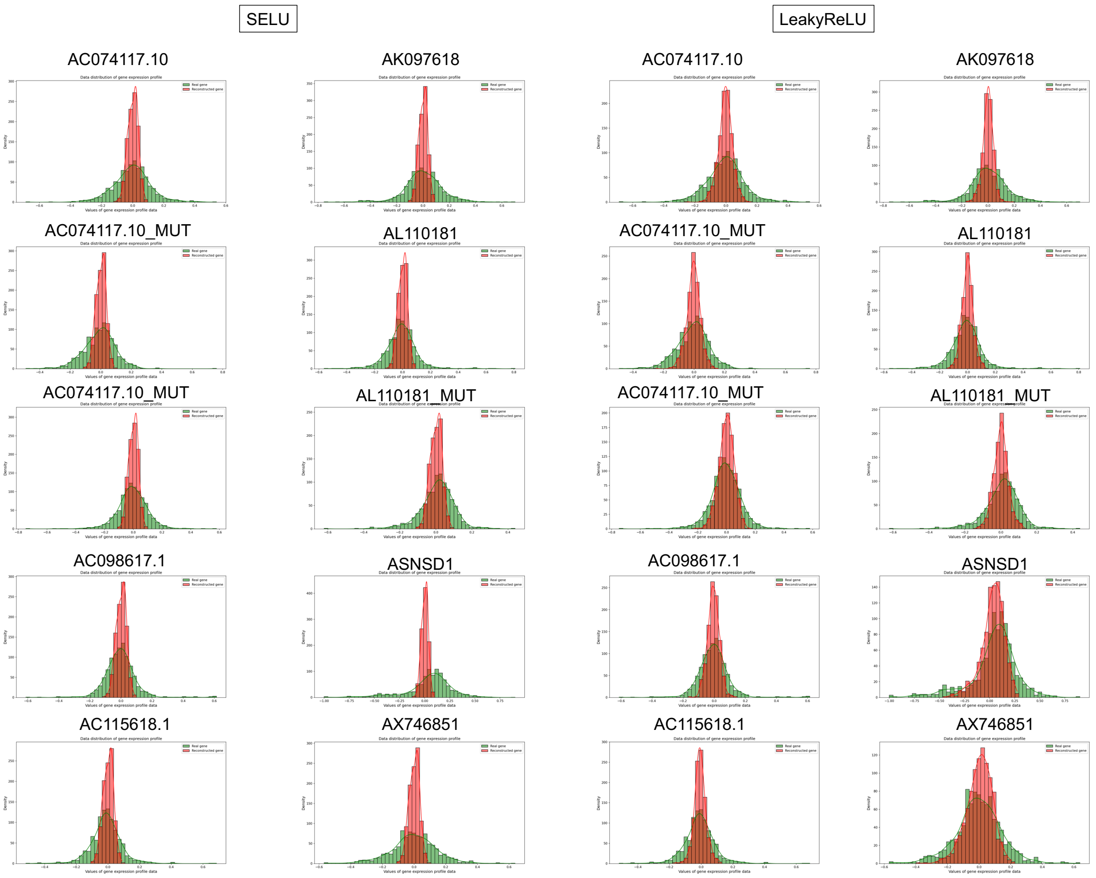

Supplementary Fig. S23: Distribution of original and reconstructed gene overexpression signatures of A375.

The left 10 panels show original and reconstructed signatures using multimodal VQ-VAE with SELU activation function. The right 10 panels show original and reconstructed signatures using multimodal VQ-VAE with LeakyReLU. Horizontal and vertical axes represent gene expression scores and frequency. Green and red represent original and reconstructed signatures, respectively.

Overexpression signatures of A549

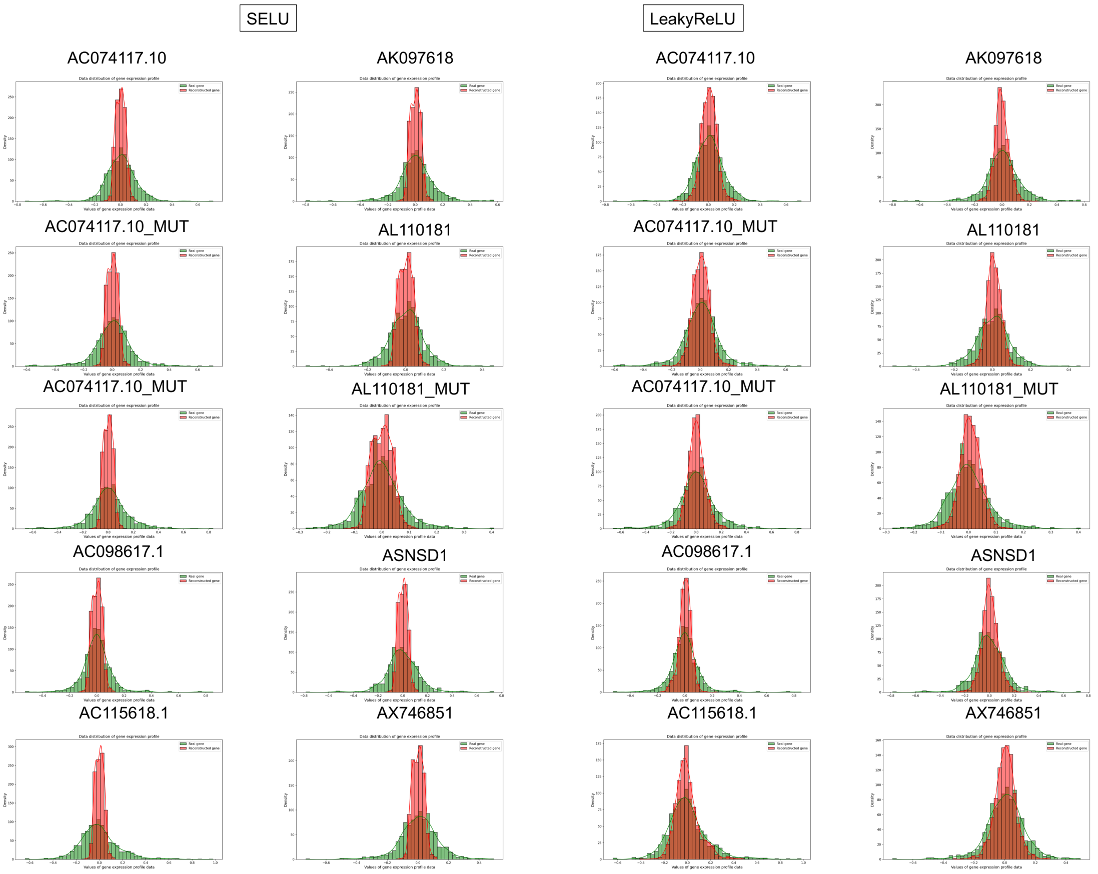

Supplementary Fig. S24: Distribution of original and reconstructed gene overexpression signatures of A549.

The left 10 panels show original and reconstructed signatures using multimodal VQ-VAE with SELU activation function. The right 10 panels show original and reconstructed signatures using multimodal VQ-VAE with LeakyReLU. Horizontal and vertical axes represent gene expression scores and frequency. Green and red represent original and reconstructed signatures, respectively.

Overexpression signatures of HA1E

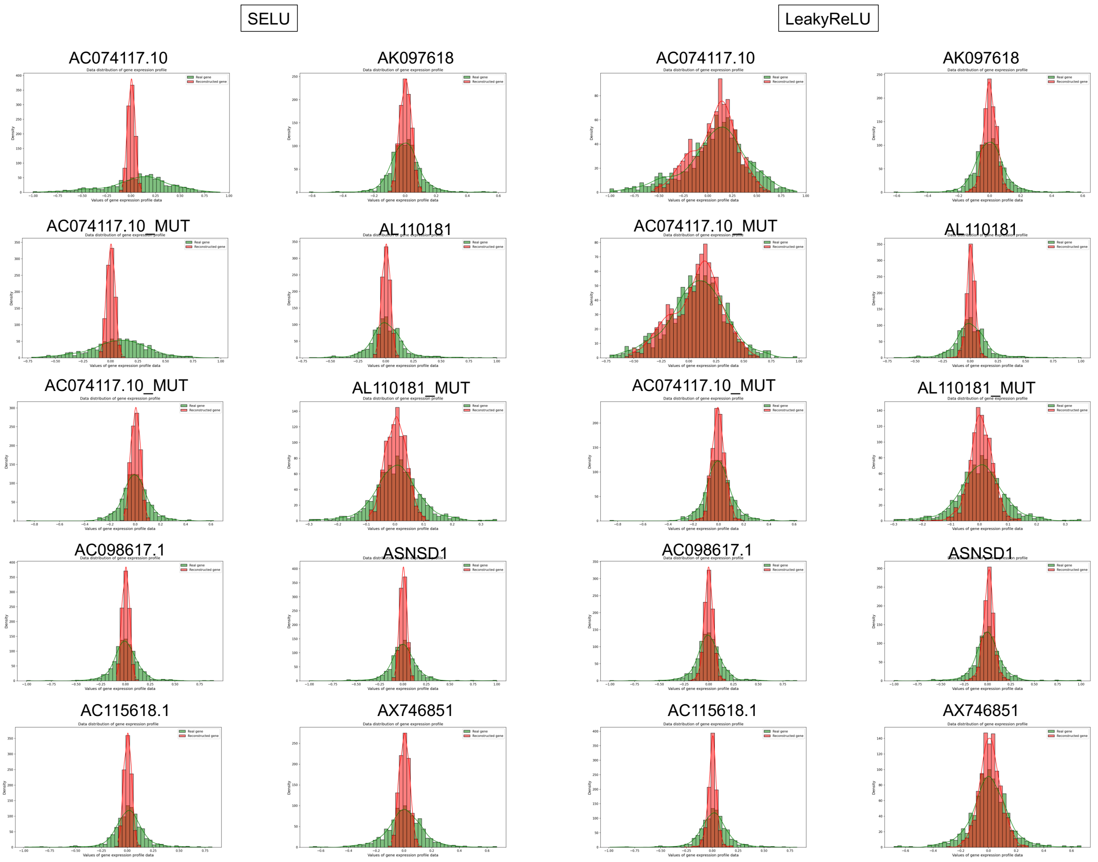

Supplementary Fig. S25: Distribution of original and reconstructed gene overexpression signatures of HA1E.

The left 10 panels show original and reconstructed signatures using multimodal VQ-VAE with SELU activation function. The right 10 panels show original and reconstructed signatures using multimodal VQ-VAE with LeakyReLU. Horizontal and vertical axes represent gene expression scores and frequency. Green and red represent original and reconstructed signatures, respectively.

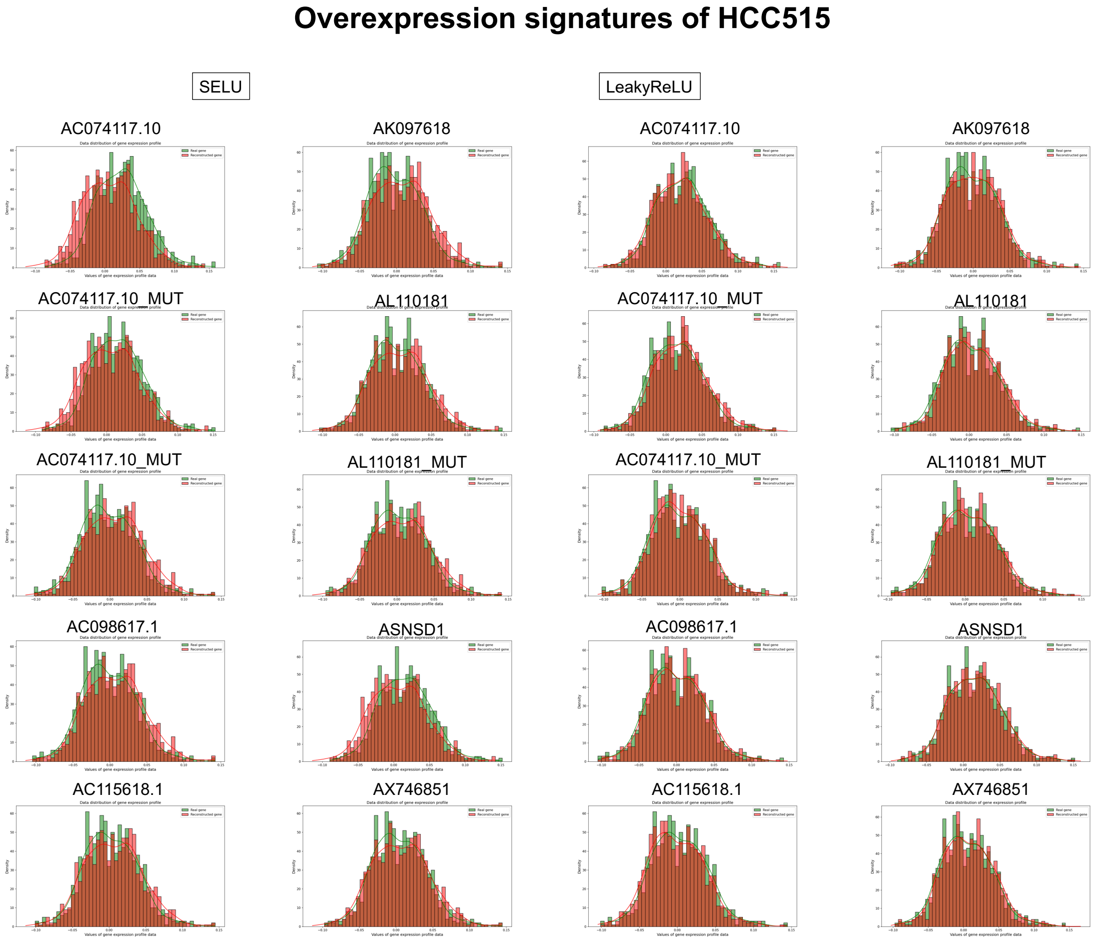

Supplementary Fig. S26: Distribution of original and reconstructed gene overexpression signatures of HCC515.

The left 10 panels show original and reconstructed signatures using multimodal VQ-VAE with SELU activation function. The right 10 panels show original and reconstructed signatures using multimodal VQ-VAE with LeakyReLU. Horizontal and vertical axes represent gene expression scores and frequency. Green and red represent original and reconstructed signatures, respectively.

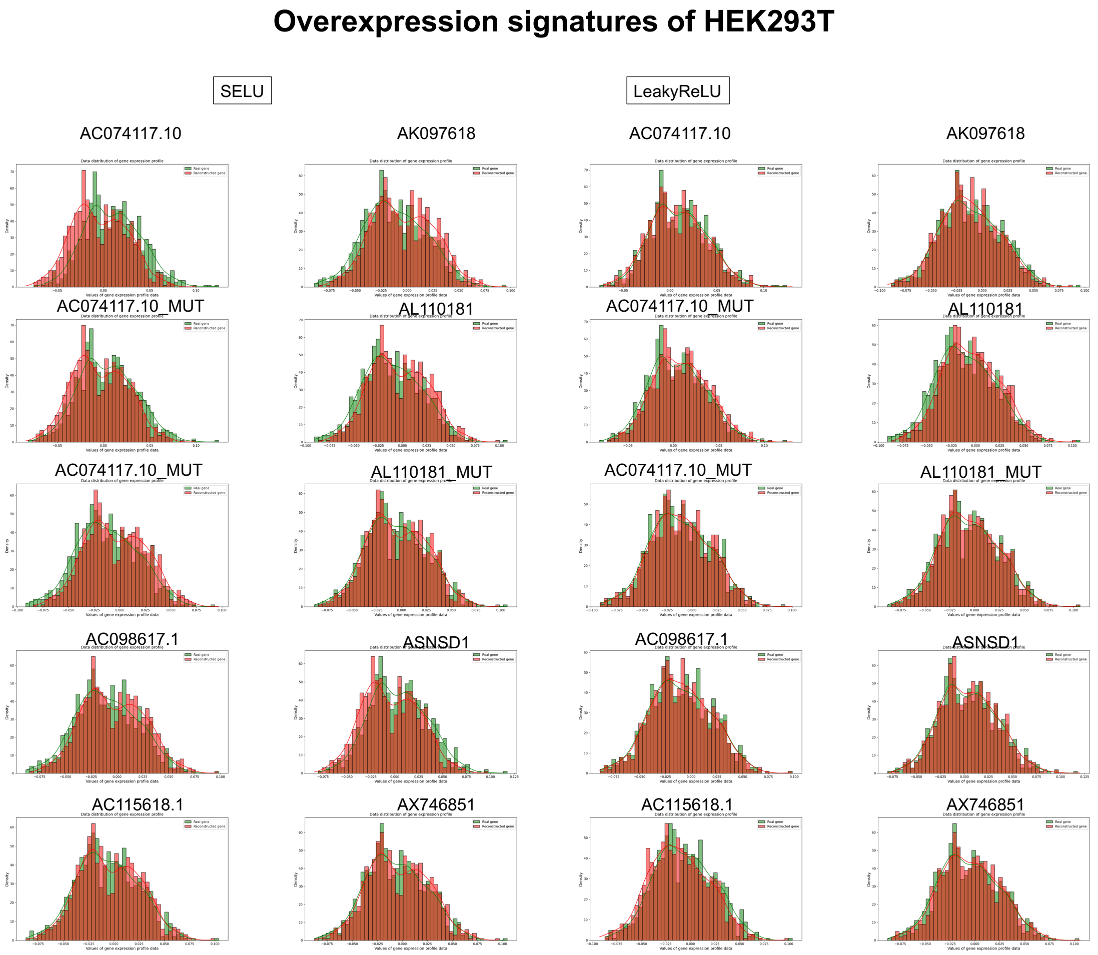

Supplementary Fig. S27: Distribution of original and reconstructed gene overexpression signatures of HEK293T.

The left 10 panels show original and reconstructed signatures using multimodal VQ-VAE with SELU activation function. The right 10 panels show original and reconstructed signatures using multimodal VQ-VAE with LeakyReLU. Horizontal and vertical axes represent gene expression scores and frequency. Green and red represent original and reconstructed signatures, respectively.

Overexpression signatures of HEPG2

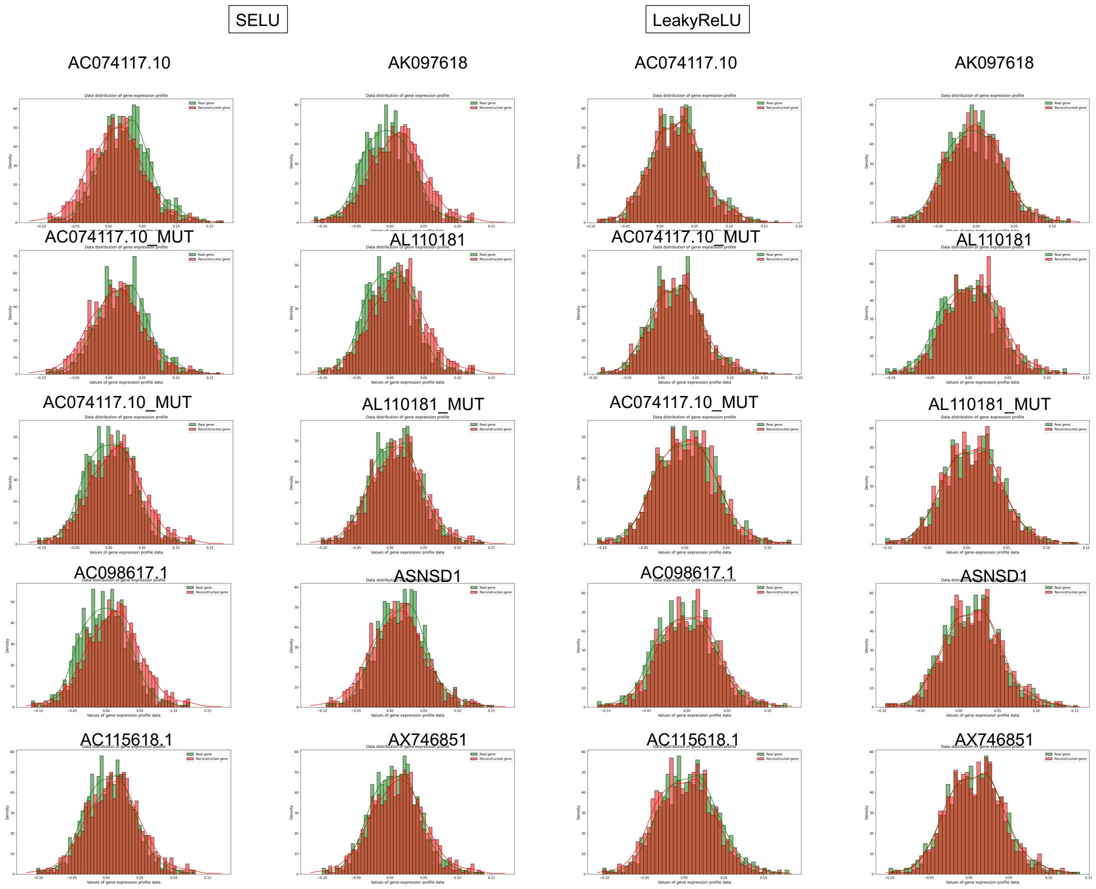

Supplementary Fig. S28: Distribution of original and reconstructed gene overexpression signatures of HEPG2.

The left 10 panels show original and reconstructed signatures using multimodal VQ-VAE with SELU activation function. The right 10 panels show original and reconstructed signatures using multimodal VQ-VAE with LeakyReLU. Horizontal and vertical axes represent gene expression scores and frequency. Green and red represent original and reconstructed signatures, respectively.

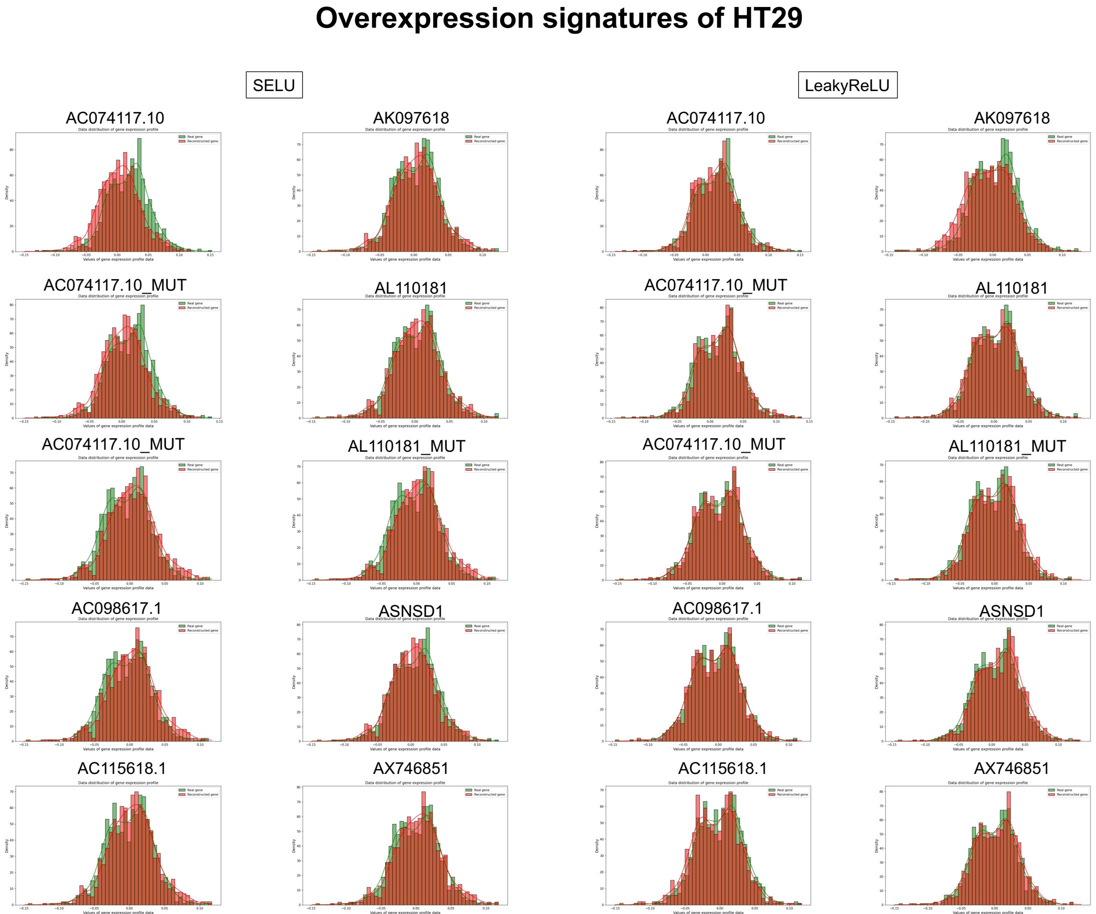

Supplementary Fig. S29: Distribution of original and reconstructed gene overexpression signatures of HT29.

The left 10 panels show original and reconstructed signatures using multimodal VQ-VAE with SELU activation function. The right 10 panels show original and reconstructed signatures using multimodal VQ-VAE with LeakyReLU. Horizontal and vertical axes represent gene expression scores and frequency. Green and red represent original and reconstructed signatures, respectively.

Overexpression signatures of MCF7

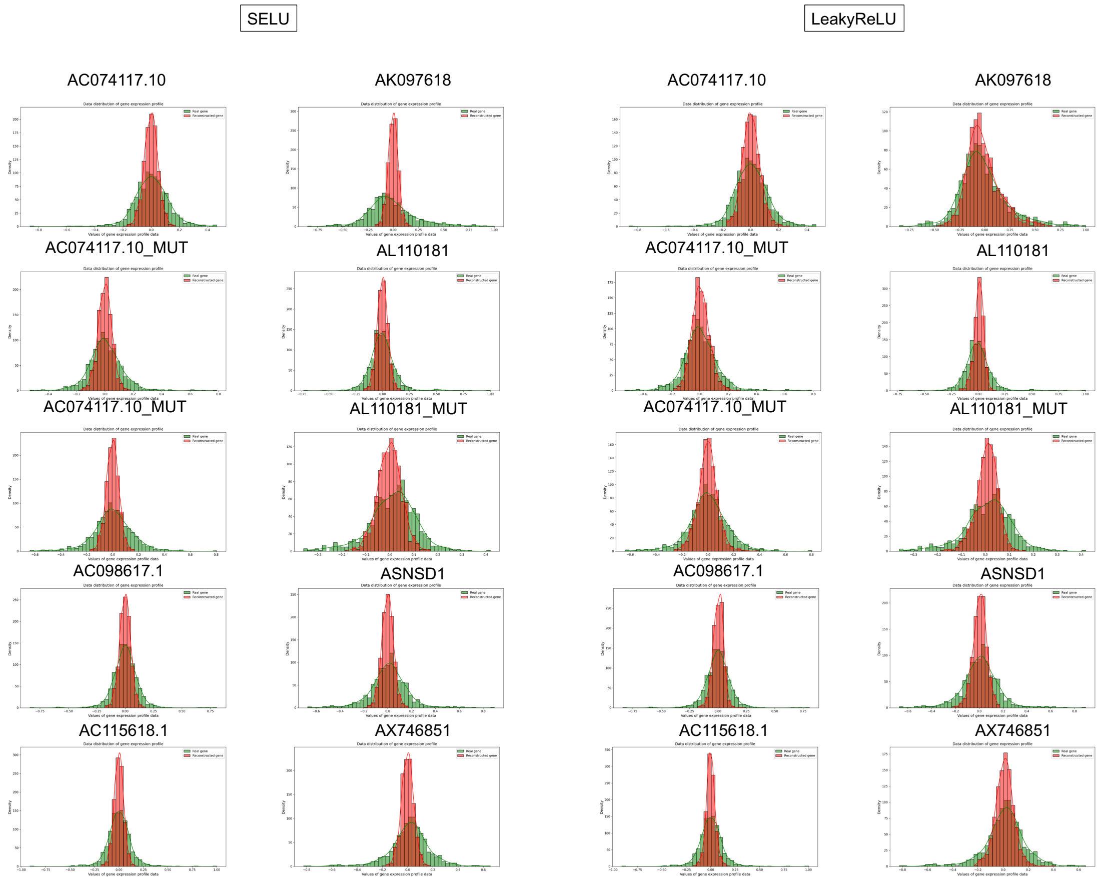

Supplementary Fig. S30: Distribution of original and reconstructed gene overexpression signatures of MCF7.

The left 10 panels show original and reconstructed signatures using multimodal VQ-VAE with SELU activation function. The right 10 panels show original and reconstructed signatures using multimodal VQ-VAE with LeakyReLU. Horizontal and vertical axes represent gene expression scores and frequency. Green and red represent original and reconstructed signatures, respectively.

Overexpression signatures of PC3

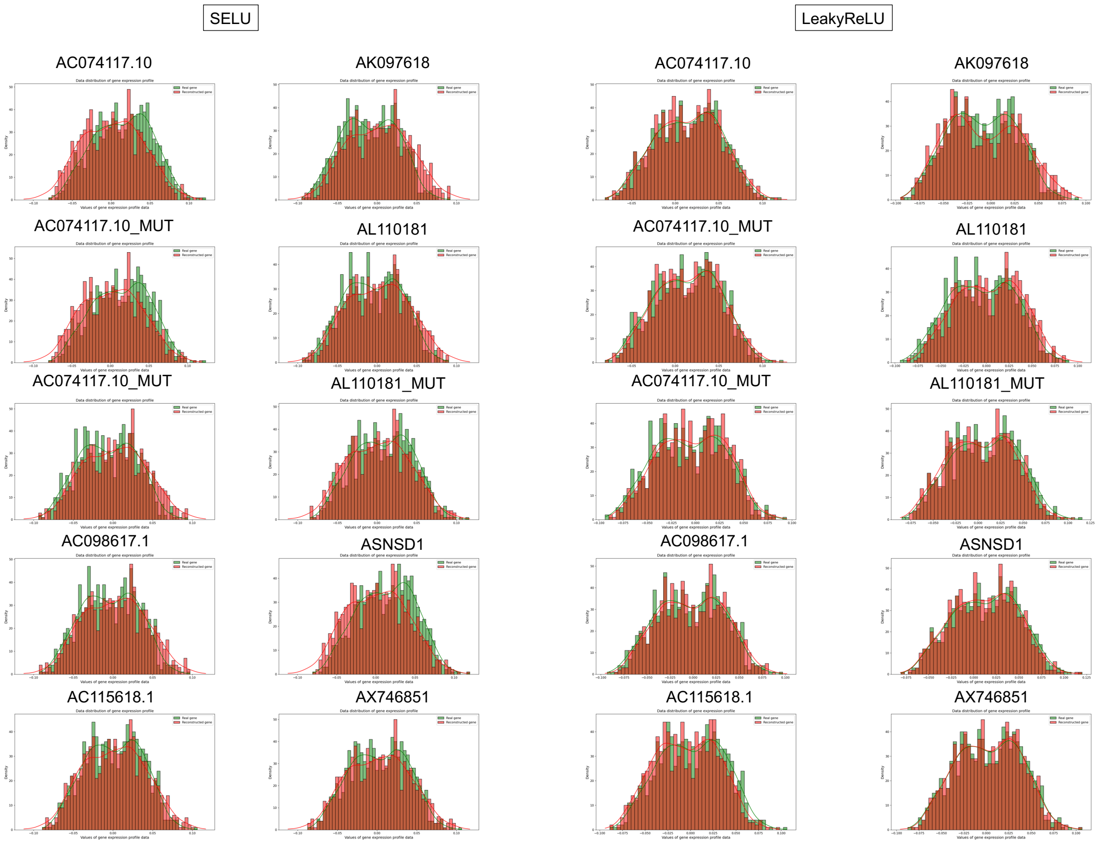

Supplementary Fig. S31: Distribution of original and reconstructed gene overexpression signatures of PC3.

The left 10 panels show original and reconstructed signatures using multimodal VQ-VAE with SELU activation function. The right 10 panels show original and reconstructed signatures using multimodal VQ-VAE with LeakyReLU. Horizontal and vertical axes represent gene expression scores and frequency. Green and red represent original and reconstructed signatures, respectively.

Overexpression signatures of VCAP

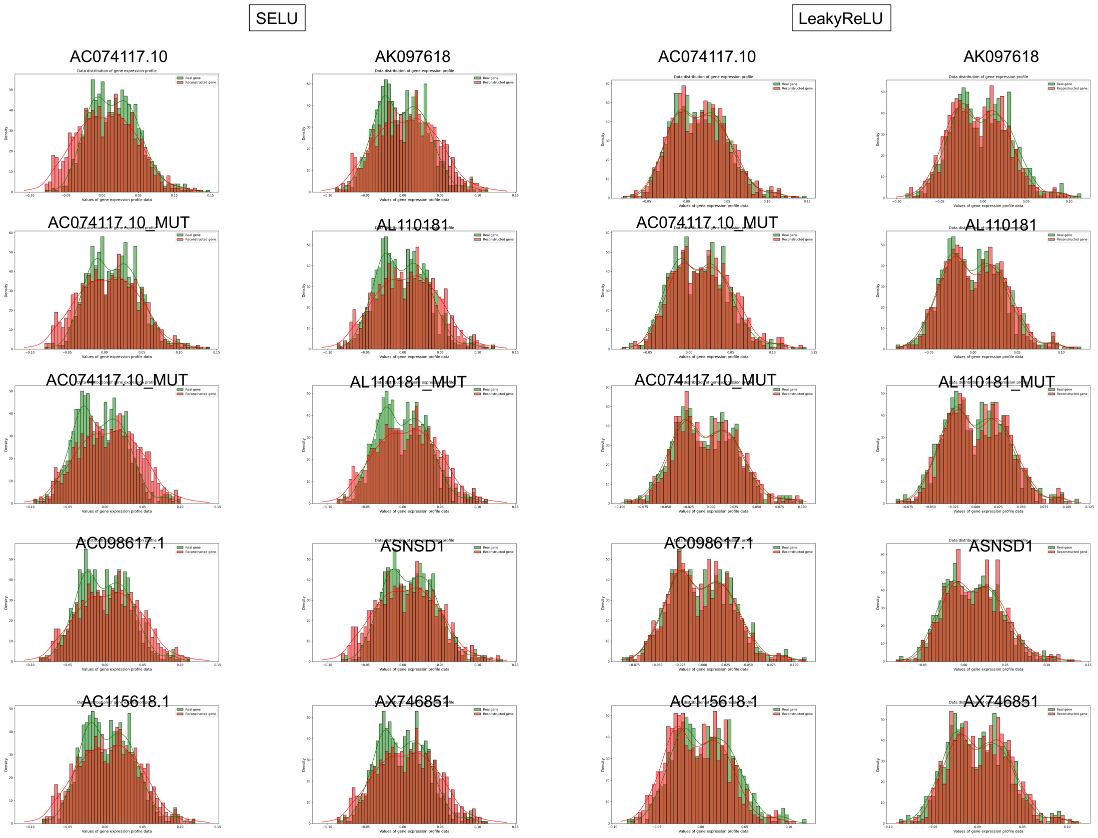

Supplementary Fig. S32: Distribution of original and reconstructed gene overexpression signatures of VCAP.

The left 10 panels show original and reconstructed signatures using multimodal VQ-VAE with SELU activation function. The right 10 panels show original and reconstructed signatures using multimodal VQ-VAE with LeakyReLU. Horizontal and vertical axes represent gene expression scores and frequency. Green and red represent original and reconstructed signatures, respectively.

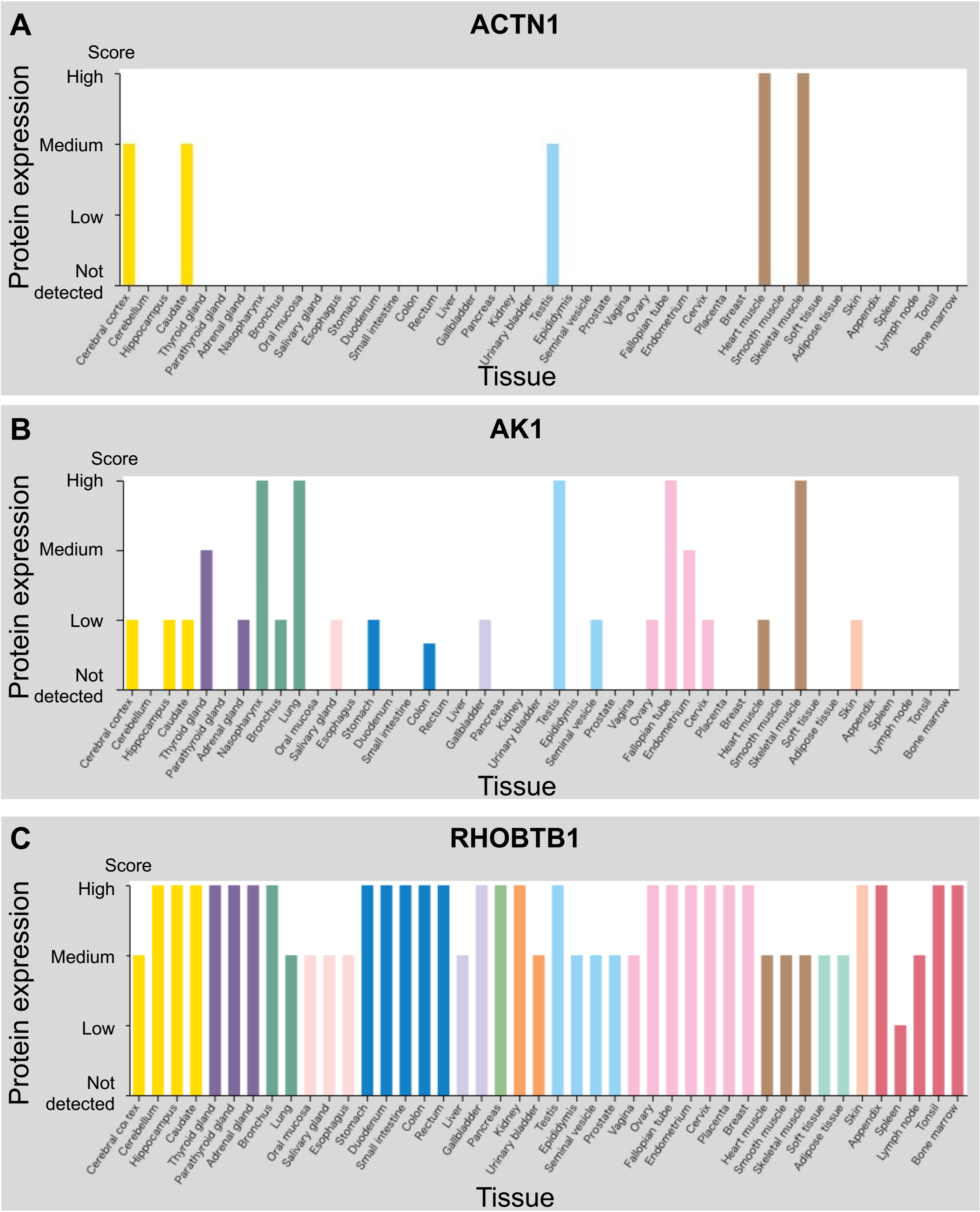

Supplementary Fig. S33: Protein expressions of activatory targets predicted for Huntington's diseases (HD).

(A–C) Protein expression levels of ACTN2, RHOBTB1 and AK1, activatory targets predicted for HD (shown in Fig. 4C–E), were assessed at four levels—"Not detected," "Low," "Medium," and "High"—across 44 tissues using The Human Protein Atlas [https://www.proteinatlas.org/]. Colors represents tissue types.

Knockdown signatures

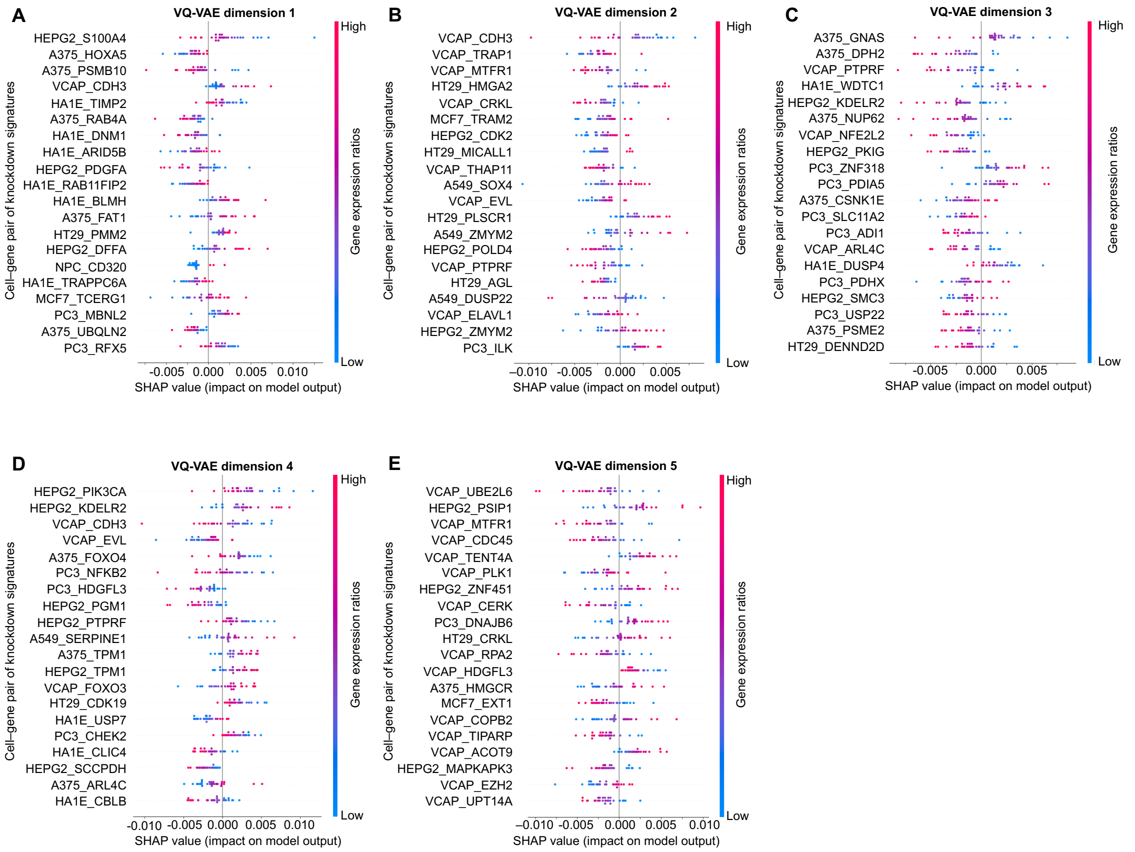

Supplementary Fig. S34: Biological interpretation of protein knockdown VQ signatures.

The contribution of input genes to each latent variable forming protein multimodal VQ signatures was calculated using SHapley Additive exPlanations (SHAP). The vertical axis corresponds to the cell type–gene pairs that constitute to protein knockdown profiles, where only pairs with large SHAP values shown. The horizontal axis corresponds to the SHAP values. The colors represent the gene expression ratios of the knockdown profiles.

Overexpression signatures

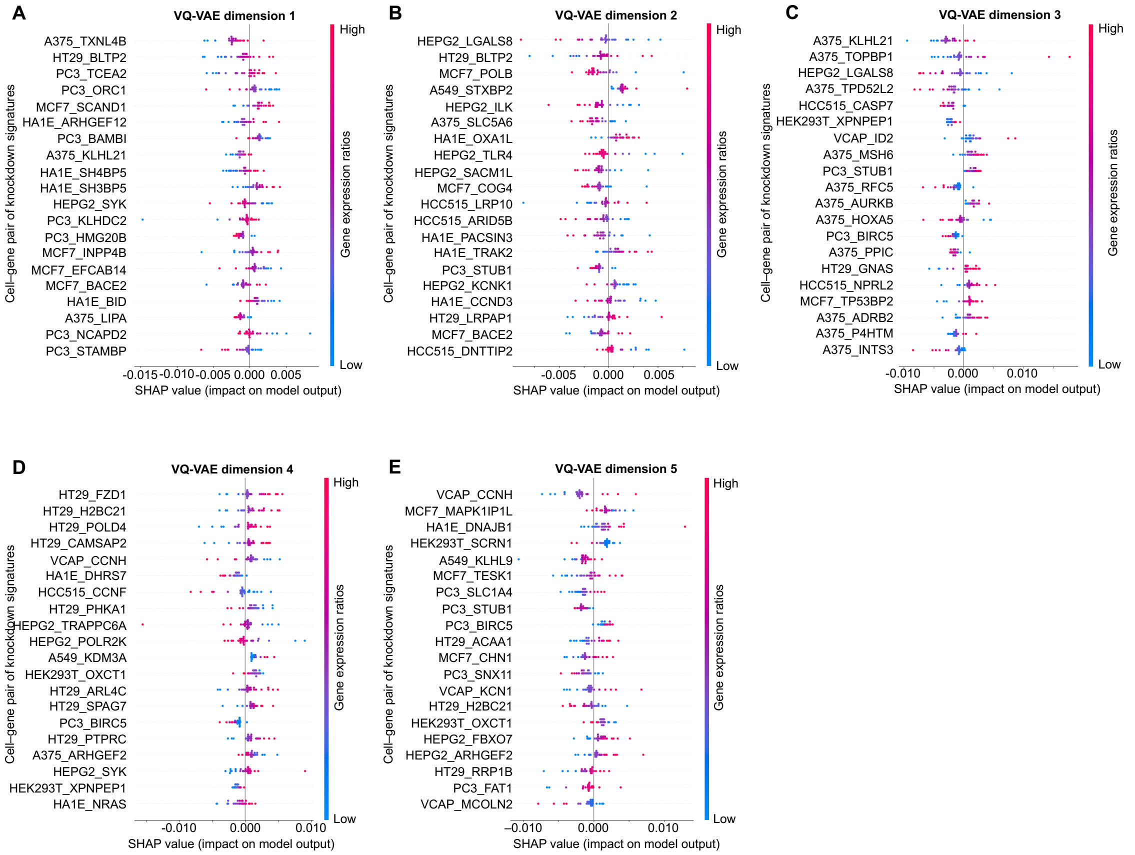

Supplementary Fig. S35: Biological interpretation of protein overexpression VQ signatures. The contribution of input genes to each latent variable forming protein multimodal VQ signatures was calculated using SHapley Additive exPlanations (SHAP). The vertical axis corresponds to the cell type–gene pairs that constitute to protein overexpression profiles, where only pairs with large SHAP values shown. The horizontal axis corresponds to the SHAP values. The colors represent the gene expression ratios of the overexpression profiles.

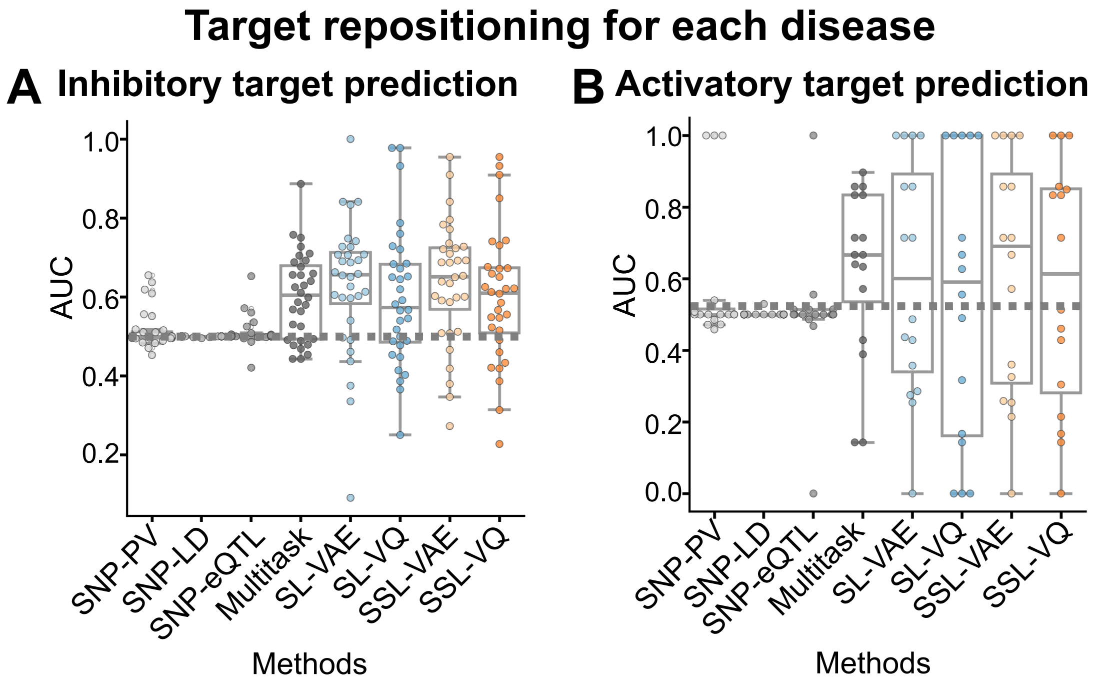

Supplementary Fig. S36: Performance evaluation of target repositioning for each disease.

(A) Comparison of proposed (SSL-VQ, SSL-VAE, SL-VQ and SL-VAE) and baseline (SNP-PV, SNP-LD, SNP-eQTL and Multitask) methods for predicting inhibitory targets involving 33 diseases and 225 proteins. Boxplots represent AUC score distributions for diseases. (B) As described in (A), but for activatory target predictions involving 16 diseases and 37 proteins.

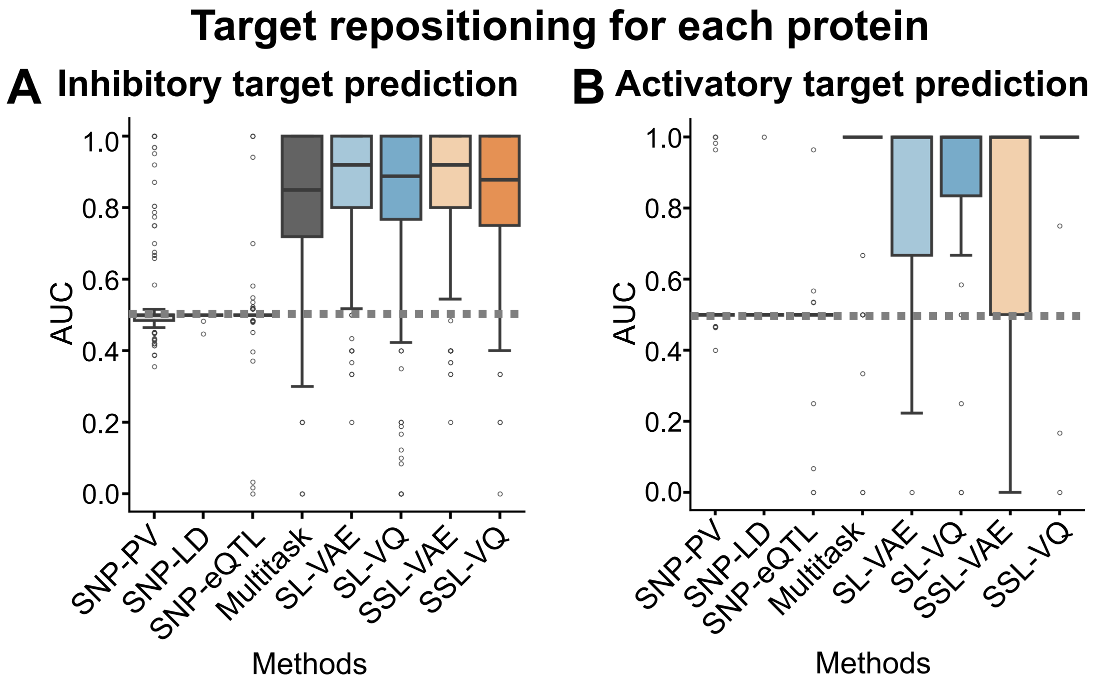

Supplementary Fig. S37: Performance evaluation of target repositioning for each protein. (A) Comparison of proposed (SSL-VQ, SSL-VAE, SL-VQ and SL-VAE) and baseline (SNP-PV, SNP-LD, SNP-eQTL and Multitask) methods for predicting inhibitory targets involving 33 diseases and 225 proteins. Boxplots represent AUC score distributions for proteins. (B) As described in (A), but for activatory target predictions involving 16 diseases and 37 proteins.

Supplementary Tables

Supplementary Table S1: Cell line list of target gene perturbation profiles.

The gene knockdown profiles consist of data from 17 cell lines and the gene overexpression profiles from 21 cell lines. These cell lines are listed alphabetical order.

Type of gene perturbation	Cell lines
Gene knockdown	A375, A549, ASC, HA1E, HCC515, HEK293T, HEKTE, HEPG2, HT29, MCF7, NCIH716, NPC, PC3, SHSY5Y, SKL, SW480, VCAP
Gene overexpression	293T, A375, A549, AALE, H1299, HA1E, HCC515, HEK293, HEK293T, HELA, HEPG2, HT29, HUH7, LHSAR, LNCAP, MCF7, PC3, RWPE, SALE, U2OS, VCAP

Supplementary Table S2: Knockdown and overexpressed gene list.

In total, 4,345 knockdown genes and 4,040 overexpression genes were used to predict new therapeutic targets for diseases. These proteins are listed in alphabetical order.

Perturbation type	Perturbed genes
Knockdown	61E3.4, A2M, AAK1, AARS, AATF, AATK, ABAT, ABCA1, ABCA3, ABCA5, ABCB1, ABCB4, ABCB5, ABCB6, ABCB7, ABCC1, ABCC10, ABCC2, ABCC3, ABCC4, ABCC5, ABCD3, ABCF1, ABCF2, ABCF3, ABCG2, ABCG5, ABCG8, ABHD2, ABHD4, ABHD6, ABL1, ABL2, ACAA1, ACACA, ACACB, ACAD8, ACADM, ACADS, ACADSB, ACAT1, ACAT2, ACBD3, ACD, ACLY, ACO2, ACOT9, ACSL1, ACSL3, ACSL4, ACSL6, ACTB, ACTN1, ACTR2, ACTR3, ACVR1, ACVR1B, ACVR1C, ACVR2A, ACVR2B, ACVRL1, ACY1, ADA, ADAM10, ADAM15, ADAM17, ADAR, ADAT1, ADC, ADCK1, ADCK2, ADCK3, ADCK4, ADCK5, ADCY3, ADCY5, ADCY9, ADH5, ADH6, ADI1, ADK, ADNP2, ADO, ADORA2A, ADORA2B, ADPGK, ADRA2A, ADRA2C, ADRB2, ADRBK1, ADRBK2, ADSL, ADSS, AES, AFF1, AFF4, AFG3L2, AFP, AGK, AGL, AGPAT1, AGPAT2, AGT, AGTR1, AHCY, AHR, AHRR, AIFM1, AIM2, AIMP2, AIP, AIRE, AK1, AK2, AK3, AK4, AK5, AK7, AK8, AKAP1, AKAP11, AKAP13, AKAP17A, AKAP8, AKAP8L, AKAP9, AKR1A1, AKR1B1, AKR1C1, AKR1C2, AKR1C3, AKR7A2, AKT1, AKT1S1, AKT2, AKT3, AKTIP, ALAD, ALAS1, ALAS2, ALB, ALDH18A1, ALDH1A1, ALDH1A3, ALDH1B1, ALDH2, ALDH3A1, ALDH3A2, ALDH3B1, ALDH3B2, ALDH4A1, ALDH5A1, ALDH6A1, ALDH7A1, ALDH9A1, ALDOA, ALDOC, ALG5, ALG9, ALK, ALMS1, ALPK1, ALPK2, ALPK3, ALX1, AMBP, AMD1, AMDHD2, AMHR2, AMIGO3, AMPD2, AMT, ANAPC5, ANG, ANGPT1, ANKK1, ANKRD10, ANKRD37, ANKRD49, ANKRD55, ANKZF1, ANO1, ANO10, ANP32E, ANPEP, ANXA1, ANXA2, ANXA3, ANXA5, ANXA7, AOC3, AP1B1, AP1S2, AP2A1, AP2A2, AP2M1, AP4S1, APAF1, APBB2, APC, APEH, APEX1, APOA1, APOA2, APOA5, APOB, APOBEC3H, APOC2, APOC3, APOE, APOH, APOM, APOO, APP, APPBP2, APRT, AQP12A, AR, ARAF, ARAP1, ARF1, ARF4, ARF6, ARFIP2, ARFRP1, ARG1, ARG2, ARGLU1, ARHGAP1, ARHGAP35, ARHGAP9, ARHGDIB, ARHGEF11, ARHGEF12, ARHGEF2, ARHGEF5, ARHGEF6, ARHGEF7, ARID3A, ARID4B, ARID5B, ARL1, ARL3, ARL4C, ARNT, ARNT2, ARNTL, ARNTL2, ARPC1A, ARPC1B, ARPC2, ARPC3, ARPC4, ARPC5, ARPP19, ARRB1, ARSG, ARX, ASAH1, ASAP2, ASCC3, ASCL4, ASF1A, ASF1B, ASGR2, ASH2L, ASL, ASMTL, ASNA1, ASNS, ASPH, ASPM, ASRGL1, ASS1, ATF1, ATF2, ATF3, ATF4, ATF5, ATF6, ATG12, ATG16L1, ATG16L2, ATG3, ATG4A, ATG4B, ATG4D, ATG5, ATG7, ATIC, ATM, ATMIN, ATOX1, ATP11B, ATP1A1, ATP1A3, ATP1B1, ATP2A2, ATP2C1, ATP5A1, ATP5B, ATP5C1, ATP5D, ATP5F1, ATP5G1, ATP5H, ATP5L, ATP5O, ATP5S, ATP6AP1, ATP6V0A1, ATP6V0B, ATP6V0C, ATP6V1A, ATP6V1D, ATP6V1F, ATP7A, ATR, ATRX, ATXN1, ATXN3, AURKA, AURKAIP1, AURKB, AURKC, AXIN1, AXIN2, AXL, AZGP1, AZI2, B2M, B3GAT3, B3GNT1, B4GALNT1, B4GALT1, B4GALT3, B4GALT4, BACE1, BACE2, BACH1, BACH2, BAD, BAG1, BAG3, BAG6, BAK1, BAMBI, BARX1, BATF, BATF3, BAX, BAZ1B, BAZ2B, BBC3, BBS9, BCAR1, BCAT1, BCAT2, BCCIP, BCKDHA, BCKDK, BCL10, BCL2, BCL2A1, BCL2L1, BCL2L11, BCL2L13, BCL2L2, BCL3, BCL6, BCL6B, BCL7B, BCL9, BCL9L, BCLAF1, BCR, BDH1, BDKRB2, BDNF, BECN1, BHLHE40, BHMT, BHMT2, BID, BIK, BIRC2, BIRC3, BIRC5, BIRC6, BLCAP, BLK, BLM, BLMH, BLNK, BLOC1S1, BLVRA, BLVRB, BLZF1, BMI1, BMP2, BMP2K, BMP2KL, BMP4, BMPR1A, BMPR1B, BMPR2, BMX, BNIP1, BNIP2, BNIP3, BNIP3L, BNIPL, BOLA1, BPHL, BRAF, BRAP, BRCA1, BRCA2, BRD1, BRD2, BRD3, BRD4, BRD8, BRDT, BRF2, BRP44, BRPF1, BRPF3, BRSK1, BRSK2, BTBD3, BTG1, BTG2, BTG3, BTK, BTRC, BUB1, BUB1B, BUB3, BUD13, BUD31, BZW2, C10ORF26, C12ORF51, C14ORF181, C15ORF42, C16ORF5, C16ORF80, C17ORF75, C19ORF10, C19ORF35, C19ORF6, C1ORF106, C1ORF9, C1QBP, C1QTNF6, C1S, C2, C21ORF7, C2CD2, C2CD2L, C2ORF3, C3, C5, C5ORF62, C9ORF95, C9ORF96, CA12, CA2, CAB39, CABIN1, CACNA1C, CACNA1D, CAD, CALCOCO2, CALM1, CALM2, CALM3, CALML6, CALR, CALU, CAMK1, CAMK1D, CAMK1G, CAMK2A, CAMK2B, CAMK2D, CAMK2G, CAMK4, CAMKK1, CAMKK2, CAMKV, CAMLG, CAMSAP2, CAND1, CAND2, CANT1, CANX, CAPG, CAPN1, CAPNS1, CAPNS2, CAPSL, CARD10, CARD11, CARD9, CARS, CASC3, CASD1, CASK, CASP10, CASP14, CASP2, CASP3, CASP4, CASP6, CASP7, CASP8, CASP9, CAST, CAT, CAV1, CBFA2T3, CBFB, CBLB, CBLC, CBR1, CBR3, CBS, CBX1, CBX3, CBX4, CBX6, CBX7, CCBL1, CCDC85B, CCDC86, CCDC90A, CCDC92, CCL2, CCL20, CCL4, CCNA1, CCNA2, CCNB1, CCNB2, CCNC, CCND1, CCND2, CCND3, CCNE1, CCNE2, CCNF, CCNG1, CCNG2, CCNH, CCNL1, CCP110, CCRL2, CCRN4L, CCT7, CCT8, CD14, CD19, CD1C, CD2, CD24, CD2BP2, CD320, CD3D, CD40, CD44, CD46, CD55, CD58, CD59, CD70, CD79B, CD81, CD83, CD97, CD99, CDA, CDC123, CDC20, CDC25A, CDC25B, CDC25C, CDC37, CDC42, CDC42BPA, CDC42BPB, CDC42BPG, CDC42SE1, CDC45, CDC7, CDCA4, CDCA7L, CDCA8, CDH1, CDH11, CDH3, CDIPT, CDK1, CDK10, CDK11A, CDK11B, CDK12, CDK14, CDK15, CDK16, CDK17, CDK18, CDK19, CDK2, CDK20, CDK3, CDK4, CDK5, CDK5R1, CDK5R2, CDK6, CDK7, CDK8, CDK9, CDKAL1, CDKL1, CDKL2, CDKL3, CDKL4, CDKL5, CDKN1A, CDKN1B, CDKN1C, CDKN2A, CDKN2B, CDKN2C, CDO1, CDT1, CDX1, CDX2, CEACAM1, CEACAM6, CEBPA, CEBPB, CEBPD, CEBPG, CEBPZ, CELSR2, CELSR3, CENPE, CEP55, CEP57, CEP72, CEPT1, CERK, CERKL, CERS2, CERS3, CERS4, CES1, CES2, CETN2, CETN3, CFB, CFD, CFL1, CFLAR, CFTR, CGRRF1, CHAC1, CHAF1B, CHCHD7, CHD1, CHD4, CHD8, CHEK1, CHEK2, CHERP, CHIC2, CHKA, CHKB, CHMP2A, CHMP4A, CHMP6, CHN1, CHRAC1, CHRM1, CHRM3, CHUK, CIAPIN1, CIB1, CIB4, CIITA, CIR1, CIRBP, CISD1, CISH, CIT, CKB, CKM, CKMT1B, CKMT2, CKS1B, CKS2, CLASRP, CLCN3, CLCN5, CLEC16A, CLIC4, CLK1, CLK2, CLK3, CLK4, CLN3, CLOCK, CLPB, CLPP, CLPTM1, CLPX, CLSTN1, CLTA, CLTB, CLTC, CMAS, CMPK1, CNDP2, CNNM2, CNOT3, CNOT4, CNOT7, CNOT8, CNPY3, COASY, COBL, COG2, COG4, COG7, COL13A1, COL4A2, COL4A3BP, COL4A5, COMT, COPA, COPB2, COPS2, COPS5, COPS7A, COPZ1, COQ3, CORO1A, COX4I1, COX5A, COX5B, COX6B2, COX6C, COX7B, CP, CPD, CPE, CPNE3, CPS1, CPSF4, CPT1A, CPT2, CRABP1, CRABP2, CRADD, CRAT, CRCP, CREB1, CREB3, CREB3L1, CREB3L2, CREB3L4, CREBBP, CREBL2, CREG1, CRELD2, CREM, CRK, CRKL, CROT, CRTAP, CRY1, CRYAA, CRYZ, CS, CSAD, CSDA, CSF1R, CSF3, CSK, CSMD1, CSNK1A1, CSNK1A1L, CSNK1D, CSNK1E, CSNK1G1,

	CSNK1G2, CSNK1G3, CSNK2A1, CSNK2A2, CSNK2B, CSRP1, CTBP1, CTBP2, CTCF, CTCFL, CTDSP1, CTGF, CTH, CTLA4, CTNNA1, CTNNAL1, CTNNB1, CTNNBIP1, CTNND1, CTNS, CTPS, CTRB1, CTRB2, CTSB, CTSD, CTSH, CTSK, CTSL1, CTSL2, CTTN, CUL2, CXADR, CXCL1, CXCL12, CXCL2, CXCR2, CXCR4, CXCR7, CXXC4, CYB561, CYB5A, CYB5B, CYB5R3, CYC1, CYCS, CYLD, CYP1A1, CYP1B1, CYP20A1, CYP24A1, CYP27B1, CYP3A5, CYP51A1, CYTH1, DACH2, DAG1, DAK, DAP, DAPK1, DAPK2, DAPK3, DARS, DAXX, DBI, DCAKD, DCK, DCLK1, DCLK2, DCLK3, DCLRE1B, DCP1A, DCPS, DCTD, DCTN6, DCUN1D4, DCXR, DDAH1, DDAH2, DDB1, DDB2, DDC, DDIT3, DDIT4, DDOST, DDR1, DDR2, DDX10, DDX39B, DDX42, DDX49, DDX5, DECR1, DEK, DENND1B, DENND2D, DEPTOR, DERA, DERL1, DFFA, DFFB, DFNB31, DGCR14, DGKA, DGKB, DGKD, DGKE, DGKG, DGKH, DGKK, DGKQ, DGKZ, DGUOK, DHCR24, DHCR7, DHDDS, DHFR, DHH, DHPS, DHRS2, DHRS3, DHRS4, DHRS7, DHTKD1, DHX16, DHX29, DHX8, DIABLO, DICER1, DIDO1, DIS3, DISC1, DIXDC1, DKC1, DKK1, DLAT, DLC1, DLD, DLG1, DLG2, DLG3, DLG5, DLGAP5, DLL1, DLX2, DLX3, DMD, DMPK, DMRTB1, DMTF1, DNA2, DNAJA3, DNAJB1, DNAJB12, DNAJB2, DNAJB6, DNAJB9, DNAJC1, DNAJC12, DNAJC15, DNAJC17, DNAJC6, DNM1, DNM1L, DNMT1, DNMT3A, DNMT3L, DNPEP, DNTTIP2, DOK4, DOT1L, DPF1, DPH2, DPP4, DPY30, DPYD, DR1, DRAP1, DROSHA, DRP2, DSE, DSG2, DSTYK, DTNA, DTX1, DTX2, DTX3L, DTYMK, DUSP1, DUSP11, DUSP14, DUSP2, DUSP22, DUSP3, DUSP6, DUSP7, DUSP9, DUT, DVL1, DVL2, DVL3, DYNLL1, DYNLL2, DYNLT3, DYRK1A, DYRK1B, DYRK2, DYRK3, DYRK4, E2F1, E2F3, E2F4, E2F5, E2F6, E2F8, EAPP, EBF1, EBNA1BP2, EBP, ECD, ECH1, ECHS1, ECI1, ECSIT, EDEM1, EDN1, EDNRB, EED, EEF1D, EEF2K, EFCAB2, EFNB2, EFNB3, EGF, EGFR, EGLN1, EGLN2, EGR1, EGR2, EGR3, EHF, EHHADH, EHMT2, EIF1B, EIF2A, EIF2AK1, EIF2AK2, EIF2AK3, EIF2AK4, EIF2B2, EIF2B3, EIF2B5, EIF2C1, EIF2C2, EIF2C3, EIF2S2, EIF3H, EIF3J, EIF4A2, EIF4B, EIF4E, EIF4EBP1, EIF4G1, EIF5, EIF5A, EIF6, ELAC2, ELAVL1, ELF1, ELF3, ELF4, ELK1, ELK3, ELL3, ELOVL6, ELTD1, EMD, EML3, EMR1, ENDOG, ENO1, ENOPH1, ENOSF1, ENPP1, ENTPD6, EP300, EP400, EPAS1, EPB41L2, EPB41L4B, EPC1, EPCAM, EPDR1, EPHA1, EPHA10, EPHA2, EPHA3, EPHA4, EPHA5, EPHA6, EPHA7, EPHA8, EPHB1, EPHB2, EPHB3, EPHB4, EPHB6, EPHX1, EPHX2, EPN2, EPO, EPOR, EPRS, EPS8, ERAP1, ERAP2, ERBB2, ERBB2IP, ERBB3, ERBB4, ERCC1, ERCC2, ERCC3, ERCC4, ERCC5, ERCC6L, ERF, ERG, ERGIC1, ERGIC2, ERH, ERLIN1, ERN1, ERN2, ERO1L, ERRFI1, ESD, ESPL1, ESR1, ESR2, ESRRA, ESYT1, ETFA, ETFB, ETNK1, ETNK2, ETS1, ETS2, ETV1, ETV4, ETV5, ETV6, EVL, EWSR1, EXO1, EXOC2, EXOC3, EXOC6, EXOSC10, EXOSC4, EXOSC8, EXT1, EXT2, EYA1, EZH1, EZH2, F10, F11, F12, F2, F2R, F2RL1, F3, F5, F7, FAAH, FABP1, FABP4, FABP5, FABP6, FADD, FADS1, FADS2, FADS3, FAF1, FAH, FAIM, FAM102A, FAM104A, FAM114A2, FAM120A, FAM134C, FAM20B, FAM3C, FAM57A, FAM63A, FAM69A, FANCA, FANCF, FANCG, FANCL, FAS, FASLG, FASN, FASTK, FASTKD1, FASTKD2, FASTKD3, FASTKD5, FAT1, FAT4, FAU, FBP1, FBRS, FBXL12, FBXL19, FBXL20, FBXL4, FBXO11, FBXO21, FBXO5, FBXO7, FBXW11, FCGR2A, FCHO1, FDFT1, FDPS, FDX1, FDX1L, FDXR, FECH, FEN1, FER, FERD3L, FERMT1, FES, FEZ1, FEZ2, FFAR1, FGA, FGB, FGF10, FGF2, FGF9, FGFR1, FGFR1OP, FGFR2, FGFR3, FGFR4, FGFRL1, FGG, FGGY, FGR, FH, FHL2, FIBP, FIGF, FIS1, FKBP14, FKBP1A, FKBP3, FKBP4, FKBP5, FKBP8, FLI1, FLJ40852, FLT1, FLT3, FLT4, FMR1, FN1, FN3K, FN3KRP, FOLR1, FOS, FOSL1, FOSL2, FOXA1, FOXA2, FOXA3, FOXD4L3, FOXF2, FOXJ2, FOXJ3, FOXN3, FOXO1, FOXO3, FOXO4, FOXP3, FOXP4, FOXQ1, FOXR1, FPGS, FRAT1, FRAT2, FRK, FRS2, FSD1, FST, FTCD, FTH1, FTL, FTSJ1, FUK, FURIN, FUS, FUT1, FUT2, FXN, FXYD2, FYN, FZD1, FZD2, FZD3, FZD4, FZD5, FZD6, FZD7, FZD8, G2E3, G3BP1, G3BP2, G6PC, G6PD, GAA, GABARAPL1, GABBR1, GABBR2, GABPB1, GABPB2, GABRA1, GABRA5, GABRB3, GABRG1, GABRP, GADD45A, GADD45B, GAK, GALC, GALE, GALK1, GALK2, GALNS, GALNT1, GALT, GAMT, GAPDH, GARS, GART, GAS6, GAS7, GATA1, GATA2, GATA3, GATA6, GATAD1, GATAD2A, GATAD2B, GBA, GBGT1, GBP2, GCA, GCAT, GCDH, GCH1, GCK, GCLC, GCLM, GDI1, GDPD5, GEMIN2, GFOD1, GFOD2, GFPT1, GFPT2, GFRA1, GGA2, GGCT, GGCX, GGH, GGPS1, GGT1, GHR, GIPC1, GIT1, GIT2, GJA1, GK, GK2, GK5, GLA, GLDC, GLI1, GLI3, GLIS3, GLOD4, GLRX, GLRX2, GLS, GLTSCR1, GLUD1, GLUD2, GLUL, GLYCTK, GM2A, GMDS, GMEB1, GMEB2, GMNN, GMPR2, GMPS, GNA11, GNA12, GNA13, GNA15, GNAI1, GNAI2, GNAI3, GNAL, GNAQ, GNAS, GNAZ, GNB1, GNB1L, GNB2, GNB5, GNE, GNG4, GNG5, GNG8, GNGT2, GNPDA1, GOLGA5, GOLIM4, GOLT1B, GOT1, GOT2, GPATCH8, GPBAR1, GPC1, GPER, GPI, GPR101, GPR107, GPR110, GPR111, GPR112, GPR113, GPR114, GPR115, GPR119, GPR123, GPR125, GPR126, GPR128, GPR132, GPR133, GPR137, GPR139, GPR141, GPR142, GPR146, GPR148, GPR150, GPR151, GPR152, GPR153, GPR156, GPR158, GPR160, GPR172A, GPR174, GPR176, GPR179, GPR19, GPR26, GPR31, GPR34, GPR35, GPR37, GPR39, GPR4, GPR55, GPR56, GPR61, GPR62, GPR64, GPR65, GPR78, GPR82, GPR83, GPR84, GPR87, GPRC5A, GPRC5B, GPRC5C, GPRC6A, GPSM1, GPSM2, GPX1, GPX2, GPX3, GPX4, GPX7, GRB10, GRB2, GRB7, GREB1, GRHL3, GRHPR, GRIN3A, GRK1, GRK4, GRK5, GRK6, GRK7, GRN, GRPR, GRWD1, GSC, GSDMB, GSG2, GSK3A, GSK3B, GSR, GSS, GSTM1, GSTM2, GSTP1, GSTT1, GSTZ1, GTDC1, GTF2A1, GTF2A2, GTF2B, GTF2E2, GTF2F2, GTF2H1, GTF2H2, GTF2H3, GTF2I, GTF3C2, GTPBP8, GUCY2C, GUCY2D, GUCY2F, GUK1, GUSB, GYG1, GYS1, H1FOO, H2AFV, H2AFX, HADH, HADHA, HADHB, HAGH, HAL, HARS, HAT1, HAVCR1, HAX1, HBE1, HBP1, HCAR1, HCFC1, HCFC2, HCK, HCN3, HDAC1, HDAC10, HDAC11, HDAC2, HDAC3, HDAC4, HDAC5, HDAC6, HDAC7, HDAC8, HDAC9, HDGFRP3, HDHD1, HEATR1, HEBP1, HECW2, HERC6, HERPUD1, HES1, HES5, HEXB, HEXIM1, HEY1, HFE, HGS, HHEX, HIBADH, HIC2, HIF1A, HIF1AN, HIGD2A, HINT1, HIPK1, HIPK2, HIPK3, HIPK4, HIST1H1B, HIST1H1C, HIST1H1E, HIST1H2AC, HIST1H2AL, HIST1H2BD, HIST1H2BK, HIST1H3A, HIST1H3B, HIST2H2BE, HK1, HK2, HK3, HKDC1, HLA-A, HLA-B, HLA-DMA, HLA-DMB, HLA-DPA1, HLA-DPB1, HLA-DQA1, HLA-DQB1, HLA-DRA, HLA-DRB1, HLA-DRB3, HLA-DRB4, HLA-DRB5, HLF, HMBOX1, HMG20B, HMGA1, HMGA2, HMGB1, HMGB2, HMGB3, HMGB4, HMGCL, HMGCR, HMGCS1, HMGCS2, HMGN3, HMGN4, HMGN5, HMMR, HMOX1, HMOX2, HN1L, HNF1A, HNF1B, HNF4A, HNF4G, HNMT, HOMER2, HOMEZ, HOOK2, HOPX, HORMAD2, HOXA1, HOXA10, HOXA2, HOXA5, HOXA6, HOXA9, HOXB13, HOXB2, HOXB4, HOXB5, HOXB7, HOXC10, HOXC4, HOXC9, HPD, HPGD, HPN, HPRT1, HRAS, HRH1, HRSP12, HS2ST1, HS3ST3A1, HSBP1, HSD17B10, HSD17B11, HSD17B2, HSD17B4, HSD17B7, HSD17B8, HSF1, HSF2, HSF5, HSP90AA1, HSP90AB1, HSP90B1, HSPA14, HSPA1A, HSPA1B, HSPA2, HSPA4, HSPA5, HSPA8, HSPA9, HSPB1, HSPB8, HSPD1, HSPE1, HSPG2, HTATIP2, HTATSF1, HTR2C, HTRA1, HUNK, HUS1, HYAL1, HYAL2, HYOU1, IARS, IARS2, ICAM1, ICAM3, ICK, ICMT, ID1, ID2, ID3, ID4, IDE, IDH1, IDH2, IDH3A, IDH3B, IDH3G, IER3, IFI16, IFI30, IFIH1, IFIT5, IFNAR2, IFNB1, IFNG, IFNGR1, IFNGR2, IFRD2, IGBP1, IGF1, IGF1R, IGF2, IGF2BP1, IGF2BP2, IGF2BP3, IGF2R, IGFBP2, IGFBP3, IGFBP4, IGFBP5, IGFBP6, IGFBP7, IGFN1, IGHMBP2, IGSF8, IKBKAP, IKBKB, IKBKE, IKBKG, IKZF1, IKZF5, IL10, IL10RB, IL11, IL11RA, IL12A, IL12B, IL13, IL13RA1, IL13RA2, IL15, IL15RA, IL18, IL18RAP, IL1B, IL1R1, IL1R2, IL1RAP, IL2, IL20, IL23R, IL27, IL2RA, IL2RB, IL4, IL4R, IL5, IL6, IL6R, IL6ST, IL7R, IL8, ILF2, ILF3, ILK, IMPA1, IMPA2, IMPDH1, IMPDH2, ING1, ING2, ING3, INHBE, INPP1, INPP4B, INPP5D, INPPL1, INS, INSIG1, INSR, INSRR, INTS12, INTS3, IP6K1, IP6K2, IP6K3, IPMK, IPO13, IPO4, IPPK, IQGAP1, IRAK1, IRAK2, IRAK3, IRAK4, IRF1, IRF2, IRF3, IRF4, IRF5, IRF6, IRF7, IRF9, IRGC, IRGM, IRS1, IRS2, ISG20, ISL1, ISOC1, ISX, ITCH, ITFG1, ITGA1, ITGA2, ITGA3, ITGA4, ITGAE, ITGAV, ITGB1, ITGB1BP1, ITGB1BP3, ITGB2, ITGB3, ITGB4, ITGB5, ITK, ITPK1, ITPKA, ITPKB, ITPKC, ITPR1, ITPR2, ITPR3, ITSN1, JAG1, JAK1, JAK2, JAK3, JAZF1, JMJD6, JMJD7-PLA2G4B, JOSD1, JUN, JUNB, KARS, KAT2B, KAT5, KAT6A, KAT6B, KAT7, KAZALD1, KBTBD2, KCNA2, KCNG1, KCNJ11, KCNK1, KCNK15,

	KCNK2, KCNMA1, KCNN4, KCNQ1, KCTD2, KCTD5, KDELR2, KDELR3, KDM1A, KDM3A, KDM4A, KDM4B, KDM4C, KDM5A, KDM5B, KDM6B, KDR, KEAP1, KHDRBS1, KHK, KIAA0100, KIAA0196, KIAA0317, KIAA0355, KIAA0494, KIAA0528, KIAA0753, KIAA0907, KIAA0922, KIAA1009, KIAA1033, KIAA1267, KIAA1279, KIAA1804, KIF11, KIF13B, KIF14, KIF20A, KIF21B, KIF2C, KIF5B, KIF5C, KIF9, KIN, KISS1R, KIT, KITLG, KLF10, KLF11, KLF14, KLF2, KLF3, KLF4, KLF5, KLF6, KLF7, KLHDC2, KLHDC9, KLHL21, KLHL6, KLHL9, KLK6, KLRC1, KNDC1, KNG1, KRAS, KREMEN1, KRT19, KRT25, KRT26, KRT8, KSR1, KSR2, KTN1, KYNU, L1CAM, L3MBTL4, LACC1, LAGE3, LAMA3, LAMB1, LAMP2, LAMTOR3, LANCL1, LAP3, LARS, LARS2, LASP1, LATS1, LATS2, LBR, LCK, LCMT1, LCN2, LDHA, LDHB, LDLR, LEF1, LEPRE1, LEPREL1, LGALS1, LGALS2, LGALS3, LGALS8, LGMN, LGR4, LGR5, LGR6, LHPP, LHX2, LHX3, LHX4, LHX8, LHX9, LIAS, LIF, LIG1, LIG3, LIMK1, LIMK2, LIN28A, LIN28B, LIPA, LIPH, LIPI, LIPT1, LMAN1, LMNA, LMNB2, LMO4, LMTK2, LMTK3, LNPEP, LOC388259, LOC389906, LOC390877, LOC391295, LOC391533, LOC392226, LOC392265, LOC392347, LOC400301, LOC441047, LOC441655, LOC441708, LOC441777, LOC441971, LOC442075, LOC442313, LOC442558, LONRF2, LOXL1, LPAR1, LPAR2, LPAR3, LPAR5, LPAR6, LPGAT1, LPHN1, LPHN2, LPL, LPXN, LRGUK, LRP10, LRP4, LRP5, LRP6, LRPAP1, LRPPRC, LRRC16A, LRRC18, LRRC32, LRRC40, LRRC41, LRRC59, LRRK1, LRRK2, LRSAM1, LSM2, LSM5, LSM6, LSP1, LSR, LSS, LTA4H, LTB, LTBR, LTK, LYAR, LYN, LYPD3, LYPLA1, LYRM1, LYZ, LZIC, M6PR, MACF1, MAD2L1BP, MAF, MAFG, MAGED1, MAGI1, MAGI3, MAK, MALT1, MAML1, MAMLD1, MAN1A1, MAN2A2, MAN2B1, MAOB, MAP2K1, MAP2K2, MAP2K3, MAP2K4, MAP2K5, MAP2K6, MAP2K7, MAP3K1, MAP3K10, MAP3K11, MAP3K13, MAP3K14, MAP3K15, MAP3K2, MAP3K3, MAP3K4, MAP3K5, MAP3K6, MAP3K7, MAP3K8, MAP3K9, MAP4K1, MAP4K3, MAP4K4, MAP4K5, MAP7, MAPK1, MAPK10, MAPK11, MAPK12, MAPK13, MAPK14, MAPK15, MAPK1IP1L, MAPK3, MAPK4, MAPK6, MAPK7, MAPK8, MAPK9, MAPKAP1, MAPKAPK2, MAPKAPK3, MAPKAPK5, MARK1, MARK2, MARK2P10, MARK3, MARK4, MARS, MAS1L, MAST1, MAST2, MAST3, MAST4, MASTL, MAT1A, MAT2A, MAT2B, MATK, MATN3, MAX, MB, MBD6, MBNL1, MBNL2, MBOAT7, MBTPS1, MCCC1, MCCC2, MCHR2, MCL1, MCM2, MCM3, MCM4, MCM5, MCM6, MCM7, MCM8, MCOLN1, MCTS1, MDC1, MDH1, MDH2, MDM2, MDM4, ME1, ME2, ME3, MECOM, MECP2, MED1, MED11, MED12L, MED14, MED15, MED21, MED26, MED28, MED4, MED6, MED7, MEF2A, MEF2BNB-MEF2B, MEF2C, MEGF9, MEIS2, MELK, MEN1, MEOX2, MERTK, MESP1, MEST, MET, METAP2, METRN, METTL1, METTL14, METTL4, MEX3B, MEX3D, MFAP3, MFHAS1, MFSD10, MGAT1, MGAT4A, MGLL, MGMT, MGST2, MIB1, MICALL1, MIF, MINK1, MITF, MIXL1, MKI67IP, MKNK1, MKNK2, MKX, MLEC, MLH1, MLKL, MLL, MLL2, MLL3, MLL4, MLLT10, MLLT11, MLLT3, MLLT6, MLST8, MLXIP, MMEL1, MMP1, MMP14, MMP2, MMP7, MNAT1, MOK, MORF4L1, MORF4L2, MORN1, MORN2, MOS, MPDZ, MPHOSPH9, MPI, MPL, MPP1, MPP2, MPP3, MPP4, MPP5, MPP6, MPP7, MPZL1, MRGPRD, MRGPRF, MRGPRX1, MRGPRX2, MRGPRX3, MRGPRX4, MRPL12, MRPL18, MRPL19, MRPS16, MRPS2, MSH2, MSH5, MSH6, MSL3, MSMO1, MSRA, MSRB2, MST1, MST1R, MST4, MSX1, MSX2, MT1F, MTA1, MTA2, MTA3, MTAP, MTERFD1, MTF2, MTFR1, MTHFD1, MTHFD2, MTOR, MTR, MTRR, MTSS1, MTTP, MTX2, MUC1, MUSK, MUT, MVK, MVP, MXD3, MXD4, MXI1, MYB, MYBL2, MYC, MYCBP, MYCBP2, MYCL1, MYCL2, MYCN, MYD88, MYL6, MYL6B, MYL9, MYLIP, MYLK, MYLK2, MYLK3, MYLK4, MYNN, MYO10, MYO3B, N4BP2, NAA25, NAA50, NAB1, NAB2, NADK, NADSYN1, NAE1, NAGA, NAGK, NAGPA, NAIP, NAMPT, NANOG, NARFL, NARS, NASP, NAT1, NAT10, NBN, NBR1, NCAPD2, NCF2, NCL, NCOA2, NCOA3, NCOA4, NCOR2, NDFIP1, NDRG1, NDUFA1, NDUFA13, NDUFA2, NDUFA3, NDUFA4, NDUFA5, NDUFA6, NDUFA7, NDUFA8, NDUFA9, NDUFAB1, NDUFAF4, NDUFB1, NDUFB2, NDUFB3, NDUFB4, NDUFB5, NDUFB6, NDUFB7, NDUFB8, NDUFC1, NDUFC2, NDUFS1, NDUFS2, NDUFS3, NDUFS4, NDUFS6, NDUFS7, NDUFS8, NDUFV1, NDUFV2, NECAB3, NEDD4, NEK1, NEK10, NEK2, NEK3, NEK4, NEK5, NEK6, NEK7, NEK8, NEK9, NENF, NET1, NEU1, NEUROD1, NEUROD4, NF1, NF2, NFAT5, NFATC2, NFATC3, NFE2L1, NFE2L2, NFIB, NFIC, NFIL3, NFKB1, NFKB2, NFKBIA, NFKBIB, NFKBIE, NFKBIL1, NFRKB, NFYB, NGFR, NGRN, NID1, NIM1, NIPBL, NIPSNAP1, NISCH, NIT1, NKIRAS1, NKIRAS2, NKX2-1, NKX2-3, NKX2-5, NLK, NME1, NME2, NME3, NME4, NME5, NME6, NME7, NME9, NMI, NMNAT2, NMRAL1, NMT1, NMUR2, NNMT, NNT, NOD1, NOD2, NOL3, NOLC1, NONO, NOS3, NOSIP, NOTCH1, NOTCH2, NOTCH2NL, NOV, NOX1, NPAS3, NPBWR1, NPC1, NPDC1, NPEPL1, NPEPPS, NPFFR2, NPM1, NPR1, NPR2, NPRL2, NPSR1, NPTN, NPY1R, NQO1, NQO2, NR0B1, NR0B2, NR1D2, NR1H2, NR1H3, NR1H4, NR2C1, NR2C2, NR2F1, NR2F2, NR2F6, NR3C1, NR4A2, NR5A2, NRAS, NRBF2, NRBP1, NRBP2, NRG1, NRGN, NRIP1, NRIP3, NRK, NRP1, NRXN1, NRXN3, NSA2, NSD1, NSDHL, NSF, NSFL1C, NT5C2, NT5DC2, NT5E, NTAN1, NTPCR, NTRK1, NTRK2, NTRK3, NTSR1, NUAK1, NUAK2, NUCB2, NUCKS1, NUDCD3, NUDT14, NUDT5, NUDT6, NUDT9, NUMA1, NUMB, NUP133, NUP62, NUP85, NUP88, NUP93, NUSAP1, NVL, NXF1, O3FAR1, OAS1, OAT, OAZ1, OAZ2, OBSCN, ODC1, OGDH, OGFOD1, OGG1, OIT3, OLIG3, OPA1, OPN3, OPN4, OPN5, OR10Z1, OR52B2, OR5W2, ORC1, ORM1, ORMDL3, OSGEP, OSMR, OSR1, OSR2, OTUD3, OTUD7A, OTX1, OTX2, OVCA2, OVOL2, OXA1L, OXCT1, OXER1, OXGR1, OXSR1, OXTR, P2RX4, P2RY12, P2RY2, P2RY6, P2RY8, P4HA1, P4HA2, P4HB, P4HTM, PA2G4, PABPC1, PACSIN3, PAF1, PAFAH1B1, PAFAH1B2, PAFAH1B3, PAH, PAICS, PAK1, PAK2, PAK3, PAK4, PAK6, PAK7, PAM, PAN2, PANK1, PANK2, PANK3, PANK4, PAPD5, PAPD7, PAPOLA, PAPSS1, PAPSS2, PAQR7, PAQR8, PARG, PARK7, PARL, PARN, PARP1, PARP10, PARP2, PARP4, PASD1, PASK, PAX5, PAX6, PAX8, PBK, PBRM1, PBX1, PBX4, PBXIP1, PC, PCBD1, PCCB, PCDH15, PCDHA10, PCGF1, PCGF2, PCGF3, PCGF5, PCGF6, PCK1, PCK2, PCLO, PCM1, PCMT1, PCNA, PCSK7, PCSK9, PDAP1, PDCD1LG2, PDCD2, PDE4D, PDGFC, PDGFRA, PDGFRB, PDGFRL, PDHA1, PDHB, PDHX, PDIA2, PDIA4, PDIA5, PDIK1L, PDK1, PDK2, PDK3, PDK4, PDLIM1, PDLIM4, PDPK1, PDS5A, PDS5B, PDX1, PDXK, PDXP, PEAK1, PEBP1, PECR, PEMT, PEPD, PER1, PER2, PET112, PEX11A, PEX12, PEX13, PEX19, PFAS, PFDN2, PFKFB1, PFKFB2, PFKFB3, PFKFB4, PFKL, PFKM, PFKP, PFN2, PGAM1, PGBD1, PGD, PGF, PGK1, PGK2, PGM1, PGRMC1, PHACTR1, PHB, PHB2, PHF1, PHF10, PHF11, PHF13, PHF15, PHF16, PHF17, PHF19, PHF2, PHF20L1, PHF21B, PHF23, PHF5A, PHGDH, PHKA1, PHKA2, PHKB, PHKG1, PHKG2, PHLPP1, PHLPP2, PHRF1, PHTF1, PHTF2, PHYH, PI4K2A, PI4K2B, PI4KA, PI4KAP2, PI4KB, PIAS1, PIAS2, PIAS3, PIAS4, PIGA, PIGB, PIGK, PIH1D1, PIK3C2A, PIK3C2B, PIK3C2G, PIK3C3, PIK3CA, PIK3CB, PIK3CD, PIK3CG, PIK3R1, PIK3R2, PIK3R3, PIK3R4, PIK3R5, PIKFYVE, PIM1, PIM2, PIM3, PIN1, PINK1, PION, PIP4K2A, PIP4K2B, PIP4K2C, PIP5K1A, PIP5K1B, PIP5K1C, PIP5KL1, PIPOX, PISD, PITPNA, PJA1, PJA2, PKD1, PKDCC, PKIA, PKIG, PKLR, PKM2, PKN1, PKN2, PKN3, PLA2G12A, PLA2G12B, PLA2G15, PLA2G2A, PLA2G2C, PLA2G4A, PLAGL1, PLAT, PLAU, PLAUR, PLCB1, PLCB3, PLCB4, PLCG1, PLCG2, PLD1, PLD2, PLD4, PLEKHJ1, PLEKHM1, PLK1, PLK2, PLK3, PLK4, PLOD1, PLOD2, PLOD3, PLP2, PLS1, PLSCR1, PLSCR3, PLXNA1, PLXNA2, PLXNA3, PLXNA4, PLXNB1, PLXNB2, PLXNB3, PLXNC1, PLXND1, PMAIP1, PMF1, PML, PMM2, PMPCB, PMS1, PMS2P3, PMVK, PNCK, PNKP, PNLDC1, PNN, PNP, PNPO, PNRC1, POFUT1, POLA1, POLA2, POLB, POLD4, POLE2, POLE3, POLG, POLG2, POLQ, POLR1A, POLR1C, POLR1E, POLR2A, POLR2C, POLR2D, POLR2E, POLR2F, POLR2H, POLR2I, POLR2K, POLR3B, POLR3C, POLR3D, POLR3E, POLR3F, POLR3K, POLRMT, PON2, PON3, POP4, POR, POT1, POU2AF1, POU2F1, POU2F2, POU5F1, POU5F2, PPAP2A, PPAP2B, PPARA, PPARD, PPARG, PPARGC1A, PPARGC1B, PPAT, PPFIBP2, PPIA, PPIB, PPIC, PPIE, PPIF, PPIG, PPIH, PPM1B, PPM1D, PPM1L, PPOX, PPP1CA, PPP1CC, PPP1R13B, PPP1R14B, PPP1R8, PPP2CA, PPP2CB, PPP2R1A, PPP2R2C, PPP2R3A, PPP2R3C, PPP2R4, PPP2R5A, PPP2R5B, PPP2R5C, PPP2R5E,

	PPP3CA, PPP3CB, PPP3CC, PPP3R1, PPP3R2, PPP4C, PPP4R1, PPT1, PPT2, PQBP1, PRAF2, PRCC, PRCP, PRDM1, PRDM4, PRDM7, PRDX1, PRDX2, PRDX5, PREB, PREP, PREX2, PRKAA1, PRKAA2, PRKAB1, PRKAB2, PRKACA, PRKACB, PRKACG, PRKAG1, PRKAG2, PRKAG3, PRKAR1A, PRKAR1B, PRKAR2A, PRKAR2B, PRKCA, PRKCB, PRKCD, PRKCE, PRKCH, PRKCI, PRKCQ, PRKCZ, PRKD2, PRKD3, PRKDC, PRKG1, PRKG2, PRKRA, PRKRIR, PRKX, PRKY, PRLHR, PRLR, PRMT1, PRMT2, PRMT3, PRNP, PROC, PROCR, PROKR1, PROKR2, PROS1, PROSC, PROX1, PRPF4, PRPF4B, PRPF6, PRPS1, PRPS1L1, PRPS2, PRPSAP2, PRR15L, PRR7, PRSS2, PRSS23, PRSS3, PRSS42, PRUNE, PSAP, PSAT1, PSEN1, PSEN2, PSENEN, PSIP1, PSKH1, PSKH2, PSMA1, PSMA3, PSMA5, PSMA7, PSMA8, PSMB1, PSMB10, PSMB2, PSMB5, PSMB7, PSMB8, PSMB9, PSMD1, PSMD10, PSMD2, PSMD3, PSMD4, PSMD5, PSMD8, PSMD9, PSME1, PSME2, PSMF1, PSMG1, PSPH, PSRC1, PTCH1, PTDSS1, PTEN, PTF1A, PTGER4, PTGFR, PTGR1, PTGS2, PTHLH, PTK2, PTK2B, PTK7, PTMS, PTP4A1, PTP4A3, PTPLA, PTPLAD1, PTPN1, PTPN11, PTPN12, PTPN2, PTPN21, PTPN22, PTPN4, PTPN5, PTPN6, PTPRF, PTPRG, PTPRK, PTRF, PTS, PTTG1, PUF60, PUM2, PURA, PUS10, PUS7L, PVALB, PVR, PVRL2, PWP1, PXK, PXMP2, PXN, PYCR1, PYCRL, PYGL, PYGO1, QARS, QDPR, QKI, QPCT, QPRT, QRFPR, QRSL1, RAB11A, RAB11FIP2, RAB1B, RAB21, RAB23, RAB27A, RAB31, RAB3GAP1, RAB4A, RAB5A, RAB5B, RAB7A, RABGGTA, RAC1, RAC3, RAD1, RAD23B, RAD50, RAD51, RAD51C, RAD54L, RAD9A, RAE1, RAF1, RAI14, RALA, RALB, RALBP1, RALGDS, RAMP1, RAN, RANBP9, RAP1A, RAP1GAP, RAP1GDS1, RARA, RARB, RARG, RARRES1, RARRES3, RASA1, RASD1, RASGRP1, RASGRP4, RASSF1, RASSF2, RASSF5, RAVER1, RB1, RBBP4, RBBP6, RBBP7, RBBP8, RBCK1, RBFOX3, RBKS, RBL1, RBL2, RBM14, RBM15, RBM15B, RBM34, RBM6, RBM8A, RBMS1, RBMX, RBP1, RBP4, RBPJ, RBX1, RCAN1, RCCD1, RCHY1, RCOR1, RDBP, RDH11, RDH14, RDX, REEP5, REL, RELA, RELB, RELN, RERG, RET, RETSAT, RFC1, RFC2, RFC5, RFK, RFNG, RFWD2, RFX2, RFX4, RFX5, RFX6, RFXANK, RG9MTD1, RGS16, RGS18, RGS19, RGS2, RGS4, RHEB, RHOA, RHOB, RHOBTB1, RHOD, RICTOR, RIMS3, RING1, RIOK1, RIOK2, RIOK3, RIPK1, RIPK2, RIPK3, RIPK4, RIT1, RNASE4, RNASEL, RNASET2, RND3, RNF11, RNF123, RNF125, RNF133, RNF138, RNF14, RNF145, RNF166, RNF167, RNF186, RNF5, RNF7, RNGTT, RNH1, RNLS, RNMT, RNPS1, ROCK1, ROCK2, ROR1, ROR2, RORC, ROS1, RP2, RPA1, RPA2, RPA3, RPF1, RPIA, RPL22, RPL39L, RPL7, RPN1, RPN2, RPP38, RPS10, RPS13, RPS14, RPS15A, RPS16, RPS19, RPS27A, RPS3, RPS3A, RPS5, RPS6, RPS6KA1, RPS6KA2, RPS6KA3, RPS6KA4, RPS6KA5, RPS6KA6, RPS6KB1, RPS6KB2, RPS6KC1, RPS6KL1, RPS7, RPS9, RPTN, RPTOR, RRAGA, RRAGB, RRAGC, RRAGD, RRM1, RRM2, RRP12, RRP1B, RRP8, RRS1, RSPH1, RSU1, RTCD1, RTEL1, RTN2, RUFY1, RUNX1, RUVBL1, RUVBL2, RXFP1, RXFP4, RXRA, RXRB, RXRG, RYBP, RYK, S100A1, S100A11, S100A13, S100A4, S100A6, S100B, S100P, S100Z, S1PR2, S1PR3, SACM1L, SAFB, SALL4, SAP18, SAR1B, SARS, SAT1, SATB1, SATB2, SBK1, SBNO1, SC5DL, SCAF8, SCAND1, SCAP, SCARB1, SCCPDH, SCMH1, SCN9A, SCNN1A, SCP2, SCRN1, SCUBE1, SCYL1, SCYL2, SCYL3, SDF2L1, SDHA, SDHB, SDHC, SDHD, SEC14L1, SEC16A, SEC24B, SEC24C, SEC24D, SEC61A2, SENP2, SENP5, SENP6, SEPHS2, SEPX1, SERINC3, SERPINA1, SERPINA5, SERPINA6, SERPINA7, SERPINB2, SERPINB5, SERPINB6, SERPINC1, SERPIND1, SERPINE1, SERPINF2, SERPINH1, SERTAD3, SESN1, SET, SETD1B, SETD7, SETD8, SETDB1, SF1, SF3B1, SFMBT1, SFN, SFPQ, SGCB, SGK1, SGK196, SGK2, SGK223, SGK3, SGK494, SGPL1, SH2B3, SH3BP4, SH3BP5, SH3BP5L, SH3GL1, SHANK2, SHANK3, SHB, SHC1, SHC4, SHH, SHMT2, SHPK, SIAH1, SIAH2, SIK1, SIK2, SIK3, SIM2, SIN3A, SIRPG, SIRT1, SIRT2, SIRT3, SIRT5, SIRT6, SIRT7, SIX2, SIX4, SKAP2, SKIV2L, SKP1, SKP2, SLAMF6, SLC11A2, SLC12A2, SLC12A7, SLC16A1, SLC16A2, SLC16A3, SLC16A4, SLC16A5, SLC16A6, SLC1A1, SLC1A4, SLC1A5, SLC1A6, SLC22A18, SLC22A23, SLC22A4, SLC22A5, SLC25A1, SLC25A10, SLC25A12, SLC25A13, SLC25A14, SLC25A15, SLC25A20, SLC25A22, SLC25A24, SLC25A28, SLC25A32, SLC25A4, SLC25A46, SLC25A5, SLC25A6, SLC27A3, SLC29A1, SLC29A2, SLC2A1, SLC2A2, SLC2A4RG, SLC2A6, SLC30A10, SLC30A2, SLC30A8, SLC35A1, SLC35A3, SLC35B1, SLC35F2, SLC36A1, SLC36A4, SLC37A4, SLC38A1, SLC38A2, SLC38A3, SLC39A6, SLC39A8, SLC3A1, SLC3A2, SLC5A6, SLC6A14, SLC6A8, SLC7A1, SLC7A11, SLC7A5, SLC7A9, SLC9A1, SLIRP, SLITRK6, SLK, SMAD1, SMAD2, SMAD3, SMAD4, SMAD5, SMAD6, SMAD7, SMARCA2, SMARCA4, SMARCA5, SMARCAD1, SMARCB1, SMARCC1, SMARCC2, SMARCD2, SMARCE1, SMC1A, SMC3, SMC4, SMG1, SMG7, SMNDC1, SMO, SMOC2, SMOX, SMS, SMU1, SMURF1, SMURF2, SMYD3, SNAI1, SNAI2, SNAI3, SNAPC1, SNAPC4, SNCA, SNF8, SNRK, SNRNP70, SNRPA, SNRPD1, SNW1, SNX11, SNX13, SNX16, SNX17, SNX2, SNX6, SNX7, SOAT1, SOAT2, SOCS1, SOCS2, SOCS3, SOCS4, SOCS5, SOD1, SOD2, SORBS3, SORD, SORT1, SOS1, SOS2, SOX10, SOX11, SOX2, SOX3, SOX4, SOX5, SP100, SP110, SP140, SP140L, SP3, SPA17, SPAG4, SPAG7, SPARC, SPDEF, SPECC1L, SPEG, SPEN, SPHK1, SPHK2, SPIB, SPIC, SPP1, SPR, SPRED2, SPRY1, SPRY2, SPRY4, SPTAN1, SPTLC1, SPTLC2, SQLE, SQRDL, SQSTM1, SRC, SRD5A1, SREBF1, SREBF2, SRF, SRGAP2, SRM, SRP14, SRPK1, SRPK2, SRPK3, SRPRB, SRPX, SRRM1, SRRT, SRSF2, SRSF3, SRSF4, SRSF8, SS18L1, SSB, SSBP2, SSBP4, SSX2, SSX3, ST13, ST14, ST3GAL5, ST6GALNAC2, ST7, STAM, STAM2, STAMBP, STAP2, STAT1, STAT2, STAT3, STAT4, STAT5A, STAT5B, STAT6, STIL, STK10, STK11, STK16, STK17A, STK17B, STK19, STK24, STK25, STK3, STK31, STK32A, STK32B, STK32C, STK33, STK35, STK36, STK38, STK38L, STK39, STK40, STMN1, STRADA, STRADB, STRN4, STUB1, STX16, STX1A, STX4, STXBP1, STXBP2, STYK1, SUB1, SUCLA2, SUCLG1, SUCLG2, SUCNR1, SUFU, SULT1A1, SULT1A2, SULT1A3, SULT2A1, SULT2B1, SUOX, SUPT16H, SUPT3H, SUPT4H1, SUPT5H, SUPV3L1, SUV39H1, SUZ12, SV2A, SWAP70, SYF2, SYK, SYN2, SYNE1, SYNE2, SYNGR3, SYPL1, SYT1, TAAR1, TAAR6, TAAR8, TAAR9, TAB2, TAB3, TACC3, TADA2A, TADA3, TAF1, TAF12, TAF13, TAF15, TAF1B, TAF1L, TAF5L, TAF8, TAGAP, TAL1, TAL2, TANK, TAOK1, TAOK2, TAOK3, TAP1, TARBP1, TARBP2, TARDBP, TARS, TASP1, TATDN2, TBC1D15, TBC1D2B, TBC1D9B, TBCB, TBCK, TBK1, TBL3, TBP, TBPL1, TBX2, TBX20, TBX3, TBX5, TBXA2R, TCEA1, TCEA2, TCEAL4, TCEB3, TCEB3C, TCERG1, TCF12, TCF3, TCF4, TCF7, TCF7L1, TCF7L2, TCFL5, TCIRG1, TCTA, TCTN1, TDRD3, TDRD9, TEAD1, TEAD2, TEAD4, TEC, TEK, TERF1, TERF2IP, TES, TESK1, TESK2, TEX10, TEX14, TF, TFAP2A, TFAP4, TFCP2, TFDP1, TFDP2, TFE3, TFF1, TFF2, TFG, TFPI, TFRC, TGDS, TGFA, TGFB1, TGFBR1, TGFBR2, TGFBR3, TGIF2LY, TGM2, TH, THADA, THAP11, THBD, THBS1, THRAP3, TIAM1, TIE1, TIMELESS, TIMM17B, TIMM22, TIMM50, TIMM9, TIMP1, TIMP2, TIMP3, TIMP4, TINF2, TIPARP, TIRAP, TJP1, TJP2, TK1, TK2, TKT, TLE1, TLE2, TLE3, TLK1, TLK2, TLR2, TLR4, TLR5, TLR7, TLR8, TLR9, TM7SF2, TM9SF2, TM9SF3, TMCO1, TMED10, TMED7, TMED9, TMEM109, TMEM11, TMEM110, TMEM127, TMEM154, TMEM174, TMEM2, TMEM5, TMEM50A, TMEM97, TMSB4XP8, TNF, TNFAIP1, TNFAIP3, TNFRSF10A, TNFRSF10B, TNFRSF11A, TNFRSF12A, TNFRSF13B, TNFRSF13C, TNFRSF14, TNFRSF17, TNFRSF18, TNFRSF19, TNFRSF1A, TNFRSF21, TNFRSF6B, TNFSF10, TNFSF13, TNFSF13B, TNFSF15, TNFSF4, TNFSF8, TNIK, TNIP1, TNK2, TNKS2, TNNI3K, TNPO3, TOLLIP, TOMM22, TOMM34, TOMM40, TOMM70A, TOP1, TOP2A, TOP2B, TOPBP1, TOR1A, TOX2, TP53, TP53BP1, TP53BP2, TP53RK, TP53TG1, TPD52L2, TPD52L3, TPI1, TPK1, TPM1, TPMT, TPR, TPRKB, TRA2B, TRADD, TRAF1, TRAF2, TRAF3, TRAF4, TRAF5, TRAF6, TRAF7, TRAFD1, TRAK2, TRAM2, TRAP1, TRAPPC3, TRAPPC6A, TRERF1, TRIB1, TRIB2, TRIB3, TRIM13, TRIM16, TRIM2, TRIM24, TRIM27, TRIM29, TRIM32, TRIM38, TRIO, TRIP10, TRIP13, TRPM2, TRPM5, TRPM6, TRPM7, TRPS1, TRRAP, TSC1, TSC2, TSC22D1, TSC22D3, TSEN2, TSG101, TSHZ3, TSKU, TSLP, TSN, TSPAN3, TSPAN4, TSPAN6, TSPAN8, TSSC4, TSSK1B, TSSK2, TSSK3, TSSK4, TSSK6, TST, TSTA3, TTBK1, TTBK2, TTC3, TTF1, TTK, TTN, TTR, TUBA1A, TUBB2A, TUBB2C, TUBB3, TUBB6, TUBD1, TWF2, TWIST1, TWIST2, TXK, TXLNA, TXN, TXNDC3, TXNDC9, TXNIP, TXNL1, TXNL4B, TXNRD1,

	TXNRD2, TXNRD3, TYK2, TYMS, TYRO3, UAP1, UBA52, UBAC2, UBAP1, UBAP2L, UBASH3A, UBB, UBC, UBE2A, UBE2C, UBE2D1, UBE2D3, UBE2E3, UBE2J1, UBE2K, UBE2L3, UBE2L6, UBE2N, UBE2V1, UBE2Z, UBE3A, UBE3B, UBE3C, UBE4A, UBL5, UBP1, UBQLN2, UBR7, UBTF, UCHL1, UCK1, UCK2, UCKL1, UFD1L, UFM1, UGCG, UGDH, UGP2, UGT1A1, UGT1A3, UGT1A6, UGT1A9, UGT2B28, UHMK1, UIMC1, ULK1, ULK2, ULK3, ULK4, UMODL1, UNC13B, UNC5C, UNC5D, UNG, UQCR11, UQCRB, UQCRC1, UQCRFS1, UROD, USF1, USP1, USP12, USP14, USP15, USP16, USP20, USP22, USP32, USP47, USP6NL, USP7, USP9X, UTP14A, UTP18, UTS2R, UVRAG, UXT, VAMP3, VAMP7, VAPA, VAPB, VARS, VAT1, VAV3, VCAN, VCP, VDAC1, VDR, VEGFA, VEGFB, VEGFC, VGLL4, VHL, VIM, VIPR1, VKORC1, VN1R2, VN1R4, VN1R5, VPS26A, VPS28, VPS72, VPS8, VRK1, VRK2, VRK3, VTI1A, WARS, WARS2, WASF3, WDR6, WDR61, WDR67, WDR7, WDTC1, WEE1, WFDC2, WFS1, WHSC1, WIF1, WIPF2, WNK1, WNK2, WNK3, WNK4, WNT1, WNT10B, WNT11, WNT5A, WNT7B, WNT9A, WNT9B, WRB, WRN, WT1, WTAP, WWOX, WWTR1, XBP1, XIAP, XK, XPC, XPNPEP1, XPNPEP3, XPO7, XPR1, XRCC4, XRCC5, XRCC6, XRCC6BP1, XYLB, YAF2, YAP1, YARS, YBX1, YEATS4, YES1, YKT6, YME1L1, YSK4, YTHDF1, YTHDF2, YWHAH, YWHAQ, YWHAZ, YY1, ZAK, ZAP70, ZBED1, ZBTB20, ZBTB24, ZBTB25, ZBTB26, ZBTB41, ZBTB45, ZBTB46, ZBTB48, ZBTB49, ZBTB5, ZC3H4, ZC3HC1, ZDHHC6, ZEB1, ZER1, ZFAND6, ZFAT, ZFP1, ZFP112, ZFP161, ZFP28, ZFP3, ZFP36, ZFP36L1, ZFP36L2, ZFX, ZFYVE19, ZGPAT, ZIC2, ZIM3, ZKSCAN2, ZMIZ1, ZMIZ2, ZMPSTE24, ZMYM2, ZMYND11, ZNF114, ZNF131, ZNF133, ZNF134, ZNF136, ZNF138, ZNF140, ZNF143, ZNF169, ZNF174, ZNF175, ZNF180, ZNF182, ZNF195, ZNF200, ZNF202, ZNF207, ZNF212, ZNF213, ZNF217, ZNF219, ZNF22, ZNF232, ZNF238, ZNF24, ZNF263, ZNF266, ZNF267, ZNF268, ZNF273, ZNF274, ZNF277, ZNF281, ZNF296, ZNF300, ZNF317, ZNF318, ZNF32, ZNF324, ZNF331, ZNF333, ZNF350, ZNF354B, ZNF365, ZNF366, ZNF384, ZNF385B, ZNF395, ZNF398, ZNF418, ZNF426, ZNF432, ZNF433, ZNF434, ZNF436, ZNF444, ZNF449, ZNF451, ZNF454, ZNF462, ZNF473, ZNF486, ZNF488, ZNF490, ZNF496, ZNF501, ZNF502, ZNF517, ZNF519, ZNF521, ZNF524, ZNF543, ZNF546, ZNF548, ZNF551, ZNF554, ZNF558, ZNF559, ZNF561, ZNF562, ZNF563, ZNF567, ZNF57, ZNF572, ZNF576, ZNF577, ZNF581, ZNF582, ZNF583, ZNF585A, ZNF586, ZNF589, ZNF595, ZNF596, ZNF597, ZNF599, ZNF607, ZNF608, ZNF610, ZNF611, ZNF619, ZNF621, ZNF623, ZNF624, ZNF625, ZNF626, ZNF629, ZNF643, ZNF653, ZNF658, ZNF662, ZNF668, ZNF669, ZNF671, ZNF672, ZNF673, ZNF677, ZNF678, ZNF684, ZNF689, ZNF692, ZNF707, ZNF717, ZNF74, ZNF764, ZNF768, ZNF77, ZNF785, ZNF786, ZNF787, ZNF791, ZNF792, ZNF8, ZNF805, ZNF830, ZNF85, ZNF92, ZNHIT3, ZPBP2, ZRANB2, ZRSR2, ZSCAN1, ZSCAN29, ZSCAN4, ZSWIM2, ZW10
Over expression	(AC074117.10), (AC074117.10_MUT), (AC084809.2), (AC098617.1), (AC115618.1), (AK097618), (AL110181), (AL110181_MUT), (ASNSD1), (AX746851), (AX747325), (B3GNT5), (CBR3-AS1), (CD27-AS1), (CHST2), (CITF22-92A6.1), (CTA-292E10.6), (CTC-480C2.1), (CTD-2270L9.4), (CTD-3131K8.2), (DDIT3), (DLG5-AS1), (EIF1B), (EMC3-AS1), (EMX2OS), (ENTPD1-AS1), (ERVK3-1), (FAM220A), (FAM83H-AS1), (FAM83H-AS1_MUT), (FOXD3-AS1), (FOXD3-AS1_MUT), (G008259), (G032161), (G036240), (G047227), (G048207), (G054633), (G063514), (G070104), (G083755), (G085301), (HLA-F-AS1), (LINC00240), (LINC00667), (LINC00842), (LINC01004), (LINC01184), (LINC01399), (LINC01589), (LINC01589_MUT), (LINC02081.2), (LIPE-AS1), (LOC100130987), (LOC100505549), (LOC100506302), (LOC101927497), (LOC101927989), (LOC102724434), (LOC105372440), (LOC284241), (LOC400553), (LOC401320.2), (LOC728730), (LOXL1-AS1), (MAF1), (MID1), (MIF-AS1), (MIR924HG), (MIRLET7BHG), (PCBP1-AS1), (PCBP1-AS1_MUT), (PIK3R1), (PNRC2), (PTP4A1), (RGMB-AS1), (RP11-138J23.1), (RP11-195F19.9), (RP11-212P7.2), (RP11-212P7.2_MUT), (RP11-234O6.2), (RP11-327P2.5), (RP11-428J1.4), (RP11-464F9.20), (RP11-467L20.10), (RP11-505K9.1), (RP11-505K9.1_MUT), (RP11-539I5.1), (RP11-574K11.24), (RP11-59D5_B.2), (RP11-680A11.5), (RP11-712L6.5), (RP13-1032I1.7), (RP3-508I15.9), (RP6-99M1.2), (SLC35A4), (SLC35A4_MUT), (SNHG1_MUT), (SOCS2), (TCONS_00019670), (TMCC1-AS1_MUT), (UBA6-AS1), (UBA6-AS1_MUT), (USP2-AS1), (ZNF788.1), A1BG-AS1, A2M, A4GALT, AATK-AS1, ABAT, ABCB5, ABCB9, ABCC3, ABCF2, ABCF3, ABCG8, ABHD2, ABHD4, ABL1, ABL1_G2A, ABL1_T315I, ABTB1, ACAA1, ACADM, ACADS, ACADSB, ACAT2, ACD, ACLY, ACTB, ACTG1, ACTL6A, ACTN2, ACTN4, ACTR3, ACVR1, ACVR1B, ACVR1C, ACVR2A, ACVR2B, ACVR2B-AS1, ACY1, ADA, ADAM17, ADAP2, ADAT1, ADC, ADCK3, ADCK4, ADCY3, ADCY6, ADCY9, ADH5, ADI1, ADK, ADORA2A, ADORA2B, ADRB2, ADRBK1, ADSL, ADSS, AES, AFF1, AFF4, AFG3L2, AFP, AGL, AGPAT1, AGPAT2, AGT, AGTR1, AHCY, AHDC1, AHR, AIFM1, AIRE, AK1, AK2, AKAP8, AKR1A1, AKR1B1, AKR1C1, AKR1C2, AKR1C3, AKT1, AKT1S1, AKT2, AKT3, AKTIP, ALDH1A1, ALDH1B1, ALDH2, ALDH3A1, ALDH3A2, ALDH3B1, ALDH3B2, ALDH6A1, ALDOA, ALDOC, ALG2, ALG5, ALG9, ALK, ALK_F1174L, ALK_R1275Q, ALK_T1151M, ALPK1, ALPK2, ALS2CR7, AMBP, AMHR2, AMIGO3, AMPD2, ANAPC2, ANAPC5, ANG, ANKRD49, ANKZF1, ANPEP, ANXA1, ANXA2, ANXA5, ANXA7, AOC3, AOF2, AP1S2, AP2M1, APAF1, APC, APEH, APEX1, APOA1, APOA2, APOBEC3H, APOC2, APOC3, APOH, APOM, APRT, APTR, AR, ARAF, ARF1, ARF6, ARFIP2, ARG1, ARG2, ARHGAP26, ARHGEF19, ARHGEF2, ARHGEF3, ARHGEF7, ARHGEF9, ARID3A, ARIH1, ARIH2, ARL4C, ARMC5, ARNT, ARNTL, ARNTL2, ARPC1A, ARPC1B, ARPC3, ARPC4, ARRB1, ARSI, ASAH1, ASAP2, ASB16-AS1, ASB3, ASCC3, ASCL2, ASCL4, ASF1A, ASF1B, ASGR2, ASH2L, ASMTL, ASNA1, ASNS, ASPH, ASPHD1, ASS1, ATAD1, ATF1, ATF2, ATF3, ATF4, ATF6, ATF6B, ATF7, ATG10, ATG16L1, ATG16L2, ATG3, ATG4A, ATG5, ATG7, ATIC, ATM, ATMIN, ATOH1, ATOX1, ATP1A1-AS1, ATP1A3, ATP1B1, ATP2A1-AS1, ATP5A1, ATP5B, ATP5C1, ATP5D, ATP5F1, ATP5H, ATP5L, ATP5S, ATP6V0A1, ATP6V0B, ATP6V0C, ATP6V1D, ATP6V1F, ATPIF1, ATXN3, AURKA, AURKAIP1, AURKB, AXIN2, AXL, AZGP1, B2M, B3GAT3, B3GNT1, B4GALT3, B4GALT4, BACH1, BAD, BAG1, BAG6, BAIAP2-AS1, BAMBI, BAP1, BARD1, BATF3, BAX, BAZ2B, BCAT2, BCCIP, BCKDK, BCL10, BCL2L1, BCL2L11, BCL2L2, BCL6, BCL6B, BCR, BCR-ABL, BCR-ABL_A269V, BCR-ABL_D276V, BCR-ABL_E236K, BCR-ABL_E279K, BCR-ABL_E281K, BCR-ABL_E282D, BCR-ABL_E285K, BCR-ABL_E286K, BCR-ABL_E292Q, BCR-ABL_E316D, BCR-ABL_E450K, BCR-ABL_E494A, BCR-ABL_E499K, BCR-ABL_F311L, BCR-ABL_F317L, BCR-ABL_F382A, BCR-ABL_F486S, BCR-ABL_G250E, BCR-ABL_G321D, BCR-ABL_G372R, BCR-ABL_H396P, BCR-ABL_H396R, BCR-ABL_I352K, BCR-ABL_I502M, BCR-ABL_K271N, BCR-ABL_L248R, BCR-ABL_L451M, BCR-ABL_M244I, BCR-ABL_M278K, BCR-ABL_M472I, BCR-ABL_N374S, BCR-ABL_Q252H, BCR-ABL_Q300H, BCR-ABL_Q346H, BCR-ABL_T224A, BCR-ABL_T315I, BCR-ABL_V289F, BCR-ABL_V339A, BCR-ABL_Y253H, BCR-ABL_Y440C, BECN1, BEND5, BHLHE40, BHMT, BHMT2, BID, BIK, BIRC2, BLCAP, BLK, BLMH, BLNK, BLVRA, BLZF1, BMI1, BMP2, BMP4, BMPR1A, BMPR1B, BMPR2, BMX, BNIP1, BNIP3, BNIP3L, BOLA1, BPHL, BRAF, BRAF_A727V, BRAF_D594V, BRAF_E586K, BRAF_G464V, BRAF_G466A, BRAF_G466E, BRAF_G466V, BRAF_G469A, BRAF_G469E, BRAF_G596R, BRAF_K601E, BRAF_V600E, BRCA1, BRD3, BRD4, BRD9, BRF2, BRMS1L, BRP44, BRPF1, BTBD1, BTBD12, BTC, BTG1, BTG3, BTK, BTRC, BUB1B, BUB3, BUD13, BUD31, BZW2, C13ORF42, C14ORF101, C15ORF23, C15ORF53, C16orf5, C16orf80, C17ORF49, C19ORF48, C19orf10, C1ORF106, C1ORF150, C1ORF25, C1QA, C1RL-AS1, C1S, C2, C20ORF194, C20ORF204, C20ORF72, C5ORF56, C5orf62, C9ORF11, CAB39, CALCOCO2, CALM1, CALM2, CALM3, CALR, CALU, CAMK1, CAMK2A, CAMK2D, CAMK2G, CAMK4, CAMKK1, CAMKK2, CAMKV, CAMLG, CANT1, CAPG, CAPN1, CAPNS2, CARD11, CARD8-AS1, CARD9, CARS, CASC15, CASC2, CASD1, CASK, CASP14, CASP2, CASP3, CASP6, CASP7, CASP8, CASP9, CAT, CAV1, CBFB, CBLB, CBLC, CBLL1, CBR1, CBR3, CBS, CBX1, CBX3,

	CBX6, CBX7, CBX8, CCBL1, CCDC144NL-AS1, CCDC150, CCDC6-PDGFRB, CCDC71, CCDC79, CCDC90A, CCDC92, CCL2, CCNC, CCND1, CCND2, CCND3, CCNE1, CCNF, CCNG1, CCNH, CCNL1, CCP110, CCRL2, CCRN4L, CCT7, CD14, CD19, CD24, CD2BP2, CD320, CD36, CD3E, CD40, CD44, CD55, CD58, CD59, CD70, CD79B, CD81, CD83, CD97, CDC20, CDC25A, CDC25B, CDC25C, CDC2L1, CDC2L5, CDC2L6, CDC42, CDC42SE1, CDC45, CDC7, CDCA4, CDCA8, CDH9, CDIPT, CDK1, CDK10, CDK16, CDK17, CDK2, CDK3, CDK4, CDK5, CDK6, CDK7, CDK8, CDK9, CDKAL1, CDKL4, CDKN1A, CDKN1B, CDKN2C, CDO1, CDX1, CDX2, CEBPA, CEBPE, CEBPG, CEP55, CEP72, CEPT1, CERS2, CERS3, CERS4, CETN3, CFB, CFL1, CFLAR, CGREF1, CGRRF1, CHAF1B, CHCHD7, CHD1, CHD2, CHEK1, CHEK2, CHGA, CHMP4A, CHMP6, CHN1, CHRAC1, CHRM3, CHST12, CHSY3, CHTF8, CHUK, CIAPIN1, CIITA, CIQTNF6, CIRBP, CISD1, CISH, CKB, CKMT2-AS1, CKS1B, CLCN5, CLDN18, CLEC2D-V1, CLEC2D-V2, CLECL1, CLIC4, CLK1, CLK2, CLK3, CLOCK, CLPB, CLPTM1, CLTA, CMAP-ERGK004, CMAP-ERGK004.DOX, CMAP-ERGK005, CMAP-ERGK005.DOX, CMPK1, CNBD1, CNDP2, CNOT3, CNOT4, CNOT7, CNOT8, CNPY3, COASY, COBL, COG2, COG7, COMT, COPS2, COPS4, COPS5, COPZ1, COQ3, CORO1A, COX4I1, COX5A, COX5B, COX6B2, COX7B, CPD, CPE, CPLX2, CPS1, CPSF1, CPSF4, CPT1A, CPXCR1, CPZ, CRABP1, CRCP, CREB1, CREB3, CREB3L1, CREB3L2, CREB3L4, CREB5, CREBBP, CREM, CRK, CRKL, CRNDE, CROT, CRX, CRY1, CRYAA, CRYBB1, CRYGC, CRYZ, CS, CSDA, CSDC2, CSF1R, CSF3, CSK, CSNK1A1, CSNK1A1L, CSNK1D, CSNK1E, CSNK1G2, CSNK1G3, CSNK2A1, CSNK2A2, CSNK2B, CTB-41I6.1, CTBP1, CTBP1-AS2, CTBP2, CTC-338M12.4, CTCF, CTCFL, CTGF, CTH, CTLA4, CTNNB1, CTNNBIP1, CTSD, CTSK, CTSL1, CTSL2, CTTN, CTXND1, CUL1, CUL2, CUL3, CUL4B, CUL5, CXADR, CXCL1, CXCL2, CXCR2, CXCR4, CXCR7, CXXC4, CYB561, CYB5A, CYC1, CYCS, CYLC2, CYLD, CYP1A1, CYP20A1, CYP24A1, CYP2E1, CYP3A5, CYP51A1, DACH1, DACH2, DANCR, DAP, DAPK1, DAPK2, DARC, DARS, DAXX, DBI, DCAF4L2, DCAF8, DCAMKL2, DCK, DCLK2, DCLRE1B, DCP1A, DCPS, DCST1-AS1, DCTN6, DCXR, DDAH2, DDB2, DDIT3, DDIT4, DDOST, DDR1, DDR2, DDX49, DECR1, DEK, DENND2D, DEPDC1, DEPDC5, DEPDC6, DEPDC7, DEPTOR, DERL1, DET1, DFFA, DFFB, DGCR14, DGKA, DGKZ, DHCR24, DHDDS, DHFR, DHH, DHRS2, DHRS3, DHRS7, DHX29, DHX57, DHX8, DIABLO, DICER1, DICER1-AS1, DIDO1, DKC1, DKFZP761P0423, DKK1, DLC1, DLD, DLEU1, DLGAP5, DLL1, DLX1, DLX2, DLX3, DLX4, DLX6, DMD, DMPK, DMRT1, DMRT3, DMRTA1, DMRTC2, DMTF1, DNAJA3, DNAJB1, DNAJB1-PRKACA, DNAJB2, DNAJB5, DNAJB6, DNAJB9, DNAJC1, DNAJC12, DNAJC15, DNAJC2, DNAJC5B, DNASE2, DNM1L, DNMT3A, DNTTIP2, DOK1, DOK4, DOT1L, DPF2, DPF3, DPY30, DPYD, DR1, DRD1, DSE, DSTYK, DTNA, DTX2, DTX3L, DTX4, DUS3L, DUSP1, DUSP14, DUSP18, DUSP2, DUSP22, DUSP28, DUSP3, DUSP4, DUSP6, DUT, DVL1, DVL2, DVL3, DYNLL1, DYNLL2, DYNLT3, DYRK1B, DYRK2, DYRK3, DYRK4, DZIP3, E2F1, E2F3, E2F6, E2F8, E4F1, EAPP, EBF1, EBF3, EBF4, EBNA1BP2, EBP, ECD, ECH1, ECHS1, ECSIT, EDNRB, EED, EEF1D, EFCAB10, EFCAB2, EFHC1, EFNB2, EFNB3, EGFL8, EGFR, EGFR_DEL3, EGFR_G719S, EGFR_INSNPG, EGFR_L858R, EGFR_L858R_T790M, EGFR_T790M, EGFR_VIII, EGLN1, EGLN3, EGR1, EGR2, EGR3, EHF, EHHADH, EHMT2, EIF1AX, EIF1B, EIF2A, EIF2AK1, EIF2AK2, EIF2AK4, EIF2B2, EIF2C1, EIF2S2, EIF3G, EIF3H, EIF3J-AS1, EIF4A2, EIF4B, EIF4E, EIF4E3, EIF4EBP1, EIF4EBP2, EIF4EBP3, EIF4H, EIF5A, ELAC2, ELANE, ELF1, ELF2, ELF3, ELF4, ELFN1-AS1, ELK1, ELK3, ELK4, ELL3, ELMOD3, ELOVL6, EMC3-AS1, EMD, ENOPH1, ENOSF1, ENST00000265296, ENST00000356364, ENTPD3-AS1, EP400, EPHA2, EPHA3, EPHB1, EPHB6, EPHX1, EPHX2, EPO, EPS8, ERAP1, ERBB2, ERBB2IP, ERBB3, ERBB4, ERBB4_E836K, ERBB4_E872K, ERCC1, ERCC3, ERCC5, ERCC6, ERF, ERG, ERGIC1, ERGIC2, ERH, ERLIN1, ERN1, ERO1L, ERRFI1, ESD, ESR1, ESR2, ESRG, ESRRA, ESRRG, ESYT1, ETFA, ETFB, ETNK1, ETS1, ETS2, ETV1, ETV5, ETV6, ETV6-PDGFRB, ETV7, EVL, EWSR1, EXOC1L, EXOC2, EXOC3, EXOC6, EXOSC4, EXOSC8, EXT1, EXT2, EYA1, EYS, EZH1, EZH2, F10, F11, F2R, F2RL1, F3, FABP1, FABP4, FABP6, FADD, FADS3, FAIM, FAM102A, FAM114A2, FAM116A, FAM120A, FAM127A, FAM134C, FAM171B, FAM173B, FAM196A, FAM200A, FAM20B, FAM3C, FAM5C, FANCG, FARSA, FARSB, FAS, FASLG, FASTK, FASTKD1, FASTKD5, FBP1, FBXL10, FBXL12, FBXL13, FBXL14, FBXL19-AS1, FBXL20, FBXO10, FBXO11, FBXO15, FBXO16, FBXO18, FBXO28, FBXO3, FBXO34, FBXO36, FBXO38, FBXO40, FBXO42, FBXO46, FBXO5, FBXO7, FBXW11, FBXW7, FCGR2A, FCGR3B, FCHO1, FDFT1, FDPS, FDX1, FDXR, FECH, FEN1, FER, FERD3L, FEZ1, FEZF1-AS1, FEZF2, FFAR1, FGA, FGB, FGD5-AS1, FGF1, FGF10, FGF9, FGFR1, FGFR1OP, FGFR2, FGFR3, FGFRL1, FGG, FGR, FH, FHL2, FIBP, FIGF, FIS1, FKBP14, FKBP3, FKBP4, FLI1, FLJ23356, FLJ25006, FLJ37453, FLJ46906, FLT1, FLT3, FLT3_D835Y, FLT3_ITD, FLT4, FOS, FOSL1, FOSL2, FOXA1, FOXA3, FOXC2, FOXD4L6, FOXJ1, FOXJ2, FOXM1, FOXN2, FOXN3, FOXO1, FOXO3, FOXO4, FOXP2, FOXP3, FOXP4, FOXP4-AS1, FOXR1, FOXR2, FRK, FRS2, FRS3, FTCDNL1, FTH1, FTL, FTSJ1, FURIN, FUS, FUT1, FUT2, FXR2, FXYD2, FYN, FZD4, FZD7, G2E3, G3BP1, G3BP2, G6PC, G6PD, GAA, GAB1, GAB2, GABARAPL1, GABBR1, GABPB1, GABPB2, GABRA1, GABRA6, GADD45A, GADD45B, GALE, GALNS, GALNT14, GALNTL5, GALR1, GALR2, GALR3, GALT, GAMT, GAPDH, GAS2L1, GAS5, GAS7, GATA2, GATA2-AS1, GATA3, GATA3-AS1, GATAD2A, GATAD2B, GBA, GBGT1, GBP2, GCA, GCAT, GCDH, GCET2, GCK, GCLC, GCLM, GCM1, GCM2, GDI1, GDPD5, GEN1, GFOD1, GFPT2, GGA2, GGCX, GGH, GGPS1, GGTLC1, GJA1, GK, GLDC, GLI1, GLI3, GLIPR1, GLOD4, GLRX, GLUL, GM2A, GMCL1, GMDS, GMEB1, GMNN, GMPR2, GNA11, GNA13, GNA15, GNAI1, GNAI2, GNAI3, GNAS, GNAZ, GNB1, GNB1L, GNB2, GNB5, GNG4, GNG5, GNG8, GNGT2, GNPDA1, GOLGA2, GON4L, GOPC, GOT1, GOT2, GPATCH8, GPBAR1, GPC1, GPER, GPR101, GPR107, GPR114, GPR119, GPR128, GPR132, GPR137B, GPR139, GPR141, GPR146, GPR148, GPR151, GPR152, GPR156, GPR160, GPR161, GPR172A, GPR183, GPR26, GPR3, GPR34, GPR35, GPR37, GPR39, GPR4, GPR52, GPR55, GPR65, GPR83, GPR84, GPR87, GPRC5B, GPS2, GPX7, GRAMD1B, GRAP, GRB10, GRB2, GRB7, GRHL3, GRK4, GRK6, GRK7, GRN, GRPR, GS1-115G20.2, GS1-124K5.4, GSC, GSDMB, GSG2, GSK3A, GSK3B, GSTM1, GSTM2, GSTP1, GSTT1, GSTZ1, GTF2A1, GTF2A2, GTF2B, GTF2E2, GTF2F1, GTF2F2, GTF2H2, GTF2H3, GTF2IRD2, GTPBP8, GUK1, GUSB, GZMA, H19, H1FOO, H2AFX, H2AFY, H2AFY2, HADHA, HADHB, HAGH, HAGLR, HAL, HAND1, HAND2, HAPLN1, HAT1, HAUS1, HAVCR1, HAVCR2, HAX1, HBE1, HBP1, HCAR1, HCK, HDAC1, HDAC10, HDAC11, HDAC2, HDAC3, HDAC4, HDAC6, HDAC8, HECW2, HERC4, HERPUD1, HERPUD2, HES2, HESX1, HEXIM1, HEY1, HEY2, HIF1A, HIF1AN, HINFP, HINT1, HIPK2, HIPK4, HIST1H1A, HIST1H1B, HIST1H1C, HIST1H1E, HIST1H1T, HIST1H2AC, HIST1H2AL, HIST1H2BD, HIST1H2BK, HIST1H3A, HIST1H3B, HIST2H2BE, HK1, HLA-A, HLA-B, HLA-DMB, HLA-DPB1, HLA-DRA, HLA-DRB1, HLA-DRB3, HLA-DRB4, HLA-DRB5, HLF, HLX, HMBOX1, HMG20B, HMGA1, HMGB1, HMGB2, HMGB3, HMGB4, HMGCL, HMGCR, HMGCS2, HMGN3, HMGN4, HMGN5, HMMR, HMOX1, HMOX2, HN1L, HNF4A, HNMT, HOGA1, HOMEZ, HOOK2, HOPX, HORMAD2, HOTAIRM1, HOXA-AS2, HOXA-AS3, HOXA1, HOXA10, HOXA10-AS, HOXA2, HOXA3, HOXA5, HOXA6, HOXA9, HOXB-AS3, HOXB13, HOXB5, HOXB7, HOXC10, HOXC11, HOXC13-AS, HOXC4, HOXC9, HOXD10, HOXD9, HP1BP3, HPD, HPGD, HPN, HPRT1, HRAS, HRH1, HRH4, HRSP12, HS2ST1, HSBP1, HSD17B10, HSD17B11, HSD17B2, HSD17B4, HSD17B7, HSD17B8, HSF1, HSF1_H2F2, HSF2, HSF5, HSP90AA1, HSP90AB1, HSP90B1, HSPA14, HSPA2, HSPA5, HSPA8, HSPA9, HSPB8, HSPB9, HSPBAP1, HSPD1, HTATIP2, HTATSF1, HTD2, HTR2C, HTR3B, HTR4, HUS1, HYAL1, HYAL2, IBTK, ICAM1, ICAM3, ICK, ICMT, ID1, ID2, ID3, IDE, IDH1, IDH2, IDH3A, IDH3B, IDH3G, IER3, IFI16, IFI30, IFIH1, IFIH1-V2, IFIT5, IFNA10, IFNAR2, IFNB1, IFNG, IFNGR1, IGF1R, IGF2, IGF2BP3, IGFBP4, IGFBP5, IGFBP6, IGFBP7, IGHA2, IGHM, IGHMBP2, IGSF8, IKBIP, IKBKB, IKBKE, IKBKG, IKZF1, IKZF2, IKZF5, IL10, IL12A, IL13, IL13RA1, IL13RA2, IL15, IL18, IL18RAP, IL1B, IL1R1, IL1R2,

	IL1RAP, IL2, IL20, IL21R, IL2RB, IL4, IL6R, IL7R, IL8, ILF2, ILF3, ILK, ILKAP, IMPA1, IMPDH1, IMPDH2, ING1, ING2, ING3, INHBE, INPP1, INS, INSIG1, INSRR, INTS12, IP6K1, IP6K2, IQANK1, IQSEC1, IRAK1, IRAK2, IRAK4, IREB2, IRF2, IRF3, IRF4, IRF5, IRF6, IRF8, IRF9, IRGM, IRS1, ISG20, ISX, ITGAE, ITGB2, ITK, ITLN1, ITPK1, ITPKB, JAG1, JAK1, JAK2, JAK3, JAKMIP2-AS1, JAZF1, JMJD1A, JMJD1B, JMJD2B, JMJD2D, JMJD4, JMJD5, JMJD6, JUN, JUNB, KARS, KAT5, KAT7, KBTBD4, KBTBD8, KCMF1, KCNA1, KCNA3, KCNA6, KCNC1, KCNJ11, KCNK1, KCNN4, KCNQ1, KCNS2, KCNS3, KCTD1, KCTD17, KCTD3, KCTD8, KDELR2, KDELR3, KEAP1, KIAA0317, KIAA0415, KIAA0494, KIAA1279, KIAA1683, KIF14, KIF20A, KIF21B, KIF2B, KIF2C, KIF9, KIR3DL2, KISS1R, KITLG, KLF10, KLF11, KLF12, KLF17, KLF3, KLF4, KLF6, KLF9, KLHDC1, KLHDC2, KLHDC3, KLHDC9, KLHL1, KLHL10, KLHL12, KLHL18, KLHL2, KLHL28, KLHL29, KLHL3, KLHL32, KLHL34, KLHL36, KLHL6, KLHL9, KLK6, KLRB1, KLRB1-I168T, KLRC1, KLRC2, KNG1, KPNA3, KRAS, KRT26, KRTAP5-9, KSR, KSR2, KYNU, LACC1, LAGE3, LAMTOR3, LANCL1, LAP3, LARP1, LARP2, LARP4, LARP6, LARS, LARS2, LASS2, LASS3, LASS4, LBR, LBX1-AS1, LBX2-AS1, LCE1B, LCK, LCMT1, LCN2, LCOR, LCORL, LDHA, LDHB, LEF1, LEPRE1, LGALS1, LGALS2, LGALS3, LGALS8, LGALS8-AS1, LGMN, LGR5, LHPP, LHX4, LHX8, LHX9, LIAS, LIFR-AS1, LIG1, LIG3, LIM2, LIMK2, LIN28, LINC-C7ORF23, LINC-CTDSP2-1, LINC-LAMA1-5, LINC-LRFN5-1, LINC-MAP3K9-1, LINC-SPANXB1-2, LINC-WNT8B, LINC-ZNF681-4, LINC-ZNF726-1, LINC00094, LINC00115, LINC00265, LINC00471, LINC00479, LINC00493, LINC00526, LINC00634, LINC00668, LINC00886, LINC00888, LINC00909, LINC00938, LINC01003, LINC01011, LINC01089, LINC01311, LINC01426, LINC01572, LINC01578, LINC01719, LINC01873, LINC02001, LINC02081, LINC02228, LINC02323, LINC02381, LINC02486, LINP1, LIPA, LIPH, LIPT1, LL21NC02-21A1.1, LMNA, LMTK2, LMX1A, LMX1B, LNX1, LOC100128386, LOC100128885, LOC100129434, LOC100132215, LOC100133315, LOC100287387, LOC100499489, LOC100506100, LOC100506411, LOC100506797, LOC100507373, LOC100507437, LOC100507507, LOC100507599, LOC100996720, LOC100996842, LOC101927021, LOC101927027, LOC101927151, LOC101927746, LOC101927752, LOC101927809, LOC101927855, LOC101928076, LOC101928433, LOC101929021, LOC101929056, LOC101929099, LOC101929147, LOC101929243, LOC101929613, LOC101929748, LOC101929897, LOC101929977, LOC101930370, LOC102606465, LOC102723335, LOC102723373, LOC102724330, LOC102724532, LOC102725044, LOC105274304, LOC105369147, LOC105369340, LOC105370333, LOC105371361, LOC105371453, LOC105372233, LOC105372480, LOC105372482, LOC105373159, LOC105373748, LOC105375014, LOC105375304, LOC105375334, LOC105376114, LOC105376736, LOC105376844, LOC105377283, LOC105377567, LOC105379049, LOC105379476, LOC107983971, LOC107984396, LOC107984814, LOC107984853, LOC107984895, LOC107986122, LOC107986167, LOC107986852, LOC107986962, LOC107986968, LOC148413, LOC150051, LOC151760, LOC153684, LOC284023, LOC285505, LOC388780, LOC389332, LOC400499, LOC401052, LOC401320, LOC401397, LOC403323, LOC440934, LOC642515, LOC644249, LOC646730, LOC648987, LOC728485, LOC728752, LOC730183, LOC90768, LOC93622, LONRF3, LPAR1, LPAR2, LPAR4, LPAR5, LPAR6, LPGAT1, LPL, LPP-AS2, LPXN, LRCH1, LRCH4, LRP1, LRP4-AS1, LRPAP1, LRPPRC, LRRC32, LRRC4, LRRC45, LRRC59, LRRC75A-AS1, LRRK2, LRSAM1, LSM2, LSM5, LSM6, LSP1, LSR, LSS, LTBR, LYK5, LYN, LYPD3, LYPLA1, LYZ, LZIC, LZTR1, M6PR, MAD2L1BP, MAEL, MAFB, MAFG, MAGEA10, MAGEA9, MAGEB6, MAGEC2, MAGED1, MAGI2-AS3, MALT1, MAOA, MAOB, MAP2K1, MAP2K3, MAP2K4, MAP2K5, MAP2K6, MAP2K7, MAP3K11, MAP3K12, MAP3K13, MAP3K14, MAP3K14-AS1, MAP3K15, MAP3K2, MAP3K5, MAP3K6, MAP3K7, MAP3K8, MAP3K9, MAP4K1, MAP4K2, MAP4K3, MAP4K4, MAP4K5, MAP7, MAPK1, MAPK12, MAPK13, MAPK14, MAPK15, MAPK1IP1L, MAPK3, MAPK4, MAPK6, MAPK7, MAPK8, MAPK9, MAPKAP1, MAPKAPK2, MAPKAPK3, MAPKAPK5, MARCKS, MARK1, MARS, MAS1, MAS1L, MAST1, MAST2, MAT1A, MAT2A, MAT2B, MATK, MAX, MB, MBD3, MBD4, MBNL1, MBNL1-AS1, MBNL2, MBNL3, MCCC1, MCCC2, MCF2L, MCF2L-AS1, MCL1, MCM2, MCM3, MCM3AP-AS1, MCM5, MCM7, MCM8, MCOLN1, MCTS1, MDH1, MDH2, MDM2, MDM4, MECP2, MED21, MED28, MED4, MED6, MED7, MEF2A, MEF2B, MEF2C, MEF2D, MEG3, MEIS2, MEOX1, MEOX2, MERTK, MESP1, MEST, MET, METTL1, METTL14, METTL21B, METTL4, MET_Y1248C, MET_Y1252C, MEX3D, MFAP4, MFHAS1, MFSD10, MGC16169, MGLL, MGRN1, MGST2, MIEF1, MIF, MINA, MIR100HG, MIR181A2HG, MIR193BHG, MIR31HG, MIR34A, MITF, MKI67IP, MKKS, MKNK1, MKNK2, MKRN2, MKRN3, MKX, MLH1, MLL5, MLLT10, MLLT11, MLLT3, MLLT6, MLST8, MLYCD, MMP1, MMP14, MMP2, MMP24-AS1, MMP25-AS1, MMP7, MNAT1, MNT, MOK, MORF4L1, MORF4L2, MOS, MPHOSPH9, MPO, MPPED1, MPZL1, MRGPRF, MRGPRX3, MRGPRX4, MRPL12, MRPL18, MRPL43, MRPS16, MRPS17, MRPS2, MRPS31, MSH2, MSH5, MSRA, MSRB2, MSX2, MTAP, MTERFD1, MTF2, MTFR1, MTM1, MTOR, MTOR_KIN, MTX2, MUSK, MVP, MXD1, MXD3, MXI1, MXRA8, MYB, MYC, MYCBP, MYCNOS, MYD88, MYF6, MYL6, MYLK-AS1, MYLK2, MYLK3, MYLK4, MYNN, MYO3B, MYOD1, MYOG, MYST2, MYT1, MZT1, NAA25, NAA50, NAB2, NAE1, NAGK, NAMPT, NANOG, NAP1L1, NAP1L3, NARFL, NASP, NAT1, NAT10, NBDY, NBPF15, NCBP2-AS2, NCF2, NCK1-AS1, NCOA1, NCOA2, NCOA3, NCOA4, NDFIP1, NDP, NDRG1, NDUFA1, NDUFA13, NDUFA3, NDUFA4, NDUFA7, NDUFA8, NDUFA9, NDUFAB1, NDUFB1, NDUFB3, NDUFB5, NDUFB6, NDUFB8, NDUFC2, NDUFS3, NDUFS4, NDUFS6, NDUFS7, NDUFV1, NDUFV2, NEDD4L, NEDD8, NEK10, NEK11, NEK2, NEK6, NEK8, NEK9, NENF, NET1, NEU1, NEUROD1, NEUROG1, NF2, NFAT5, NFATC3, NFE2, NFE2L1, NFE2L2, NFIA, NFIB, NFIL3, NFKB1, NFKB2, NFKBIA, NFKBIB, NFKBIE, NFYA, NFYB, NGEF, NISCH, NIT1, NKIRAS2, NKX2-5, NLK, NME1, NMI, NMNAT2, NMNAT3, NMRAL1, NMT1, NMUR2, NNT-AS1, NOC4L, NOD2, NONO, NOSIP, NOTCH1, NOTCH2, NOTCH2NL, NOV, NOX1, NOXA1, NPDC1, NPM1, NPR2, NPRL2, NPSR1, NQO2, NR0B1, NR0B2, NR1D2, NR1H2, NR1H3, NR1H4, NR1I2, NR1I3, NR2C1, NR2C2, NR2E1, NR2E3, NR2F2-AS1, NR2F6, NR3C1, NR4A1, NR4A2, NR5A1, NR5A2, NRAS, NRAV, NRBF2, NRBP1, NRBP2, NRF1, NRG1, NRIP1, NRIP3, NRP1, NSA2, NSDHL, NSFL1C, NT5E, NTAN1, NTRK1, NTRK3, NUAK2, NUDT14, NUDT6, NUDT9, NUP50-AS1, NUP62, NUP88, NUSAP1, NXF1, O3FAR1, OAS1, OAT, OGG1, OLIG3, OMA1, OPA1, OPN3, OPN4, OPN5, OR2G6, OR2M2, OR2T33, OR52B2, ORMDL1, ORMDL2, ORMDL3, OSER1-AS1, OSGEP, OSR2, OTUD3, OTUD6A, OTX1, OTX2, OVCA2, OVOL1, OVOL2, OXCT1, OXER1, OXGR1, OXTR, P2RX4, P2RY12, P2RY2, P2RY6, P2RY8, P4HA2, P4HB, P62-666SQSTM1, PA2G4, PABPC1, PACSIN3, PADI4, PAF1, PAFAH1B1, PAFAH1B2, PAFAH1B3, PAH, PAICS, PAK1, PAK2, PAK3, PAK4, PAK7, PANK2, PANK4, PAPD7, PAPSS1, PAQR7, PAQR8, PARL, PARN, PARP15, PASD1, PAX3, PAX5, PAX6, PAX7, PAX8, PAX8-AS1, PAXIP1-AS2, PBK, PBRM1, PBX2, PBX4, PBXIP1, PC, PCAT19, PCAT7, PCBD1, PCCB, PCDHA10, PCDHGB1, PCGF2, PCGF3, PCGF5, PCGF6, PCK2, PCNA, PCTK3, PDAP1, PDCD1LG2, PDE4D, PDGFC, PDGFRA, PDGFRB, PDGY, PDHA1, PDHB, PDHX, PDIA4, PDIA5, PDIK1L, PDK1, PDK2, PDK4, PDLIM1, PDLIM4, PDPK1, PDS5A, PDS5B, PDXK, PDZD11, PECR, PEMT, PEPD, PER1, PEX11A, PEX13, PEX19, PEX3, PFAS, PFKFB3, PFKL, PFN2, PFTK2, PGBD1, PGD, PGK1, PHACTR1, PHB, PHB2, PHF1, PHF11, PHF13, PHF17, PHF19, PHF23, PHF5A, PHKG2, PHLPP1, PHTF1, PHTF2, PHYH, PI16, PI4KAP2, PIAS1, PIGA, PIGB, PIGBOS1, PIGK, PIH1D1, PIK3CA, PIK3CB, PIK3CD, PIK3CG, PIK3IP1, PIK3R1, PIK3R2, PIK3R3, PIK3R4, PIM1, PIM2, PIM3, PIN1, PINK1, PIP5K3, PIPOX, PISD, PITPNA, PITPNA-AS1, PITX1, PITX2, PJA1, PJA2, PKIA, PKIG, PKM2, PKN1, PKN2, PKNOX1, PLA2G12A, PLA2G12B, PLAT, PLAU, PLAUR, PLCB1, PLCG1, PLCG2, PLD1, PLEKHA4, PLEKHG3, PLEKHG5, PLEKHG6, PLEKHJ1, PLK1, PLK2, PLK4, PLOD1, PLOD3, PLP2, PLXNA4, PMF1-BGLAP, PML, PNCK, PNKP, PNLDC1, PNN, PNP, PNPO, POFUT1, POGZ, POLA2, POLB, POLD1, POLE, POLE2, POLE3, POLE4, POLG2, POLR1C, POLR2C,

	POLR2D, POLR2E, POLR2F, POLR2H, POLR2I, POLR3C, POLR3D, POLR3K, POP4, POT1, POU2AF1, POU2F2, POU4F3, POU5F1, PP14571, PPAP2A, PPAP2B, PPARA, PPARD, PPARG, PPARGC1A, PPAT, PPFIBP2, PPIA, PPIAL4G, PPIE, PPIL2, PPM1B, PPOX, PPP1CA, PPP1CC, PPP1R10, PPP1R13B, PPP1R8, PPP2CB, PPP2R1A, PPP2R3A, PPP2R3C, PPP2R4, PPP2R5C, PPP3CA, PPP3CB, PPP3CC, PPP3R2, PPP4C, PPP6C, PPT1, PQBP1, PRAF2, PRAME, PRB3, PRCC, PRDM1, PRDM10, PRDM14, PRDM4, PRDM5, PRDM7, PRDX2, PRDX5, PREB, PREX2, PRKAA1, PRKAB1, PRKAB2, PRKACA, PRKACB, PRKACG, PRKAG1, PRKAG2, PRKAG2-AS1, PRKAG3, PRKAR1A, PRKAR2A, PRKAR2B, PRKCA, PRKCB, PRKCE, PRKCG, PRKCH, PRKCQ, PRKCZ, PRKD1, PRKD2, PRKG2, PRKR, PRKRIR, PRKX, PRKY, PRMT2, PRMT7, PRNP, PROC, PROCR, PROKR1, PRPF19, PRPF4, PRPF4B, PRPF6, PRR15L, PRR23B, PRR34-AS1, PRR7-AS1, PRSS2, PRSS23, PSAT1, PSEN1, PSEN2, PSENEN, PSG11, PSG2, PSKH1, PSKH2, PSMA1, PSMA3, PSMA3-AS1, PSMA5, PSMA8, PSMB1, PSMB10, PSMB2, PSMB7, PSMB8, PSMB9, PSMC5, PSMD10, PSMD12, PSMD2, PSMD3, PSMD4, PSMD5, PSMD5-AS1, PSMD9, PSME1, PSME2, PSMF1, PSMG1, PSMG3-AS1, PSPH, PTEN, PTGER4, PTGFR, PTGS2, PTHLH, PTK2, PTK2B, PTP4A2, PTP4A3, PTPLA, PTPN12, PTPN2, PTPN4, PTPN6, PTPRF, PTPRG-AS1, PTRF, PTS, PTTG1, PUF60, PURG, PUS7L, PVR, PVRL1, PVRL2, PWP1, PXK, PXN-AS1, PYCR1, PYGL, QDPR, QPCT, QPRT, QRSL1, RAB11A, RAB11B-AS1, RAB11FIP2, RAB11FIP3, RAB1B, RAB23, RAB27A, RAB31, RAB42, RAB4A, RAB5A, RABGEF1, RABGGTA, RAC1, RAD1, RAD18, RAD23B, RAE1, RAF1, RAG1, RAGE, RALA, RALB, RALBP1, RAMP1, RAN, RAP1A, RAP1GDS1, RAPGEF3, RAPGEF4, RAPGEF5, RAPSN, RARA, RARB, RARG, RARRES3, RASA1, RASD1, RASGRP1-V1, RASGRP1-V2, RASGRP2, RASGRP3, RASGRP4, RASSF1, RASSF2, RASSF8-AS1, RB1, RBBP4, RBBP6, RBBP7, RBCK1, RBKS, RBM10, RBM11, RBM14, RBM15, RBM15B, RBM22, RBM26, RBM34, RBM45, RBM5, RBM6, RBMS1, RBMX, RBP4, RBPJ, RBX1, RC3H2, RCBTB2, RCC1, RCCD1, RCHY1, RCOR3, RDBP, RDH11, RDX, REG1A, REG1B, REG3A, REL, RELA, RELB, RELL2, REPIN1, RERG, REST, RET, RET_M918T, RFC5, RFK, RFWD2, RFWD3, RFX3, RFX4, RFX5, RFX6, RFXANK, RG9MTD3, RGR, RGS11, RGS18, RGS3, RGS4, RGS6, RGS7, RGS9, RHEB, RHOA, RHOBTB1, RHOBTB2, RHOC, RHOD, RHOXF1, RHPN1-AS1, RICTOR, RIMS2, RING1, RIOK2, RIOK3, RIPK1, RIPK2, RIPK5, RIT1, RIT2, RLF, RNASE4, RNASET2, RND3, RNF10, RNF11, RNF111, RNF138, RNF14, RNF144A-AS1, RNF145, RNF146, RNF166, RNF167, RNF17, RNF186, RNF19B, RNF2, RNF20, RNF213, RNF219, RNF25, RNF31, RNF40, RNF5, RNF7, RNF8, RNGTT, RNH1, RNMT, RNPS1, ROGDI, RORA, RORC, ROS, RPA2, RPA4, RPF1, RPL11, RPL19, RPL21, RPL22, RPL35A, RPL39L, RPL7, RPN1, RPN2, RPS10, RPS13, RPS14, RPS15A, RPS16, RPS19, RPS27A, RPS3, RPS3A, RPS6, RPS6KA1, RPS6KA2, RPS6KA3, RPS6KA4, RPS6KA5, RPS6KB1, RPS6KB2, RPS6KC1, RPS6KL1, RPS7, RPTOR, RRAGA, RRAGC, RRM1, RRM2, RRP8, RRS1, RSPH1, RSPRY1, RSU1, RTCD1, RTN4RL1, RUFY1, RUVBL1, RUVBL2, RXFP1, RXFP4, RXRA, RXRB, RXRG, RYBP, S100A1, S100A11, S100A13, S100A6, S100B, S100P, S1PR2, S1PR3, SACM1L, SALL4, SAMD4A, SAMD4B, SAMHD1, SARS, SAT1, SATB1, SATB2, SC5DL, SCAF8, SCCPDH, SCEL, SCMH1, SCP2, SCRN1, SCYL3, SDF2L1, SDHA, SDHB, SDHC, SDHD, SENP2, SENP5, SENP6, SEPT4-AS1, SERINC3, SERPINA12, SERPINA5, SERPINA6, SERPINA7, SERPINB2, SERPINB4, SERPINB5, SERPINB6, SERPIND1, SERTAD4-AS1, SET, SETBP1, SETD5, SETD6, SETD7, SETD8, SETDB1, SETDB2, SETMAR, SF1, SF3A3, SFPQ, SGK1, SGK2, SGK3, SGPL1, SH3BGRL2, SH3BP5, SH3RF2, SHC1, SHC4, SHMT2, SHOX2, SHPRH, SIAH1, SIK1, SIM2, SIP1, SIRPG, SIRT1, SIRT2, SIRT3, SIRT4, SIRT5, SIRT6, SIX2, SKAP2, SKP1, SKP2, SLC16A11_D127G, SLC16A11_DBL, SLC16A11_G340S, SLC16A11_L187L, SLC16A11_P443T, SLC16A11_QNT, SLC16A11_REF, SLC16A11_V113I, SLC16A13_REF, SLC1A1, SLC1A5, SLC22A18, SLC22A23, SLC22A4, SLC22A5, SLC25A10, SLC25A13, SLC25A14, SLC25A15, SLC25A22, SLC25A3, SLC25A32, SLC25A4, SLC25A5, SLC25A6, SLC27A3, SLC29A1, SLC2A1, SLC2A3, SLC2A6, SLC30A2, SLC30A8, SLC30A9, SLC35A1, SLC35A4, SLC35F2, SLC37A4, SLC38A2, SLC39A8, SLC3A2, SLC46A1, SLC4A2, SLC5A6, SLC6A14, SLC7A1, SLC7A11, SLC7A9, SLFN11, SLIRP, SLITRK6, SLK, SMAD1, SMAD2, SMAD3, SMAD4, SMAD5, SMAD7, SMAD7|CPS1|SUZ12|FAM5C, SMARCA5, SMARCAD1, SMARCB1, SMARCC1, SMARCC2, SMARCD1, SMARCE1, SMIM25, SMIM26, SMIM27, SMIM30, SMNDC1, SMO, SMOX, SMR3A, SMU1, SMURF2, SMYD1, SMYD3, SMYD4, SNAI1, SNAI2, SNAI3, SNAPC1, SNCA, SNHG1, SNHG10, SNHG15, SNHG17, SNHG18, SNHG21, SNHG25, SNHG3, SNHG5, SNHG6, SNHG8, SNHG9, SNRK, SNRNP25, SNRNP70, SNRPA, SNURF, SNW1, SNX11, SNX17, SNX6, SNX7, SOAT1, SOCS1, SOCS2, SOCS3, SOCS4, SOCS5, SOCS6, SOD1, SORBS2, SORBS3, SORD, SOS1, SOS2, SOX10, SOX14, SOX15, SOX2, SOX2-OT, SOX5, SOX6, SP100, SP110, SP110A, SP110B, SP2, SP2-AS1, SP6, SP8, SPA17, SPAAR, SPAG7, SPAG8, SPANXN4, SPARC, SPATA13, SPATA8, SPDEF, SPECC1L, SPIB, SPIC, SPINT1-AS1, SPOP, SPP1, SPRED2, SPRY1, SPRY2, SPRY4, SPTLC2, SPTY2D1-AS1, SQLE, SQRDL, SQSTM1, SRC, SRD5A1, SREBF1, SRM, SRP14, SRP14-AS1, SRP54-AS1, SRPK1, SRPK2, SRPK3, SRPRB, SRPX, SRSF4, SSBP2, SSBP4, SSX2, SSX3, ST14, ST18, STAG2, STAMBP, STAP2, STAT1, STAT2, STAT3, STAT4, STAT5A, STAT5B, STAT6, STC2, STK11, STK16, STK17A, STK19, STK24, STK25, STK3, STK32C, STK33, STK38, STK38L, STMN1, STRADB, STUB1, STX4, STXBP1, STYK1, SUB1, SUCLA2, SUCNR1, SULT1A1, SULT1A2, SUOX, SUPT4H1, SUPT5H, SUPV3L1, SUV39H1, SUV39H2, SUV420H1, SUZ12, SV2C, SWAP70, SYCP1, SYF2, SYK, SYN2, SYNGR3, SYPL1, SYT1, TACC3, TADA2A, TADA2L, TADA3, TAF12, TAF13, TAF15, TAF1B, TAF5L, TAF6L, TAGAP, TAGAP-A, TAGAP-B, TAGAP-C, TAL2, TANK, TAOK3, TAP1, TAP2, TAPT1-AS1, TAX1BP1, TBC1D3, TBC1D9B, TBCB, TBK1, TBL1XR1, TBX15, TBX2, TBX20, TBX22, TBX3, TBX5-AS1, TCEA1, TCEB3, TCEB3B, TCF12, TCF4, TCF7L2, TCIRG1, TCTA, TCTN1, TDRD3, TEAD2, TEAD4, TERF1, TERF2IP, TERT, TES, TESK1, TESK2, TEX10, TEX11, TFAP2A, TFAP2A-AS1, TFAP4, TFCP2, TFDP1, TFDP2, TFEB, TFF2, TFG, TFPI, TGDS, TGFA, TGFB1, TGFBR1, TGFBR2, TGIF1, TGIF2, TGIF2LY, TGM2, TH, THAP11, THAP5, THAP7-AS1, THAP8, THEMIS, THRA, THRAP3, THRB, THUMPD3-AS1, TICAM2, TIE1, TIGD4, TIGD6, TIGD7, TIMELESS, TIMM17B, TIMM50, TIMM8A, TIMM9, TIMP2, TIMP3, TIMP4, TIRAP, TK1, TKT, TLE1, TLE2, TLK2, TLR2, TLR5, TLR8, TLX2, TLX3, TM7SF2, TMED10, TMED7, TMED9, TMEM110, TMEM154, TMEM174, TMEM5, TMEM97, TMPO, TMSB4X, TNF, TNFAIP1, TNFAIP3, TNFRSF10A, TNFRSF10B, TNFRSF13B, TNFRSF14, TNFRSF17, TNFRSF19, TNFRSF1A, TNFRSF21, TNFRSF6B, TNFSF10, TNFSF13, TNFSF13B, TNFSF8, TNIP1, TNK1, TNK2, TNNI3K, TOLLIP, TOMM22, TOMM34, TOR1A, TOX, TOX2, TOX4, TP53, TP53RK, TP63, TPD52L2, TPD52L3, TPI1, TPK1, TPM1, TPM3, TPMT, TPT1-AS1, TPTE, TRAF1, TRAF2, TRAF3, TRAF3IP2, TRAF3IP2-AS1, TRAF4, TRAF5, TRAF6, TRAFD1, TRAIP, TRAK2, TRAM2, TRAP1, TRAT1, TRHDE-AS1, TRIB1, TRIB3, TRIM13, TRIM16, TRIM17, TRIM2, TRIM21, TRIM22, TRIM23, TRIM26, TRIM27, TRIM29, TRIM32, TRIM36, TRIM37, TRIM38, TRIM39, TRIM41, TRIM42, TRIM46, TRIM50, TRIM55, TRIM56, TRIM60, TRIM62, TRIM7, TRIM73, TRIM9, TRIP10, TRIP12, TRIP13, TRIT1, TRMT1, TSC1, TSC2, TSC22D1, TSC22D3, TSC22D4, TSEN2, TSFM, TSG101, TSN, TSPAN4, TSPAN6, TSPAN8, TSSC4, TSSK1B, TSSK2, TSSK3, TSSK6, TSTA3, TSTD3, TTK, TTN-AS1, TTR, TTTY14, TUBA1A, TUBA3FP, TUBB, TUBB2A, TUBB2C, TUBB3, TUBB6, TUBD1, TULP2, TUT1, TWF2, TWIST2, TXLNA, TXNDC9, TXNIP, TXNL1, TXNL4B, TYK2, TYRO3, U2AF1, UBA52, UBAP1, UBAP2L, UBASH3A-K387N, UBE2A, UBE2D1, UBE2E3, UBE2J1, UBE2K, UBE2L6, UBE2V1, UBE3B, UBE3C, UBE4A, UBL5, UBOX5, UBR1, UBR5-AS1, UCHL1, UCK2, UFD1L, UFM1, UGCG, UGDH, UGDH-AS1, UGP2, UGT1A1, UGT1A6, UGT1A9, UHRF1, ULK2, ULK3, UNC45B, UNC5C, UNC93B1, UNC93B4, UQCR11, UQCRFS1, UROD, USF1, USF2, USP1, USP14, USP15, USP27X-AS1, USP39, USP6NL, UTP14A, UTS2R, UXT, VAMP3, VAPB, VARS, VAV1, VDAC1, VDR, VEGFA, VEGFB, VEGFC, VGLL4, VHL, VIM, VIPR1, VKORC1, VN1R2, VN1R4,

	VN1R5, VPS26A, VPS28, VPS41, VPS72, VPS9D1-AS1, VRK1, VRK2, VSIG10L2, WAC-AS1, WARS, WARS2, WBSCR17, WDR18, WDR5, WDR61, WDTC1, WFDC2, WHSC1L1, WIPI1, WISP2, WNK1, WNK3, WNK4, WNT1, WNT10B, WNT2, WNT5A, WNT7B, WNT9A, WT1, WTAP, WWP1, WWP2, WWTR1, XBP1, XIAP, XK, XKR6, XKR8, XKRX, XLOC_000348, XLOC_000587, XLOC_000889, XLOC_000966, XLOC_001257, XLOC_001272, XLOC_001323, XLOC_001342, XLOC_001398, XLOC_001453, XLOC_001532, XLOC_001537, XLOC_001605, XLOC_001646, XLOC_001728, XLOC_001826, XLOC_002075, XLOC_002111, XLOC_002133, XLOC_002263, XLOC_002344, XLOC_002408, XLOC_002539, XLOC_002736, XLOC_002746, XLOC_002888, XLOC_003204, XLOC_003385, XLOC_003782, XLOC_003870, XLOC_004122, XLOC_004165, XLOC_004250, XLOC_004284, XLOC_004297, XLOC_004323, XLOC_004382, XLOC_004457, XLOC_004698, XLOC_004803, XLOC_004896, XLOC_004924, XLOC_005086, XLOC_005143, XLOC_005219, XLOC_005634, XLOC_005769, XLOC_005777, XLOC_005925, XLOC_005935, XLOC_005945, XLOC_006025, XLOC_006194, XLOC_006301, XLOC_006313, XLOC_006450, XLOC_006513, XLOC_006516, XLOC_006704, XLOC_006894, XLOC_006915, XLOC_006985, XLOC_007053, XLOC_007123, XLOC_007249, XLOC_007458, XLOC_007769, XLOC_007970, XLOC_008151, XLOC_008174, XLOC_008183, XLOC_008370, XLOC_008462, XLOC_008583, XLOC_008704, XLOC_008711, XLOC_008730, XLOC_008823, XLOC_008829, XLOC_009199, XLOC_009233, XLOC_009299, XLOC_009340, XLOC_009549, XLOC_009645, XLOC_009788, XLOC_009810, XLOC_009943, XLOC_010102, XLOC_010202, XLOC_010445, XLOC_010514, XLOC_010813, XLOC_010952, XLOC_011036, XLOC_011064, XLOC_011944, XLOC_012046, XLOC_012281, XLOC_012324, XLOC_012342, XLOC_012505, XLOC_012530, XLOC_012538, XLOC_012542, XLOC_012568, XLOC_012827, XLOC_012975, XLOC_013207, XLOC_013222, XLOC_013282, XLOC_013429, XLOC_013499, XLOC_013615, XLOC_013835, XLOC_013921, XLOC_014105, XLOC_014192, XLOC_014288, XLOC_L2_000101, XLOC_L2_000416, XLOC_L2_000582, XLOC_L2_000636, XLOC_L2_002033, XLOC_L2_002204, XLOC_L2_003877, XLOC_L2_004212, XLOC_L2_004287, XLOC_L2_004598, XLOC_L2_005874, XLOC_L2_006404, XLOC_L2_006648, XLOC_L2_006983, XLOC_L2_006994, XLOC_L2_007033, XLOC_L2_007456, XLOC_L2_007489, XLOC_L2_007543, XLOC_L2_007571, XLOC_L2_008203, XLOC_L2_008692, XLOC_L2_009105, XLOC_L2_009285, XLOC_L2_009655, XLOC_L2_009882, XLOC_L2_010127, XLOC_L2_011173, XLOC_L2_011728, XLOC_L2_011885, XLOC_L2_011983, XLOC_L2_012023, XLOC_L2_012578, XLOC_L2_013314, XLOC_L2_013594, XLOC_L2_013650, XLOC_L2_013963, XLOC_L2_014012, XLOC_L2_014171, XLOC_L2_014585, XLOC_L2_014771, XLOC_L2_015034, XLOC_L2_015037, XLOC_L2_015295, XPA, XPC, XPO1, XPO7, XPR1, XRCC4, XRCC6, YAF2, YAP1, YARS, YEATS4, YES1, YKT6, YTHDC1, YTHDF1, YTHDF2, YWHAQ, YY1, ZAK, ZBED1, ZBED2, ZBED4, ZBED5-AS1, ZBP1, ZBTB1, ZBTB11-AS1, ZBTB17, ZBTB20, ZBTB22, ZBTB24, ZBTB25, ZBTB26, ZBTB37, ZBTB38, ZBTB40, ZBTB43, ZBTB44, ZBTB45, ZBTB46, ZBTB47, ZBTB48, ZBTB49, ZBTB7A, ZBTB7B, ZBTB9, ZC3H7B, ZC3HC1, ZCCHC11, ZCCHC6, ZDHHC11, ZDHHC4, ZEB1, ZEB1-AS1, ZEB2, ZER1, ZFAND6, ZFAS1, ZFP1, ZFP112, ZFP161, ZFP2, ZFP28, ZFP3, ZFP36L1, ZFP91, ZFPM2, ZFYVE19, ZFYVE26, ZGPAT, ZHX1, ZIC3, ZIK1, ZIM3, ZKSCAN1, ZKSCAN2, ZKSCAN3, ZKSCAN4, ZMAT2, ZMYM1, ZNF10, ZNF100, ZNF114, ZNF131, ZNF133, ZNF134, ZNF136, ZNF140, ZNF141, ZNF148, ZNF155, ZNF165, ZNF169, ZNF174, ZNF175, ZNF18, ZNF187, ZNF19, ZNF192, ZNF193, ZNF195, ZNF20, ZNF200, ZNF202, ZNF205, ZNF207, ZNF212, ZNF213, ZNF213-AS1, ZNF217, ZNF219, ZNF22, ZNF222, ZNF223, ZNF224, ZNF227, ZNF23, ZNF232, ZNF238, ZNF239, ZNF24, ZNF248, ZNF253, ZNF256, ZNF257, ZNF26, ZNF263, ZNF266, ZNF267, ZNF268, ZNF273, ZNF274, ZNF276, ZNF277, ZNF280A, ZNF281, ZNF285A, ZNF296, ZNF3, ZNF30, ZNF300, ZNF317, ZNF32, ZNF320, ZNF321, ZNF322A, ZNF323, ZNF329, ZNF331, ZNF333, ZNF335, ZNF34, ZNF343, ZNF345, ZNF350, ZNF354A, ZNF366, ZNF37A, ZNF384, ZNF385A, ZNF385C, ZNF385D, ZNF394, ZNF396, ZNF398, ZNF404, ZNF410, ZNF415, ZNF416, ZNF417, ZNF418, ZNF423, ZNF426, ZNF428, ZNF430, ZNF433, ZNF436, ZNF439, ZNF440, ZNF444, ZNF446, ZNF449, ZNF451, ZNF454, ZNF460, ZNF461, ZNF462, ZNF468, ZNF473, ZNF483, ZNF486, ZNF488, ZNF490, ZNF496, ZNF497, ZNF500, ZNF502, ZNF503, ZNF503-AS2, ZNF509, ZNF510, ZNF512, ZNF513, ZNF517, ZNF518A, ZNF519, ZNF521, ZNF524, ZNF525, ZNF529, ZNF529-AS1, ZNF530, ZNF536, ZNF543, ZNF544, ZNF546, ZNF548, ZNF549, ZNF550, ZNF551, ZNF554, ZNF555, ZNF556, ZNF557, ZNF558, ZNF559, ZNF561, ZNF562, ZNF563, ZNF564, ZNF567, ZNF569, ZNF57, ZNF570, ZNF571, ZNF572, ZNF573, ZNF576, ZNF577, ZNF581, ZNF582, ZNF583, ZNF585A, ZNF587, ZNF592, ZNF595, ZNF596, ZNF597, ZNF599, ZNF607, ZNF608, ZNF610, ZNF619, ZNF621, ZNF622, ZNF625, ZNF626, ZNF627, ZNF639, ZNF645, ZNF649, ZNF653, ZNF655, ZNF658, ZNF669, ZNF670, ZNF671, ZNF672, ZNF673, ZNF677, ZNF679, ZNF680, ZNF689, ZNF69, ZNF692, ZNF695, ZNF697, ZNF70, ZNF701, ZNF707, ZNF71, ZNF711, ZNF718, ZNF726-1.5, ZNF738, ZNF74, ZNF747, ZNF75D, ZNF764, ZNF768, ZNF77, ZNF770, ZNF772, ZNF774, ZNF785, ZNF786, ZNF788, ZNF79, ZNF790, ZNF790-AS1, ZNF791, ZNF8, ZNF808, ZNF821, ZNF827, ZNF83, ZNF830, ZNF85, ZNF92, ZNF98, ZNFX1-AS1, ZNHIT3, ZPBP2, ZRANB2, ZRSR1, ZRSR2, ZSCAN1, ZSCAN12, ZSCAN16-AS1, ZSCAN2, ZSCAN21, ZSCAN22, ZSCAN29, ZSCAN5A, ZSWIM2, ZUFSP, ZXDC, ZZZ3

Supplementary Table S3: Cell line list with missing rates below 90%.

11 and 10 cells were used for gene knockdown and gene overexpression signatures, respectively.

Type of gene perturbation	Cell lines
Gene knockdown	A375, A549, ASC, HA1E, HCC515, HEPG2, HT29, MCF7, NPC, PC3, VCAP
Gene overexpression	A375, A549, HA1E, HCC515, HEK293T, HEPG2, HT29, MCF7, PC3, VCAP

Supplementary Table S4: All diseases of disease/patient-specific transcriptome profiles.

In total, we constructed disease/patient-specific transcriptome profiles for 79 diseases and 14,804 genes.

**Disease_name**
Acute myeloid leukemia
Chronic myeloid leukemia
Chronic lymphocytic leukemia
Adult T-cell leukemia
Multiple myeloma
Small cell lung cancer
Gastric cancer
Pancreatic cancer
Colorectal cancer
Renal cell carcinoma
Testicular cancer
Endometrial cancer
Ovarian cancer
Cervical cancer
Breast cancer
Melanoma
Nasopharyngeal cancer
Alzheimer disease; Dementia due to Alzheimer disease
Parkinson disease
Amyotrophic lateral sclerosis (ALS); Lou Gehrig disease
Huntington disease
Lewy body dementia (LBD); Dementia with Lewy bodies (DLB)
Asthma
Systemic lupus erythematosus
Chronic granulomatous disease
Familial combined hyperlipidemia
Familial hypercholesterolemia; Autosomal dominant hypercholesterolaemia
Adrenoleukodystrophy
Neonatal adrenoleukodystrophy
Peroxisome biogenesis disorder
Cystic fibrosis
Sickle cell anemia
Diamond-Blackfan anemia
Ebola disease; Ebola hemorrhagic fever
Crohn disease
Dilated cardiomyopathy
Tuberculosis
Dengue
Mosquito-borne viral fever
Severe acute respiratory syndrome; SARS
Type 1 diabetes mellitus
Type 2 diabetes mellitus
Hepatitis C; Hepatitis C virus (HCV) infection
Rett syndrome
Fragile X syndrome
Mitochondrial complex I deficiency
X-linked mental retardation
Chromosome Xp21 deletion syndrome
Dystrophinopathies
Myotonic dystrophy
Epidermolysis bullosa simplex

Congenital muscular dystrophies (CMD/MDC)
Facioscapulohumeral muscular dystrophy
Distal myopathy
Nonaka distal myopathy (NM); Distal myopathy with rimmed vacuoles (DMRV); Hereditary inclusion body myopathy (hIBM)
46,XY disorder of sex development due to testosterone secretion defect
Primary open angle glaucoma; Glaucoma 1
Rheumatoid arthritis
Marfan syndrome
Primary dystonia
Spastic quadriplegic cerebral palsy
Pituitary adenomas
Alpha-1-antitrypsin deficiency
Aplastic anemia
Left ventricular noncompaction
Ketosis-prone diabetes mellitus
Inflammatory bowel disease (IBD)
Inclusion body myopathy 3
Immune thrombocytopenia; Autoimmune thrombocytopenic purpura
Idiopathic pulmonary fibrosis
Sarcoidosis, early-onset
MELAS Syndrome; Mitochondrial myopathy, Encephalopathy, Lactic Acidosis, Stroke-like episodes
Allergic contact dermatitis
Atopic dermatitis
Hyperlipoproteinemia type IIa; LDL receptor disorder
Alpers syndrome
Autosomal recessive progressive external ophthalmoplegia
Williams-Beuren syndrome
Ulcerative colitis

Supplementary Table S5: Diseases used in gold standard dataset.

These diseases had at least one inhibitory target protein. In total, 32 diseases had at least one inhibitory target protein.

Diseases
Acute myeloid leukemia
Adult T-cell leukemia
Alzheimer disease; Dementia due to Alzheimer disease
Aplastic anemia
Asthma
Atopic dermatitis
Breast cancer
Cervical cancer
Chronic lymphocytic leukemia
Chronic myeloid leukemia
Colorectal cancer
Congenital muscular dystrophies (CMD/MDC)
Crohn disease
Endometrial cancer
Familial hypercholesterolemia; Autosomal dominant hypercholesterolaemia
Gastric cancer
Hepatitis C; Hepatitis C virus (HCV) infection
Idiopathic pulmonary fibrosis
Inflammatory bowel disease (IBD)
Melanoma
Multiple myeloma
Ovarian cancer
Pancreatic cancer
Parkinson disease
Renal cell carcinoma
Rheumatoid arthritis
Small cell lung cancer
Systemic lupus erythematosus
Testicular cancer
Tuberculosis
Type 1 diabetes mellitus
Type 2 diabetes mellitus

Supplementary Table S6: Diseases used in gold standard dataset.

These diseases had at least one activatory target protein. In total, 16 diseases had at least one activatory target protein.

**Diseases**
Acute myeloid leukemia
Aplastic anemia
Breast cancer
Chronic granulomatous disease
Chronic myeloid leukemia
Colorectal cancer
Crohn disease
Inflammatory bowel disease (IBD)
Multiple myeloma
Ovarian cancer
Parkinson disease
Renal cell carcinoma
Rett syndrome
Rheumatoid arthritis
Type 1 diabetes mellitus
Type 2 diabetes mellitus

Supplementary Table S7: Diseases used in uncharacterized disease dataset.

These diseases had at least one inhibitory target protein. In total, 6 diseases had at least one inhibitory target protein.

**Diseases**
Amyotrophic lateral sclerosis (ALS)
Immune thrombocytopenia; Autoimmune thrombocytopenic purpura
Lewy body dementia (LBD); Dementia with Lewy bodies (DLB)
Pituitary adenomas
Primary open angle glaucoma; Glaucoma 1
Ulcerative colitis

Supplementary Table S8: Diseases used in uncharacterized disease dataset.

These diseases had at least one activatory target protein. In total, 16 diseases had at least one activatory target protein.

**Diseases**
Allergic contact dermatitis
Alzheimer disease; Dementia due to Alzheimer disease
Amyotrophic lateral sclerosis (ALS)
Asthma
Atopic dermatitis
Chronic lymphocytic leukemia
Familial hypercholesterolemia; Autosomal dominant hypercholesterolaemia
Hepatitis C; Hepatitis C virus (HCV) infection
Huntington disease
Hyperlipoproteinemia type IIa; LDL receptor disorder
Idiopathic pulmonary fibrosis
Immune thrombocytopenia; Autoimmune thrombocytopenic purpura
Melanoma
Pancreatic cancer
Primary open angle glaucoma; Glaucoma 1
Ulcerative colitis

Supplementary Table S9: Proteins used in uncharacterized protein dataset.

These proteins had at least one known inhibitory or activatory association with disease. In total, 63 and 21 proteins had at least one inhibitory and activatory association with diseases, respectively.

Target type	Uncharacterized proteins
Inhibitory target	ADRB2, AKT2, AKT3, ANPEP, APP, CA12, CA2, CDK7, CTGF, CYP3A5, DDC, DERL1, DNMT1, DNMT3A, EIF2AK4, ELOVL6, EPAS1, FGF2, FUS, GFPT1, GPR84, HPRT1, IFNAR2, IGF1, IL13RA1, IL18, IL1R2, IL8, IRF5, KLF5, LGALS3, LRRK2, MAP3K5, MAPK10, MAPK8, MAPK9, MMP2, NT5E, OXGR1, PARP2, PCSK9, PGF, PIK3CA, PIK3CB, PIK3CD, PRKCB, PRLR, PSEN1, PSEN2, ROCK1, ROCK2, RRM2, SMAD7, SNCA, SOCS3, SOD1, TARDBP, TGM2, TLR7, TLR8, TLR9, TNFRSF18, TNFSF13
Activatory target	ADCY3, ADCY6, ADCY9, ADORA2A, ADRB2, CHRM3, GABBR1, HTR4, NR3C1, PINK1, PML, PPARA, PPARD, PRKAA1, PTGFR, RAP1A, RARA, RARB, RARG, SREBF1, TERT

Supplementary Table S10: Performance evaluation of predicting inhibitory indications for uncharacterized proteins.

Comparison of the performance of proposed and baseline methods for predicting inhibitory targets for 24 diseases and 63 proteins; the proposed method corresponds to SSL-VQ; the baseline methods correspond to SL-VQ, SNP-PV, SNP-eQTL, and multitask learning method. Models were trained and evaluated based on a "uncharacterized protein" scenario (Methods).

Gene	SNP-PV	SNP-eQTL	Multitask	SL-VQ	SSL-VQ
ADRB2	0.500	0.500	0.069	0.500	**0.750**
AKT2	0.500	0.500	0.667	0.722	**0.778**
AKT3	0.500	0.500	**0.806**	0.667	0.611
ANPEP	0.500	0.500	**0.778**	0.667	0.667
APP	**1.000**	0.500	0.472	0.306	0.556
CA12	0.486	0.500	0.069	0.444	**0.722**
CA2	0.500	0.500	0.069	0.389	**0.583**
CDK7	**0.500**	**0.500**	0.306	0.389	0.083
CTGF	0.500	0.500	0.639	0.722	**0.778**
CYP3A5	0.500	0.500	**0.583**	0.000	0.250
DDC	0.458	0.500	0.472	0.194	**0.528**
DERL1	0.500	0.500	0.069	**0.528**	0.222
DNMT1	0.500	0.500	0.639	0.778	**0.806**
DNMT3A	0.431	0.514	0.722	**0.806**	0.694
EIF2AK4	0.486	0.500	**0.778**	0.722	0.556
ELOVL6	0.500	0.500	0.306	**0.528**	0.472
EPAS1	0.486	0.500	0.556	0.083	**0.528**
FGF2	0.500	0.597	**0.806**	0.694	0.611
FUS	0.500	0.500	0.083	**0.556**	0.139
GFPT1	0.500	0.500	**1.000**	0.389	0.556
GPR84	0.500	0.500	**0.722**	0.694	0.694
HPRT1	0.500	0.500	0.514	0.814	**0.829**
IFNAR2	0.500	0.500	**1.000**	0.472	0.778
IGF1	0.486	**0.500**	0.500	0.250	**0.500**
IL13RA1	0.486	**0.500**	0.471	0.000	0.286
IL18	0.500	0.500	**0.671**	0.629	0.000
IL1R2	0.500	0.500	0.750	**0.944**	0.972
IL8	**0.500**	**0.500**	0.472	0.000	0.083
IRF5	**1.000**	0.889	0.861	0.833	0.778
KLF5	**1.000**	0.500	0.694	0.778	0.889

LGALS3	0.500	0.500	0.722	**0.778**	0.694
LRRK2	**1.000**	**1.000**	0.750	0.389	0.472
MAP3K5	**0.500**	**0.500**	0.069	**0.500**	0.083
MAPK10	0.486	0.500	0.778	**0.806**	0.694
MAPK8	0.500	0.500	0.500	**0.694**	**0.694**
MAPK9	0.500	0.500	0.722	**0.806**	0.639
MMP2	0.486	0.500	**1.000**	0.833	0.917
NT5E	0.500	0.500	0.857	0.871	**0.943**
OXGR1	**0.500**	**0.500**	0.250	0.000	0.278
PARP2	0.500	0.500	**0.814**	0.627	0.725
PCSK9	0.500	**0.514**	0.417	0.417	0.250
PGF	0.500	0.500	**1.000**	0.778	0.833
PIK3CA	0.500	0.500	**0.806**	0.722	0.694
PIK3CB	0.500	0.500	**0.833**	0.750	0.778
PIK3CD	0.472	0.500	0.500	**0.889**	0.722
PRKCB	0.403	0.444	0.639	**0.750**	0.667
PRLR	0.500	0.500	0.139	**0.556**	0.361
PSEN1	0.500	0.500	0.306	0.222	**0.528**
PSEN2	0.472	**0.500**	0.389	0.111	0.167
ROCK1	**0.500**	**0.500**	0.279	0.371	0.386
ROCK2	0.500	0.500	0.417	0.490	**0.549**
RRM2	0.500	0.500	**1.000**	0.694	0.611
SMAD7	0.444	**0.500**	0.194	0.181	0.472
SNCA	**1.000**	0.500	0.450	0.686	0.629
SOCS3	**0.500**	**0.500**	0.444	0.000	0.250
SOD1	**1.000**	0.500	0.069	0.611	0.194
TARDBP	0.500	0.500	0.069	**0.583**	0.250
TGM2	0.500	0.500	**0.944**	0.778	0.667
TLR7	0.500	0.500	**0.778**	0.722	0.639
TLR8	0.500	0.500	**0.556**	0.528	0.472
TLR9	0.500	0.500	0.417	0.528	**0.778**
TNFRSF18	0.500	0.556	**0.944**	0.056	0.389
TNFSF13	0.486	0.500	**0.861**	0.472	0.778

Supplementary Table S11: Performance evaluation of predicting activatory indications for uncharacterized proteins.

Comparison of the performance of proposed and baseline methods for predicting activatory targets for 18 diseases and 21 proteins; the proposed method corresponds to SSL-VQ; the baseline methods correspond to SL-VQ, SNP-PV, SNP-eQTL, and multitask learning method. Models were trained and evaluated based on a "uncharacterized protein" scenario (Methods).

gene	SNP-PV	SNP-eQTL	Multitask	SL-VQ	SSL-VQ
ADCY3	0.419	0.484	0.242	0.000	**0.548**
ADCY6	0.500	0.500	0.274	0.000	**0.581**
ADCY9	0.468	0.500	0.242	0.000	**0.548**
ADORA2A	0.500	0.500	0.233	**0.717**	0.683
ADRB2	0.500	0.500	0.525	**0.617**	0.517
CHRM3	0.484	0.500	0.242	0.000	**0.548**
GABBR1	0.468	0.500	0.242	**0.742**	0.387
HTR4	0.500	0.500	0.242	**0.903**	0.710
NR3C1	0.477	0.500	0.382	0.320	**0.514**
PINK1	0.500	0.500	**0.935**	0.452	0.323
PML	0.484	0.500	**0.677**	0.129	0.226
PPARA	0.484	0.500	0.242	**0.532**	0.435
PPARD	0.484	0.500	0.242	**0.532**	0.452
PRKAA1	0.484	0.500	**1.000**	**1.000**	**1.000**
PTGFR	0.484	0.500	0.242	0.000	**0.548**
RAP1A	0.435	0.500	**1.000**	**1.000**	**1.000**
RARA	0.500	0.516	**0.774**	0.161	0.161
RARB	0.403	0.500	**0.839**	0.129	0.161
RARG	0.500	0.500	**0.806**	0.161	0.194
SREBF1	0.435	0.468	0.242	**0.532**	0.403
TERT	**0.935**	0.500	0.242	0.677	0.419

Supplementary Table S12: Newly predicted inhibitory targets for uncharacterized diseases.

We comprehensively predicted new inhibitory targets for all protein–disease pairs (343,255 pairs involving 4,345 proteins and 79 diseases) by SSL-VQ. Because the number of protein–disease pairs is enormous, we listed the results of dilated cardiomyopathy (DCM), amyotrophic lateral sclerosis (ALS), and Rett syndrome (RTT).

Rank	Disease name	Gene	Score
1	Dilated cardiomyopathy	BUB1B	0.910
2	Dilated cardiomyopathy	MST4	0.431
3	Dilated cardiomyopathy	CEBPA	0.147
4	Dilated cardiomyopathy	HDHD1	0.109
5	Dilated cardiomyopathy	F10	0.051
6	Dilated cardiomyopathy	STK10	0.043
7	Dilated cardiomyopathy	HOXA10	0.021
8	Dilated cardiomyopathy	ACSL6	0.014
9	Dilated cardiomyopathy	APOM	0.013
10	Dilated cardiomyopathy	UBE4A	0.008
11	Dilated cardiomyopathy	RPS3A	0.007
12	Dilated cardiomyopathy	TBX2	0.007
13	Dilated cardiomyopathy	BCL9	0.006
14	Dilated cardiomyopathy	CIRBP	0.006
15	Dilated cardiomyopathy	KDR	0.005
16	Dilated cardiomyopathy	MAP3K5	0.005
17	Dilated cardiomyopathy	BAG6	0.004
18	Dilated cardiomyopathy	RPP38	0.004
19	Dilated cardiomyopathy	MAP3K3	0.002
20	Dilated cardiomyopathy	STK19	0.001
21	Dilated cardiomyopathy	SMURF2	0.001
22	Dilated cardiomyopathy	GPR110	0.001
23	Dilated cardiomyopathy	DYRK4	0.001
24	Dilated cardiomyopathy	NDUFA3	0.001
25	Dilated cardiomyopathy	PTCH1	0.000
26	Dilated cardiomyopathy	NPRL2	0.000
27	Dilated cardiomyopathy	RBM8A	0.000
28	Dilated cardiomyopathy	IL13	0.000
29	Dilated cardiomyopathy	ECSIT	0.000
30	Dilated cardiomyopathy	ATOX1	0.000
1	Rett syndrome	BUB1B	1.000
2	Rett syndrome	STK10	1.000
3	Rett syndrome	CEBPA	0.999
4	Rett syndrome	MST4	0.999
5	Rett syndrome	RPP38	0.996
6	Rett syndrome	F10	0.996
7	Rett syndrome	IL13	0.995
8	Rett syndrome	UBE4A	0.994

9	Rett syndrome	ACSL6	0.993
10	Rett syndrome	APOM	0.991
11	Rett syndrome	HDHD1	0.990
12	Rett syndrome	HOXA10	0.989
13	Rett syndrome	NPRL2	0.989
14	Rett syndrome	ATOX1	0.988
15	Rett syndrome	MAP3K5	0.987
16	Rett syndrome	BAG6	0.984
17	Rett syndrome	BCL9	0.981
18	Rett syndrome	MAP3K3	0.977
19	Rett syndrome	SMURF2	0.976
20	Rett syndrome	CIRBP	0.970
21	Rett syndrome	RPS3A	0.966
22	Rett syndrome	UGT1A9	0.963
23	Rett syndrome	NDUFA3	0.954
24	Rett syndrome	STK19	0.953
25	Rett syndrome	RALB	0.951
26	Rett syndrome	GPR110	0.949
27	Rett syndrome	TBX2	0.948
28	Rett syndrome	INPP4B	0.943
29	Rett syndrome	LOC442558	0.942
30	Rett syndrome	DUT	0.940
1	Amyotrophic lateral sclerosis (ALS)	MST4	1.000
2	Amyotrophic lateral sclerosis (ALS)	F10	1.000
3	Amyotrophic lateral sclerosis (ALS)	RPP38	0.999
4	Amyotrophic lateral sclerosis (ALS)	HOXA10	0.997
5	Amyotrophic lateral sclerosis (ALS)	GPR110	0.996
6	Amyotrophic lateral sclerosis (ALS)	EWSR1	0.996
7	Amyotrophic lateral sclerosis (ALS)	CEBPA	0.995
8	Amyotrophic lateral sclerosis (ALS)	RNASET2	0.994
9	Amyotrophic lateral sclerosis (ALS)	CIRBP	0.990
10	Amyotrophic lateral sclerosis (ALS)	CAPG	0.978
11	Amyotrophic lateral sclerosis (ALS)	APOM	0.977
12	Amyotrophic lateral sclerosis (ALS)	GLUD1	0.909
13	Amyotrophic lateral sclerosis (ALS)	SOS1	0.822
14	Amyotrophic lateral sclerosis (ALS)	RPS3A	0.818
15	Amyotrophic lateral sclerosis (ALS)	YAF2	0.776
16	Amyotrophic lateral sclerosis (ALS)	AKAP13	0.505
17	Amyotrophic lateral sclerosis (ALS)	HDHD1	0.474
18	Amyotrophic lateral sclerosis (ALS)	TFCP2	0.454
19	Amyotrophic lateral sclerosis (ALS)	CALCOCO2	0.397
20	Amyotrophic lateral sclerosis (ALS)	SDF2L1	0.356
21	Amyotrophic lateral sclerosis (ALS)	STK19	0.308
22	Amyotrophic lateral sclerosis (ALS)	FAM134C	0.295
23	Amyotrophic lateral sclerosis (ALS)	BCL9	0.204
24	Amyotrophic lateral sclerosis (ALS)	TBX2	0.121

25	Amyotrophic lateral sclerosis (ALS)	CDK6	0.089
26	Amyotrophic lateral sclerosis (ALS)	ZNF432	0.078
27	Amyotrophic lateral sclerosis (ALS)	PSMA8	0.071
28	Amyotrophic lateral sclerosis (ALS)	ACSL6	0.070
29	Amyotrophic lateral sclerosis (ALS)	PGK1	0.065
30	Amyotrophic lateral sclerosis (ALS)	LONRF2	0.047

Supplementary Table S13: Newly predicted activatory targets for uncharacterized diseases.

We comprehensively predicted new activatory targets for all protein–disease pairs (319,160 pairs involving 4,040 proteins and 79 diseases) by SSL-VQ. Because the number of protein–disease pairs is enormous, we listed the results of Huntington disease (HD), amyotrophic lateral sclerosis (ALS), and systemic lupus erythematosus (SLE).

Rank	Disease name	Gene	Score
1	Huntington disease	CCND3	0.00686
2	Huntington disease	IL2	0.00155
3	Huntington disease	ARHGEF19	0.00133
4	Huntington disease	SQRDL	0.00009
5	Huntington disease	RNF10	0.00002
6	Huntington disease	PLK1	0.00002
7	Huntington disease	XLOC_002888	0.00002
8	Huntington disease	PRNP	0.00002
9	Huntington disease	NKIRAS2	0.00001
10	Huntington disease	FRS2	0.00000
11	Huntington disease	ZNF285A	0.00000
12	Huntington disease	CCNC	0.00000
13	Huntington disease	HLA-DRB3	0.00000
14	Huntington disease	RHOBTB1	0.00000
15	Huntington disease	ZNF165	0.00000
16	Huntington disease	RNF20	0.00000
17	Huntington disease	XLOC_002075	0.00000
18	Huntington disease	BAG6	0.00000
19	Huntington disease	TRAF3	0.00000
20	Huntington disease	XLOC_L2_007033	0.00000
21	Huntington disease	MECP2	0.00000
22	Huntington disease	MFAP4	0.00000
23	Huntington disease	TIMP2	0.00000
24	Huntington disease	AK1	0.00000
25	Huntington disease	CISD1	0.00000
26	Huntington disease	ACTN2	0.00000
27	Huntington disease	CHEK1	0.00000
28	Huntington disease	GPR87	0.00000
29	Huntington disease	XLOC_013429	0.00000
30	Huntington disease	MARK1	0.00000
1	Amyotrophic lateral sclerosis (ALS); Lou Gehrig disease	CCND3	0.99378
2	Amyotrophic lateral sclerosis (ALS); Lou Gehrig disease	ARHGEF19	0.73193
3	Amyotrophic lateral sclerosis (ALS); Lou Gehrig disease	IL2	0.69863
4	Amyotrophic lateral sclerosis (ALS); Lou Gehrig disease	SQRDL	0.04483
5	Amyotrophic lateral sclerosis (ALS); Lou Gehrig disease	RNF10	0.00314
6	Amyotrophic lateral sclerosis (ALS); Lou Gehrig disease	XLOC_002888	0.00298
7	Amyotrophic lateral sclerosis (ALS); Lou Gehrig disease	PLK1	0.00293
8	Amyotrophic lateral sclerosis (ALS); Lou Gehrig disease	PRNP	0.00214

9	Amyotrophic lateral sclerosis (ALS); Lou Gehrig disease	NKIRAS2	0.00106
10	Amyotrophic lateral sclerosis (ALS); Lou Gehrig disease	FRS2	0.00052
11	Amyotrophic lateral sclerosis (ALS); Lou Gehrig disease	ZNF285A	0.00042
12	Amyotrophic lateral sclerosis (ALS); Lou Gehrig disease	CCNC	0.00033
13	Amyotrophic lateral sclerosis (ALS); Lou Gehrig disease	TRAF3	0.00029
14	Amyotrophic lateral sclerosis (ALS); Lou Gehrig disease	RNF20	0.00026
15	Amyotrophic lateral sclerosis (ALS); Lou Gehrig disease	ZNF165	0.00024
16	Amyotrophic lateral sclerosis (ALS); Lou Gehrig disease	HLA-DRB3	0.00023
17	Amyotrophic lateral sclerosis (ALS); Lou Gehrig disease	RHOBTB1	0.00022
18	Amyotrophic lateral sclerosis (ALS); Lou Gehrig disease	BAG6	0.00019
19	Amyotrophic lateral sclerosis (ALS); Lou Gehrig disease	MECP2	0.00013
20	Amyotrophic lateral sclerosis (ALS); Lou Gehrig disease	XLOC_L2_007033	0.00013
21	Amyotrophic lateral sclerosis (ALS); Lou Gehrig disease	MFAP4	0.00013
22	Amyotrophic lateral sclerosis (ALS); Lou Gehrig disease	XLOC_002075	0.00012
23	Amyotrophic lateral sclerosis (ALS); Lou Gehrig disease	AK1	0.00004
24	Amyotrophic lateral sclerosis (ALS); Lou Gehrig disease	CISD1	0.00004
25	Amyotrophic lateral sclerosis (ALS); Lou Gehrig disease	GPR87	0.00004
26	Amyotrophic lateral sclerosis (ALS); Lou Gehrig disease	XLOC_013429	0.00004
27	Amyotrophic lateral sclerosis (ALS); Lou Gehrig disease	CHEK1	0.00003
28	Amyotrophic lateral sclerosis (ALS); Lou Gehrig disease	TIMP2	0.00003
29	Amyotrophic lateral sclerosis (ALS); Lou Gehrig disease	ACTN2	0.00003
30	Amyotrophic lateral sclerosis (ALS); Lou Gehrig disease	MYCNOS	0.00002
1	Systemic lupus erythematosus	CCND3	0.92235
2	Systemic lupus erythematosus	IL2	0.10250
3	Systemic lupus erythematosus	ARHGEF19	0.04672
4	Systemic lupus erythematosus	SQRDL	0.01408
5	Systemic lupus erythematosus	RNF10	0.01283
6	Systemic lupus erythematosus	PLK1	0.00754
7	Systemic lupus erythematosus	XLOC_002888	0.00631
8	Systemic lupus erythematosus	NKIRAS2	0.00582
9	Systemic lupus erythematosus	TRAF3	0.00366
10	Systemic lupus erythematosus	FRS2	0.00366
11	Systemic lupus erythematosus	BAG6	0.00231
12	Systemic lupus erythematosus	MFAP4	0.00219
13	Systemic lupus erythematosus	PRNP	0.00207
14	Systemic lupus erythematosus	CCNC	0.00192
15	Systemic lupus erythematosus	AK1	0.00178
16	Systemic lupus erythematosus	XLOC_L2_007033	0.00160
17	Systemic lupus erythematosus	RNF20	0.00141
18	Systemic lupus erythematosus	RHOBTB1	0.00109
19	Systemic lupus erythematosus	XLOC_013429	0.00101
20	Systemic lupus erythematosus	MECP2	0.00098
21	Systemic lupus erythematosus	ACTN2	0.00097
22	Systemic lupus erythematosus	CHEK1	0.00095
23	Systemic lupus erythematosus	ZNF285A	0.00088
24	Systemic lupus erythematosus	CISD1	0.00087

25	Systemic lupus erythematosus	XLOC_002075	0.00087
26	Systemic lupus erythematosus	DEPDC1	0.00070
27	Systemic lupus erythematosus	ZNF165	0.00061
28	Systemic lupus erythematosus	TUBB2A	0.00052
29	Systemic lupus erythematosus	CMAP-ERGK004	0.00048
30	Systemic lupus erythematosus	HLA-DRB3	0.00043

Supplementary Table S14: The number of unlabeled and labeled samples used to assess of the influence of amount of unlabeled samples.

For inhibitory and activatory target predictions, three types of datasets with varying amount of unlabeled samples—“Sample,” “Middle,” and “Large”—are listed. Numbers in brackets indicate ratios of unlabeled or labeled samples.

	**Inhibitory target predictions**		**Activatory target predictions**	
	Unlabeled samples	Labeled samples	Unlabeled samples	Labeled samples
Small dataset	10,575 (59.5 %)	7,200 (40.5%)	2,331 (79.7%)	592 (20.3%)
Middle dataset	131,840 (94.8 %)	7,200 (5.2%)	64,048 (99.1 %)	592 (0.9%)
Large dataset	142,597 (95.2 %)	7,200 (4.8%)	66,379 (99.1 %)	592 (0.9%)

Supplementary Table S15: Training times of SSL-VQ for each parameter set.

The time taken to train SSL-VQ model is shown for each latent layer sizes and drop rate. Note that these calculations were performed on the following computational resources: [iMac: Processor, 3.6 GHz 10 core Inter Core i9; Computer memory, 128 GB 2267 MHz DDR4; OS, Mac OS (Sonoma 14.4.1 Sonoma 14.4.1)].

**Laten layer sizes**	**Drop rate**	**Training time of inhibitory target prediction [min]**	**Training time of activatory target prediction [min]**
[1024,512,256]	0.1	67 min	13 min
[1024,512,256]	0.2	48 min	13 min
[1024,512,256]	0.3	59 min	12 min
[1024,512,256]	0.4	45 min	14 min
[1024,512,256]	0.5	86 min	12 min
